# Supplementary material for: Carbon nitride photocatalyzes regioselective aminium radical addition to the carbonyl bond and yields N-fused pyrroles
Source: Nat Commun. 2019 Feb 26;10:945. doi: 10.1038/s41467-019-08652-w (PMC6391478; doi:10.1038/s41467-019-08652-w)
Supplement: Supplementary file 1 — Supplementary Information [file 41467_2019_8652_MOESM1_ESM.pdf]

Carbon Nitride Photocatalyzes Regioselective Aminium Radical Addition to the Carbonyl Bond and Yields *N*-fused Pyrroles

Kurpil et al.

Supplementary Figures

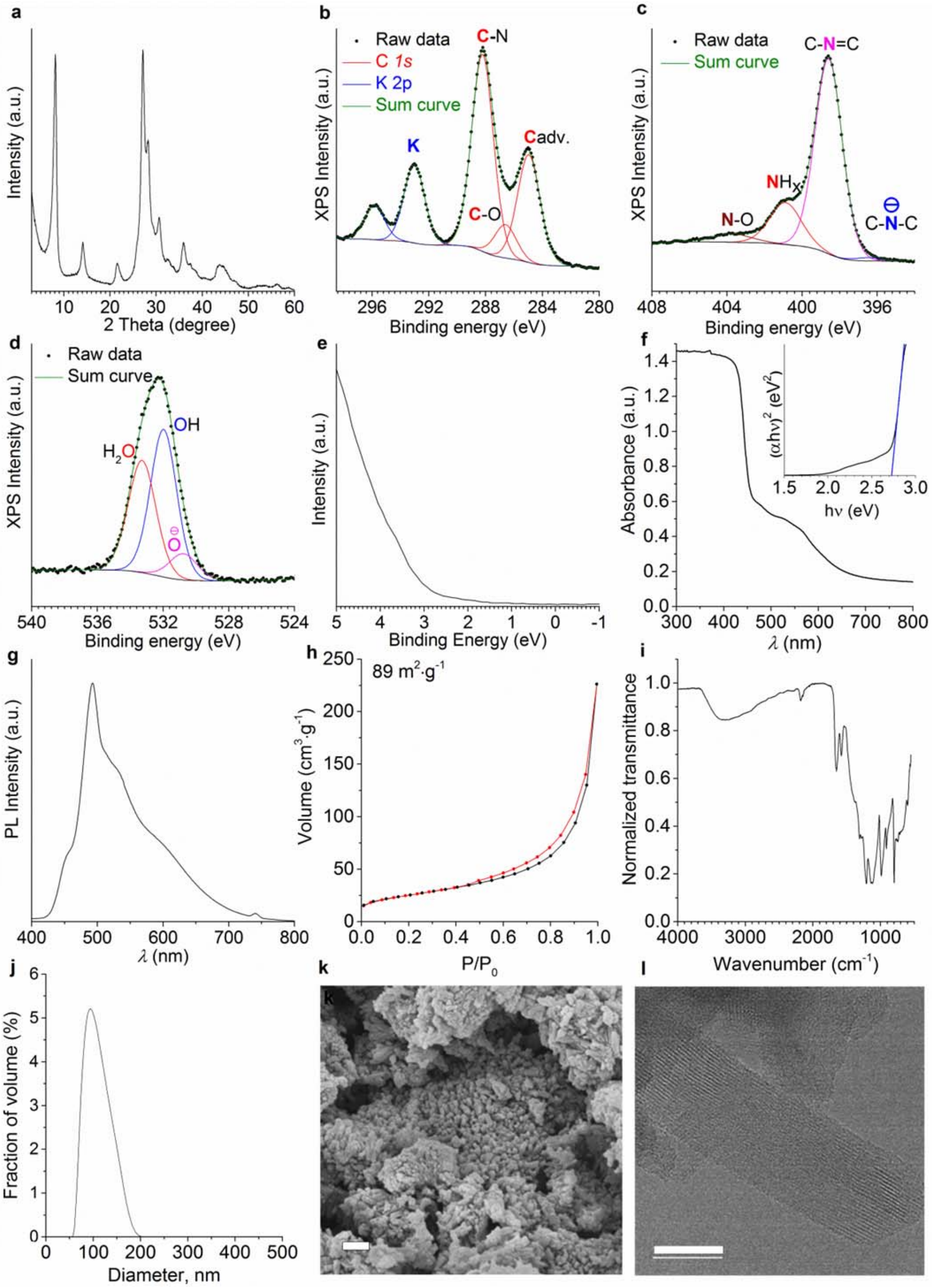

Supplementary Figure 1. K-PHI characterization data. a) PXRD pattern of K-PHI; b) XPS C 1s and K 2p spectra of K-PHI; c) XPS N 1s spectrum of K-PHI; d) XPS O 1s spectrum of K-PHI; e) UPS spectrum of K-PHI; f) UV-vis absorption spectrum of K-PHI with Tauc plot as inset assuming that K-PHI is a direct semiconductor; g) room temperature PL spectrum of K-PHI obtained upon excitation with 350 nm wavelength; h) N_2 sorption isotherm measured at 77 K. BET surface area; i) FT-IR spectrum of K-PHI; j) DLS analysis of K-PHI suspension in water; k) representative SEM image of K-PHI photocatalsyt. Scale bar 200 nm; l) AC-HRTEM image of K-PHI photocatalyst. Scale bar 20 nm.

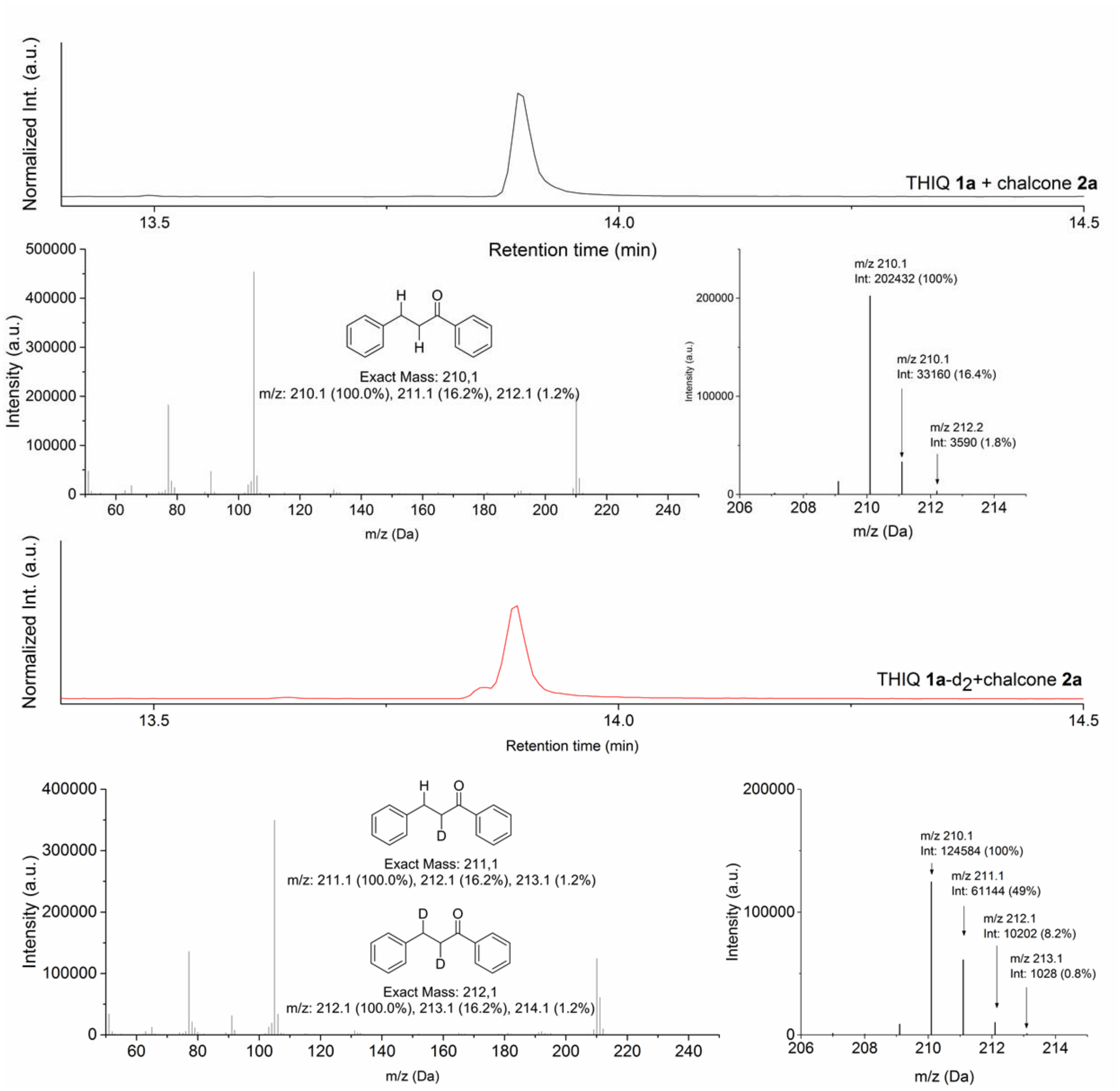

Supplementary Figure 2. Fragments of gas chromatograms of the reaction mixtures. THIQ **1a** and chalcone **2a** (top) and THIQ **1a**-d_2 and chalcone **2a** (bottom).

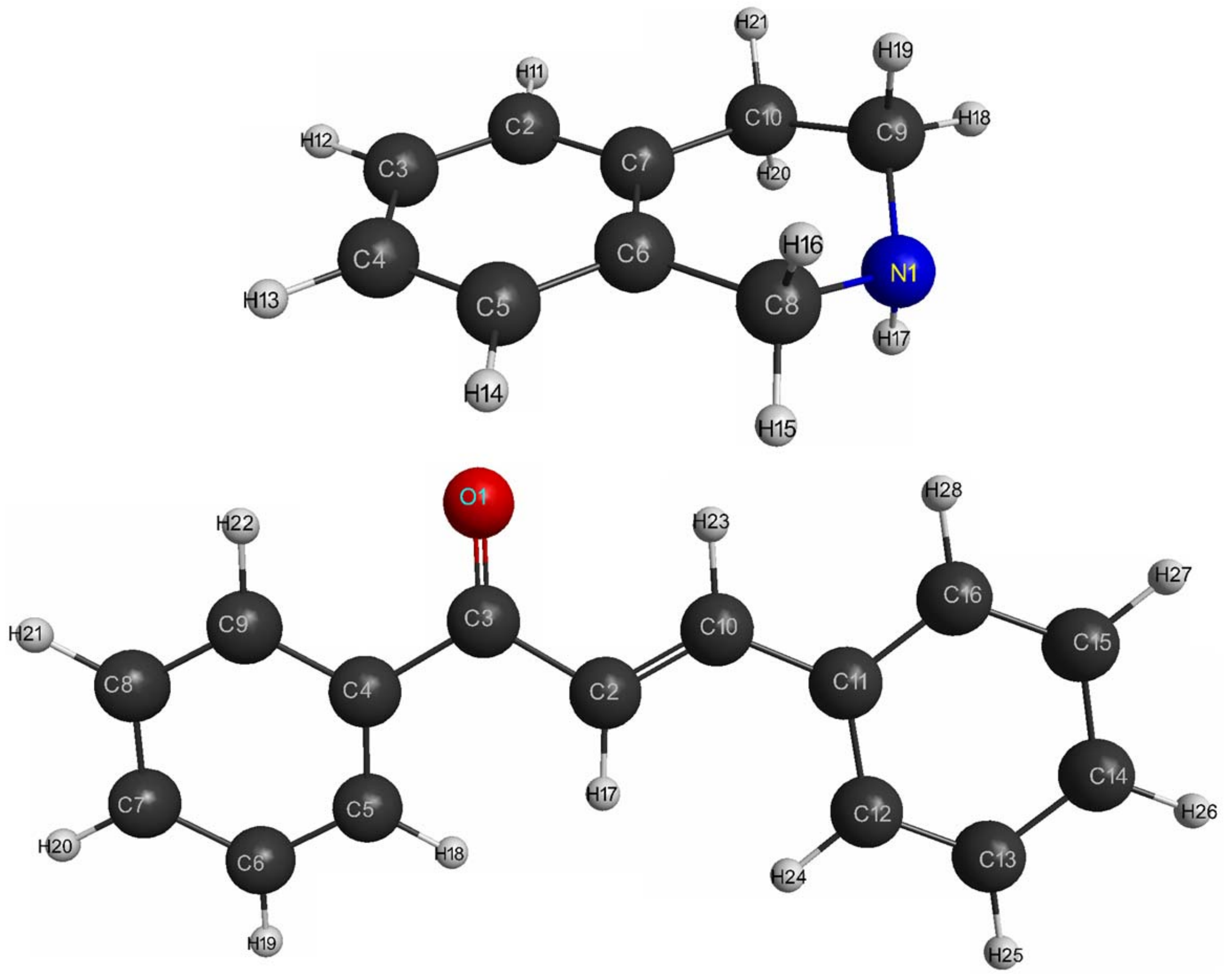

Supplementary Figure 3. Atom numbering scheme for **[1a]** and **[2a]**.

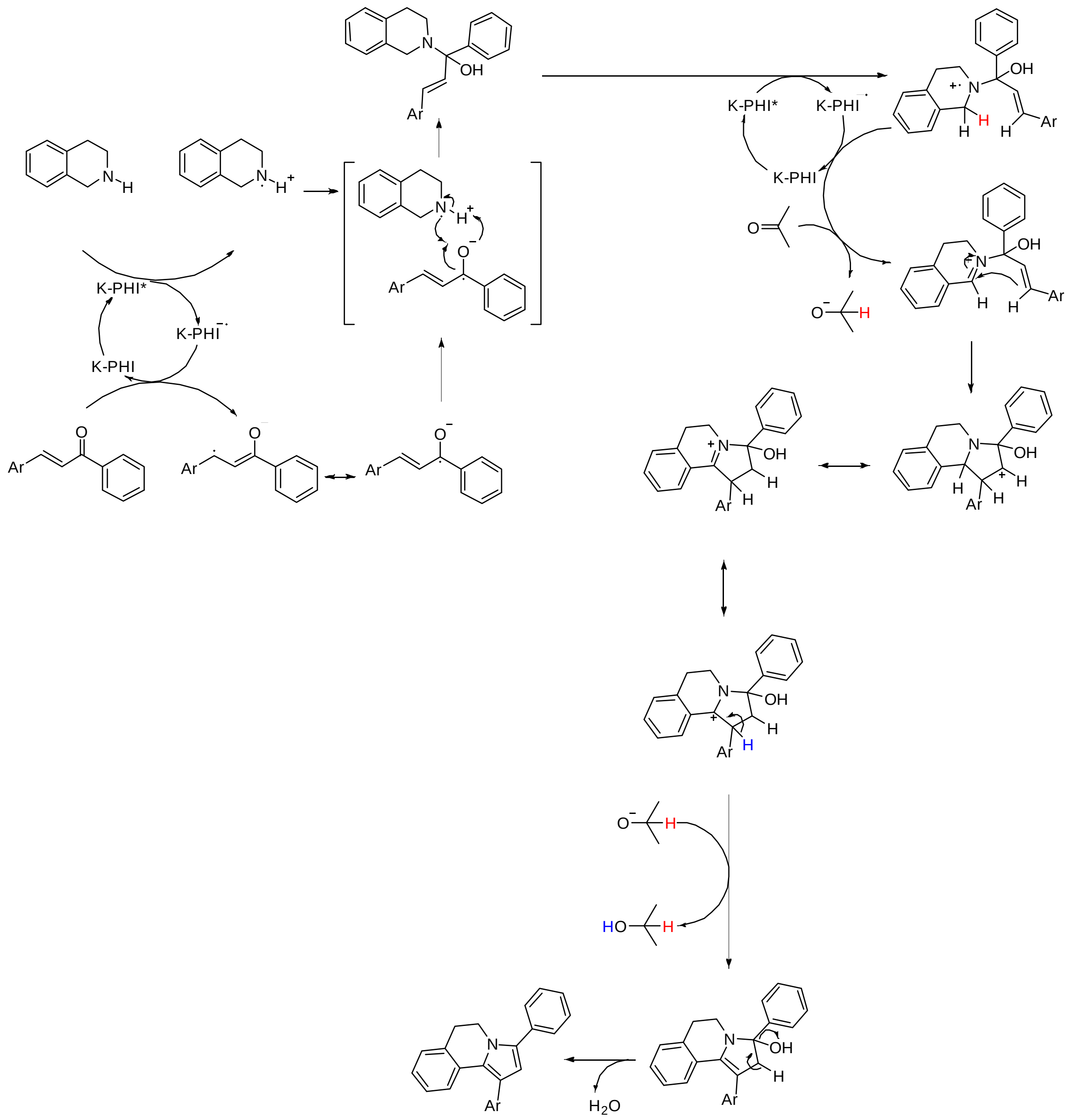

Supplementary Figure 4. Extended scheme of the mechanism.

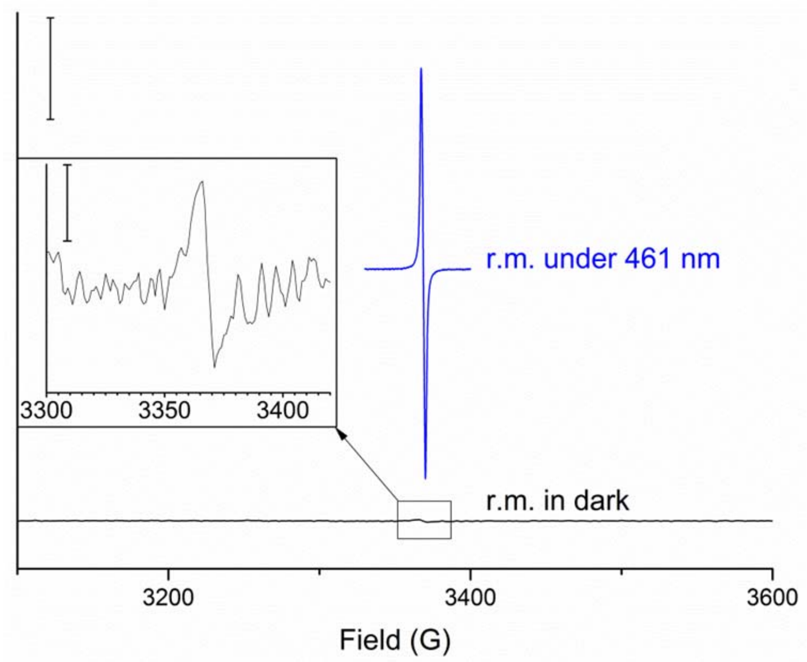

Supplementary Figure 5. *In-situ* EPR spectra of the reaction mixture acquired in dark and under irradiation with blue LED (461 nm, 6 mW·cm^{-2}). Scale bar 10^{-3} a.u. Inset represents a magnified area of the spectrum acquired in dark. Scale bar 10^{-5} a.u.

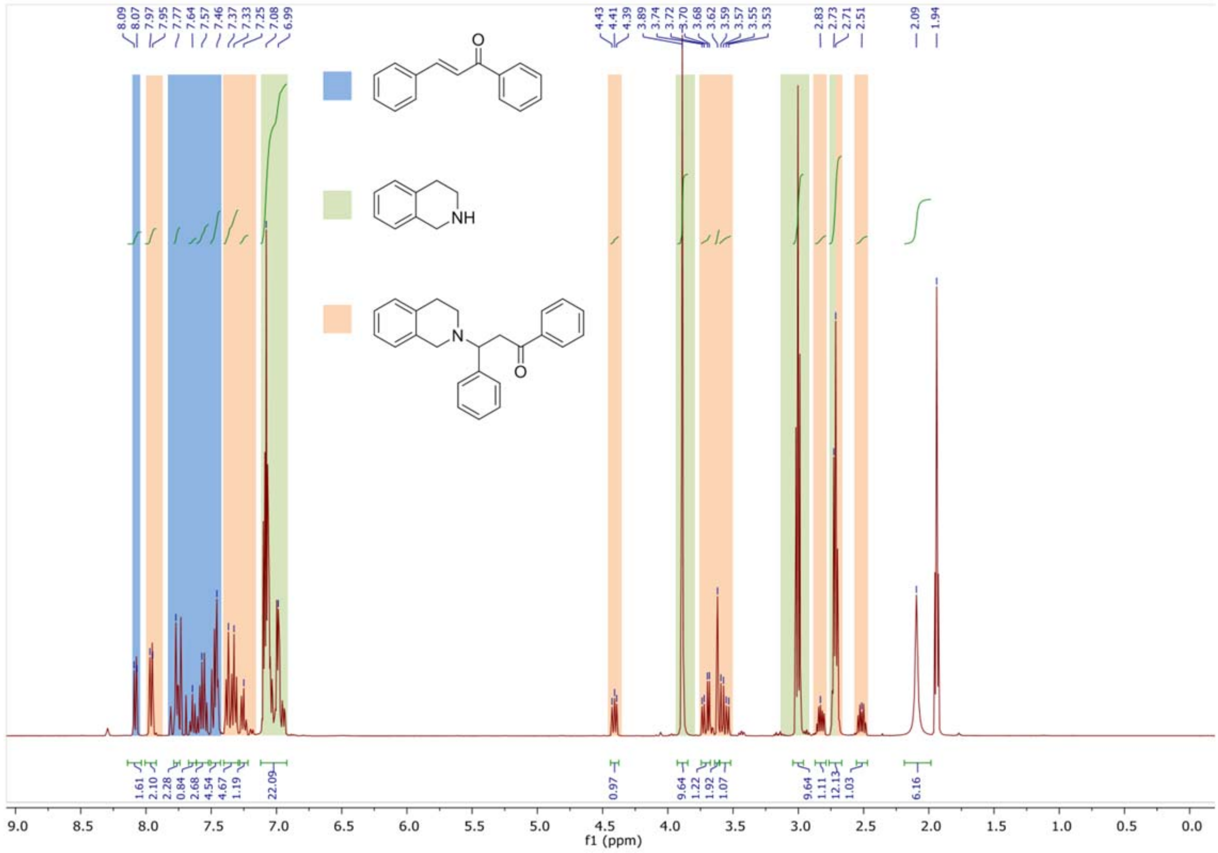

Supplementary Figure 6. NMR spectra of a mixture of chalcone **2a** and THIQ in CD_3CN. The reaction conditions were: Chalcone (50 μmol), THIQ (150 μmol), CD_3CN (0.4 mL), room temperature, 72 h.

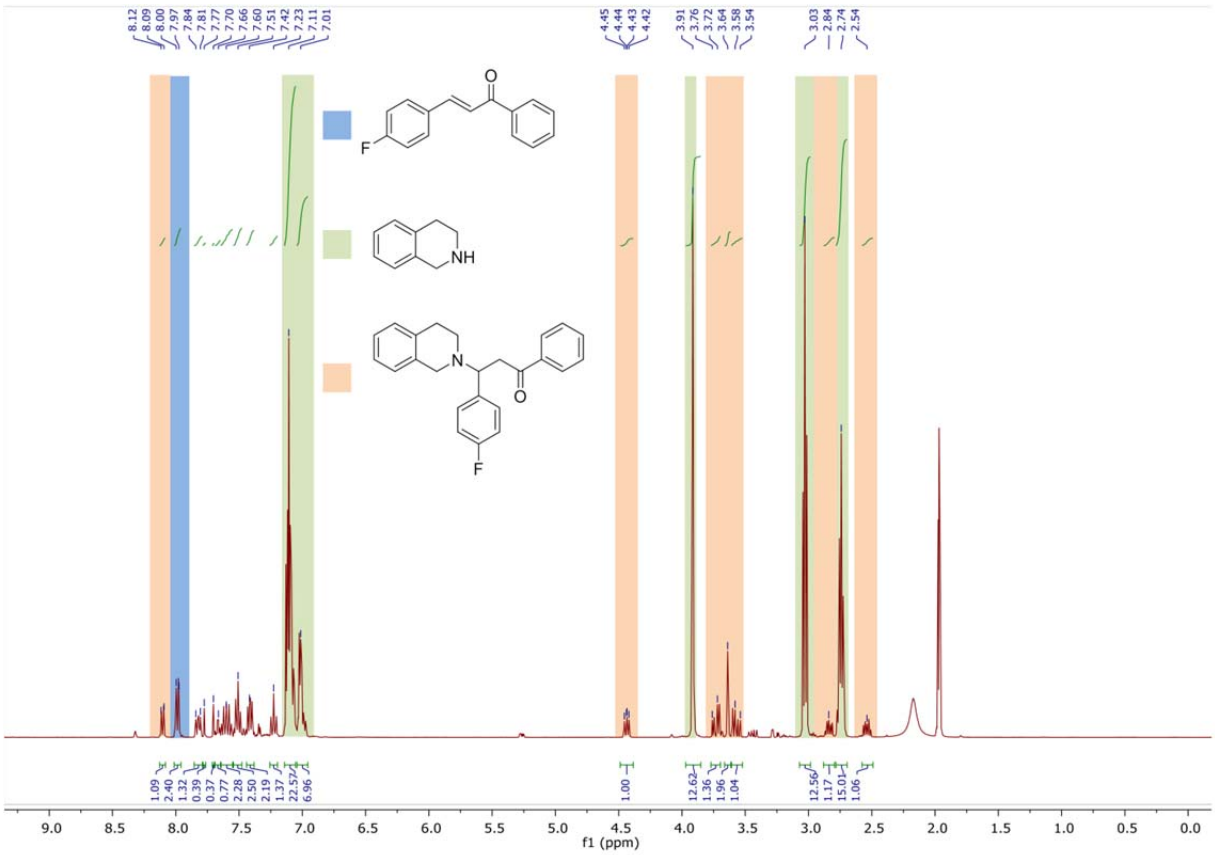

Supplementary Figure 7. NMR spectra of a mixture of chalcone **2d** and THIQ in CD_3CN. The reaction conditions were: Chalcone (50 µmol), THIQ (150 µmol), CD_3CN (0.4 mL), room temperature, 72 h.

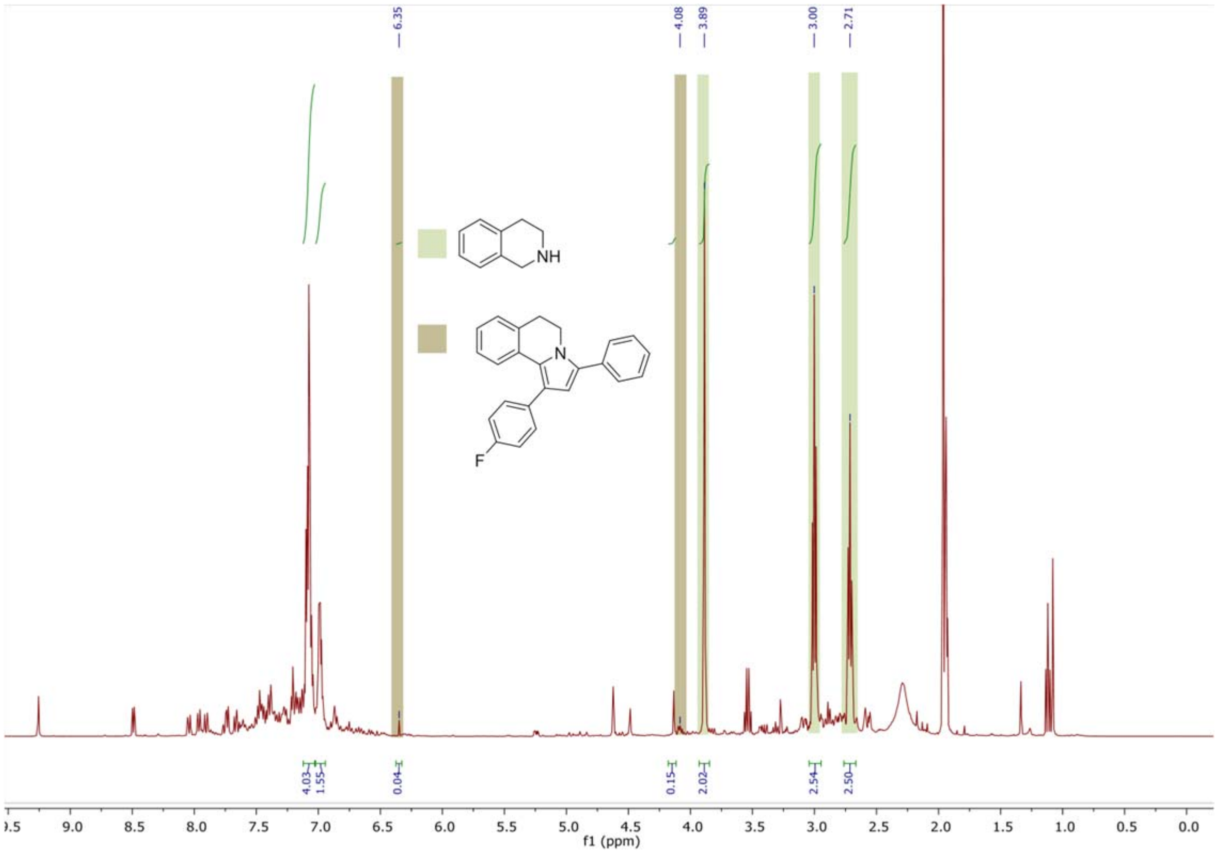

Supplementary Figure 8. NMR spectra of a mixture of chalcone **2d** and THIQ in CD_3CN after addition of K-PHI and light irradiation at 80°C for 20 h.

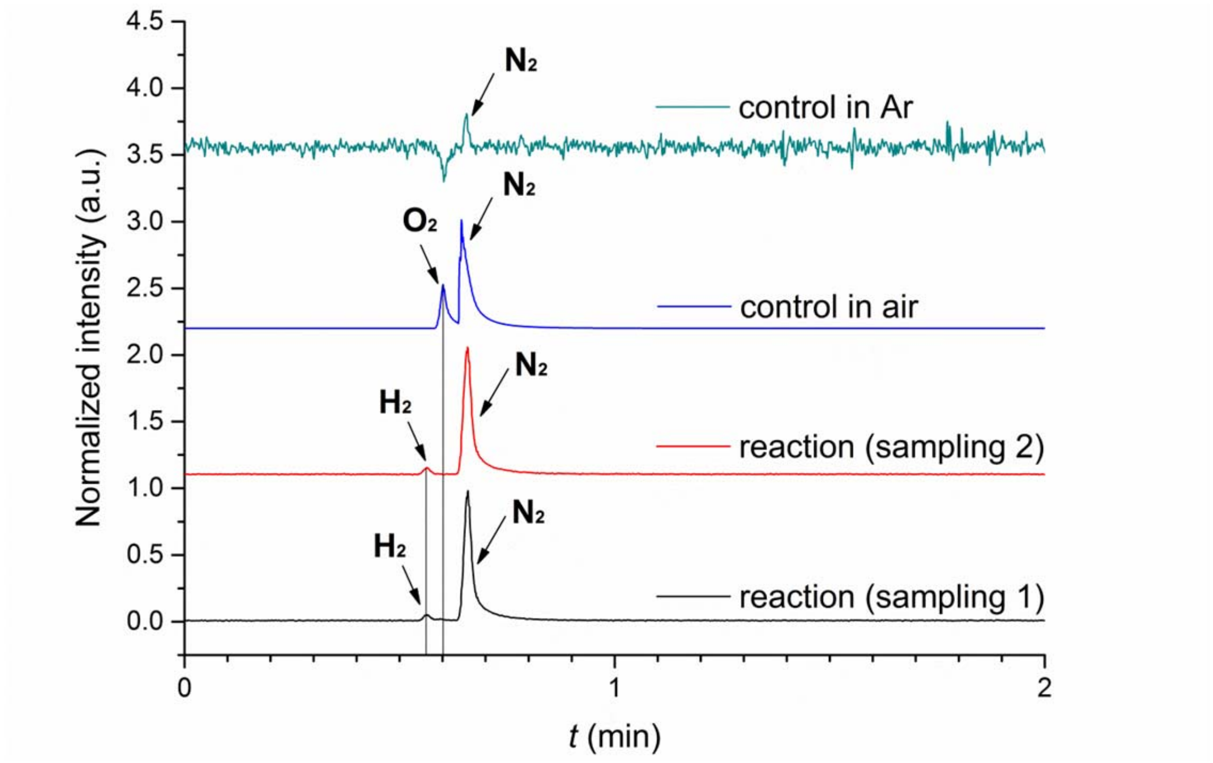

Supplementary Figure 9. GC-TCD of the head space after the photocatalytic experiment and control experiments after injection of air and Ar respectively.

Supplementary Tables

Supplementary Table 1. Atomic spin population calculated for **[1a]**$^{\bullet+}$

Atom		Mulliken population			Lowdin population		
		PBE0-D3	B3LYP-D3	CAMB3LYP	PBE0-D3	B3LYP-D3	CAMB3LYP
1	N	0.61	0.56	0.69	0.55	0.51	0.63
2	C	-0.03	-0.03	-0.02	-0.01	-0.01	-0.01
3	C	0.08	0.09	0.05	0.07	0.08	0.04
4	C	0.05	0.07	0.03	0.05	0.06	0.03
5	C	-0.02	-0.03	-0.01	-0.01	-0.01	0.00
6	C	0.16	0.17	0.13	0.15	0.16	0.12
7	C	0.08	0.10	0.05	0.07	0.09	0.05
8	C	-0.02	-0.02	-0.02	0.01	0.01	0.01
9	C	-0.02	-0.01	-0.03	0.01	0.02	0.01
10	C	0.08	0.07	0.08	0.07	0.07	0.07
11	H	0.00	0.00	0.00	0.00	0.00	0.00
12	H	0.00	0.00	0.00	0.00	0.00	0.00
13	H	0.00	0.00	0.00	0.00	0.00	0.00
14	H	0.00	0.00	0.00	0.00	0.00	0.00
15	H	0.01	0.01	0.01	0.01	0.01	0.01
16	H	0.00	0.00	0.00	0.00	0.00	0.00
17	H	-0.02	-0.02	-0.02	0.00	0.00	0.00
18	H	0.01	0.01	0.01	0.01	0.01	0.01
19	H	0.02	0.02	0.02	0.02	0.01	0.02
20	H	0.01	0.01	0.01	0.01	0.01	0.00
21	H	0.00	0.00	0.00	0.00	0.00	0.00

Supplementary Table 2. Atomic charges calculated for **[1a]** and **[1a]$^{•+}$**

Atom		Mulliken						Lowdin					
		PBE0-D3		B3LYP-D3		CAMB3LYP		PBE0-D3		B3LYP-D3		CAMB3LYP	
		**[1a]**	**[1a]$^{•+}$**	**[1a]**	**[1a]$^{•+}$**	**[1a]**	**[1a]$^{•+}$**	**[1a]**	**[1a]$^{•+}$**	**[1a]**	**[1a]$^{•+}$**	**[1a]**	**[1a]$^{•+}$**
1	N	-0.39	-0.11	-0.39	-0.14	-0.40	-0.09	-0.57	-0.29	-0.58	-0.32	-0.58	-0.26
2	C	-0.25	-0.22	-0.26	-0.22	-0.25	-0.22	-0.13	-0.10	-0.13	-0.10	-0.13	-0.10
3	C	-0.16	-0.12	-0.14	-0.10	-0.15	-0.11	-0.14	-0.06	-0.13	-0.06	-0.13	-0.07
4	C	-0.17	-0.12	-0.15	-0.10	-0.15	-0.11	-0.14	-0.08	-0.13	-0.06	-0.13	-0.08
5	C	-0.25	-0.25	-0.25	-0.25	-0.25	-0.25	-0.13	-0.10	-0.13	-0.10	-0.13	-0.10
6	C	0.23	0.25	0.25	0.28	0.23	0.26	-0.05	-0.01	-0.05	0.00	-0.05	-0.02
7	C	0.25	0.28	0.28	0.31	0.25	0.27	-0.05	-0.01	-0.04	0.00	-0.04	-0.02
8	C	-0.32	-0.35	-0.30	-0.33	-0.30	-0.34	-0.08	-0.04	-0.07	-0.03	-0.07	-0.03
9	C	-0.24	-0.30	-0.20	-0.25	-0.20	-0.27	-0.09	-0.06	-0.08	-0.05	-0.08	-0.05
10	C	-0.40	-0.39	-0.37	-0.36	-0.36	-0.36	-0.21	-0.16	-0.20	-0.16	-0.20	-0.15
11	H	0.13	0.16	0.11	0.15	0.12	0.16	0.13	0.16	0.13	0.15	0.13	0.15
12	H	0.13	0.18	0.11	0.16	0.12	0.16	0.13	0.16	0.13	0.16	0.13	0.16
13	H	0.13	0.18	0.11	0.16	0.12	0.17	0.13	0.16	0.13	0.16	0.13	0.16
14	H	0.12	0.16	0.11	0.15	0.12	0.16	0.14	0.16	0.13	0.15	0.13	0.15
15	H	0.15	0.21	0.13	0.19	0.14	0.20	0.15	0.18	0.14	0.17	0.14	0.18
16	H	0.17	0.23	0.16	0.21	0.16	0.22	0.14	0.17	0.14	0.17	0.14	0.17
17	H	0.25	0.35	0.24	0.32	0.24	0.34	0.22	0.26	0.21	0.26	0.21	0.27
18	H	0.14	0.22	0.12	0.20	0.13	0.20	0.14	0.17	0.14	0.17	0.14	0.17
19	H	0.17	0.23	0.15	0.21	0.15	0.22	0.14	0.17	0.14	0.17	0.14	0.17
20	H	0.16	0.21	0.14	0.20	0.15	0.20	0.13	0.16	0.13	0.16	0.13	0.16
21	H	0.15	0.21	0.13	0.19	0.13	0.20	0.13	0.16	0.13	0.15	0.13	0.16

Supplementary Table 3. Atomic spin population calculated for **[2a]**$^{\bullet-}$

Atom		Mulliken population			Lowdin population		
		PBE0-D3	B3LYP-D3	CAMB3LYP	PBE0-D3	B3LYP-D3	CAMB3LYP
1	O	0.20	0.20	0.20	0.19	0.19	0.20
2	C	0.00	0.02	-0.02	0.03	0.04	0.01
3	C	0.17	0.16	0.18	0.15	0.15	0.16
4	C	-0.02	-0.02	-0.04	-0.01	0.00	-0.02
5	C	0.05	0.04	0.05	0.04	0.04	0.04
6	C	-0.03	-0.02	-0.04	-0.02	-0.01	-0.02
7	C	0.10	0.10	0.09	0.08	0.08	0.07
8	C	-0.05	-0.04	-0.05	-0.03	-0.02	-0.03
9	C	0.09	0.08	0.09	0.07	0.07	0.07
10	C	0.29	0.27	0.34	0.24	0.23	0.28
11	C	-0.03	-0.01	-0.05	-0.01	0.00	-0.03
12	C	0.14	0.12	0.16	0.11	0.10	0.13
13	C	-0.08	-0.06	-0.09	-0.05	-0.04	-0.06
14	C	0.18	0.17	0.20	0.14	0.13	0.16
15	C	-0.06	-0.05	-0.08	-0.04	-0.03	-0.05
16	C	0.10	0.09	0.12	0.09	0.08	0.10
17	H	0.00	0.00	0.00	0.00	0.00	0.00
18	H	0.00	0.00	0.00	0.00	0.00	0.00
19	H	0.00	0.00	0.00	0.00	0.00	0.00
20	H	-0.01	-0.01	-0.01	0.00	0.00	0.00
21	H	0.00	0.00	0.00	0.00	0.00	0.00
22	H	-0.01	-0.01	-0.01	0.00	0.00	0.00
23	H	-0.02	-0.02	-0.02	0.00	0.00	0.00
24	H	-0.01	-0.01	-0.01	0.00	0.00	0.00
25	H	0.00	0.00	0.00	0.00	0.00	0.00
26	H	-0.01	-0.01	-0.01	0.00	0.00	0.00
27	H	0.00	0.00	0.00	0.00	0.00	0.00
28	H	-0.01	-0.01	-0.01	0.00	0.00	0.00

Supplementary Table 4. Atomic charges calculated for **[2a]** and **[2a]$^{\bullet-}$**

Atom		Mulliken						Lowdin					
		PBE0-D3		B3LYP-D3		CAMB3LYP		PBE0-D3		B3LYP-D3		CAMB3LYP	
		**[2a]**	**[2a]$^{\bullet-}$**	**[2a]**	**[2a]$^{\bullet-}$**	**[2a]**	**[2a]$^{\bullet-}$**	**[2a]**	**[2a]$^{\bullet-}$**	**[2a]**	**[2a]$^{\bullet-}$**	**[2a]**	**[2a]$^{\bullet-}$**
1	O	-0.40	-0.59	-0.44	-0.63	-0.44	-0.65	-0.47	-0.63	-0.48	-0.64	-0.48	-0.65
2	C	-0.22	-0.24	-0.20	-0.23	-0.21	-0.24	-0.15	-0.19	-0.15	-0.19	-0.15	-0.20
3	C	0.36	0.33	0.34	0.32	0.34	0.30	0.22	0.13	0.22	0.14	0.23	0.14
4	C	0.28	0.27	0.31	0.30	0.30	0.28	-0.05	-0.03	-0.04	-0.02	-0.04	-0.02
5	C	-0.24	-0.26	-0.24	-0.26	-0.24	-0.26	-0.12	-0.14	-0.11	-0.14	-0.11	-0.14
6	C	-0.16	-0.17	-0.14	-0.15	-0.15	-0.16	-0.13	-0.16	-0.13	-0.16	-0.13	-0.16
7	C	-0.14	-0.19	-0.12	-0.17	-0.12	-0.17	-0.12	-0.19	-0.11	-0.18	-0.11	-0.18
8	C	-0.17	-0.17	-0.14	-0.15	-0.15	-0.15	-0.13	-0.15	-0.12	-0.15	-0.13	-0.15
9	C	-0.23	-0.26	-0.22	-0.26	-0.23	-0.26	-0.08	-0.12	-0.07	-0.11	-0.07	-0.11
10	C	-0.13	-0.25	-0.14	-0.24	-0.14	-0.26	-0.04	-0.16	-0.04	-0.15	-0.03	-0.16
11	C	0.42	0.42	0.44	0.45	0.42	0.44	-0.04	-0.04	-0.03	-0.03	-0.04	-0.03
12	C	-0.25	-0.30	-0.25	-0.30	-0.25	-0.30	-0.11	-0.16	-0.11	-0.16	-0.11	-0.16
13	C	-0.15	-0.16	-0.13	-0.13	-0.14	-0.14	-0.13	-0.15	-0.12	-0.15	-0.13	-0.15
14	C	-0.15	-0.22	-0.13	-0.20	-0.13	-0.21	-0.12	-0.21	-0.12	-0.20	-0.12	-0.21
15	C	-0.15	-0.16	-0.13	-0.13	-0.14	-0.13	-0.13	-0.16	-0.12	-0.16	-0.13	-0.16
16	C	-0.26	-0.30	-0.26	-0.30	-0.27	-0.31	-0.12	-0.16	-0.11	-0.16	-0.12	-0.16
17	H	0.11	0.07	0.10	0.07	0.11	0.07	0.13	0.12	0.13	0.11	0.13	0.11
18	H	0.11	0.09	0.10	0.08	0.11	0.09	0.14	0.13	0.13	0.12	0.13	0.13
19	H	0.13	0.09	0.11	0.08	0.12	0.09	0.14	0.12	0.13	0.11	0.13	0.12
20	H	0.13	0.10	0.12	0.08	0.13	0.09	0.14	0.12	0.13	0.11	0.14	0.12
21	H	0.13	0.09	0.12	0.08	0.12	0.09	0.14	0.12	0.13	0.11	0.14	0.12
22	H	0.17	0.17	0.16	0.16	0.17	0.17	0.17	0.16	0.16	0.16	0.17	0.16
23	H	0.17	0.15	0.16	0.14	0.17	0.15	0.17	0.15	0.16	0.14	0.17	0.15
24	H	0.12	0.10	0.11	0.09	0.11	0.10	0.14	0.13	0.13	0.12	0.13	0.12
25	H	0.13	0.09	0.11	0.07	0.12	0.08	0.14	0.12	0.13	0.11	0.14	0.12
26	H	0.14	0.10	0.12	0.08	0.13	0.09	0.14	0.12	0.13	0.11	0.13	0.11
27	H	0.13	0.09	0.12	0.07	0.12	0.08	0.14	0.12	0.13	0.11	0.14	0.12
28	H	0.14	0.11	0.12	0.09	0.13	0.10	0.14	0.13	0.14	0.12	0.14	0.12

Supplementary Notes

Supplementary Note 1

NMR tube was charged with THIQ (150 μmol), chalcone **2a** (50 μmol) and acetonitrile-d_3 (0.4 mL). The resultant solution was allowed to react at room temperature (+25°C) for 72 h. ^{1}H NMR spectrum was acquired. Aza-Michael adduct (~57%) was detected (Supplementary Figure 3).

Supplementary Note 2

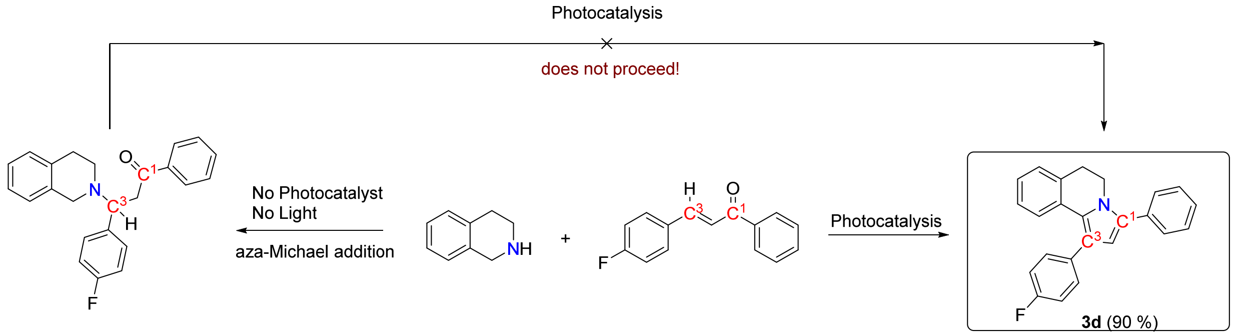

NMR tube was charged with THIQ (150 μmol), chalcone **2d** (50 μmol) and acetonitrile-d_3 (0.4 mL). The resultant solution was allowed to react at room temperature (+25°C) for 24 h. ^{1}H NMR spectrum was acquired. Aza-Michael adduct (~31%) was detected (Supplementary Figure 4).

Supplementary Note 3

The solution from NMR tube prepared according to the Supplementary Note 2 was transferred into a glass tube with rubber-lined cap. Acetonitrile (1.6 mL), acetone (9mg, 150 μmol), K-PHI (5 mg) were added. The mixture was purged with Ar for 1 min and stirred at 80°C under irradiation of blue LED (λ=461nm, 51.7±0.03 mW·cm^{-2}) for 20 hours. Then the reaction mixture was cooled to room temperature and centrifuged, clear solution was separated and solid residue was washed with acetonitrile (2 mL) and centrifuged again. Organic solutions were combined and evaporated to dryness. The residue after evaporation was analyzed by ^{1}H NMR in CD_3CN. Traces of DHPIQ were observed (Supplementary Figure 5).

Supplementary Note 4

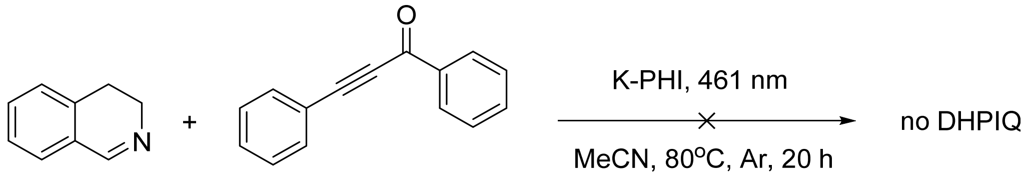

A glass tube with rubber-lined cap was evacuated and filled with argon three times. To this tube 3,4-dihydroisoquinoline (150 μmol), 1,3-diphenylprop-2-yn-1-one (50 μmol), acetone (9mg, 150 μmol), K-PHI (5 mg) and acetonitrile (2 mL) were added. The resulting mixture was stirred at 80°C under irradiation of blue LED (λ=461nm, 51.7±0.03 mW·cm^{-2}) for 20 hours. Then the reaction mixture was cooled to room temperature and centrifuged, clear solution was separated and solid residue was washed with acetonitrile (2 mL) and centrifuged again. Organic solutions

were combined and evaporated to dryness. The residue after evaporation was analyzed by ^{1}H NMR in CD_3CN. No DHPIQ was observed.

Supplementary Note 5

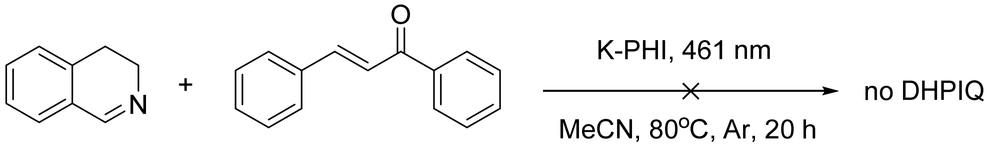

A glass tube with rubber-lined cap was evacuated and filled with argon three times. To this tube 3,4-dihydroisoquinoline (150 μmol), chalcone **2a** (50 μmol), acetone (9mg, 150 μmol), K-PHI (5 mg) and acetonitrile (2 mL) were added. The resulting mixture was stirred at 80°C under irradiation of blue LED (λ=461nm, 51.7±0.03 mW·cm^{-2}) for 20 hours. Then the reaction mixture was cooled to room temperature and centrifuged, clear solution was separated and solid residue was washed with acetonitrile (2 mL) and centrifuged again. Organic solutions were combined and evaporated to dryness. The residue after evaporation was analyzed by ^{1}H NMR in CD_3CN. No DHPIQ was observed.

Supplementary Note 6

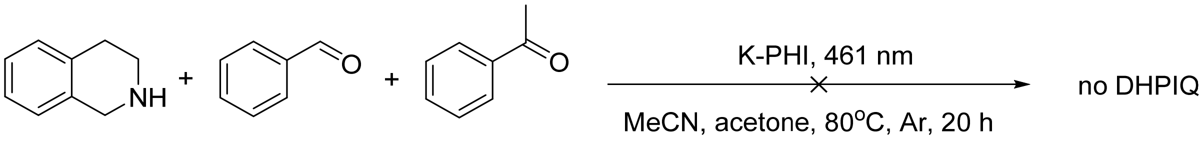

A glass tube with rubber-lined cap was evacuated and filled with argon three times. To this tube THIQ (150 μmol), acetophenone (50 μmol), benzaldehyde (50 μmol), acetone (9mg, 150 μmol), K-PHI (5 mg) and acetonitrile (2 mL) were added. The resulting mixture was stirred at 80°C under irradiation of blue LED (λ=461nm, 51.7±0.03 mW·cm^{-2}) for 20 hours. Then the reaction mixture was cooled to room temperature and centrifuged, clear solution was separated and solid residue was washed with acetonitrile (2 mL) and centrifuged again. Organic solutions were combined and evaporated to dryness. The residue after evaporation was analyzed by ^{1}H NMR in CD_3CN. No DHPIQ was observed.

Supplementary Note 7

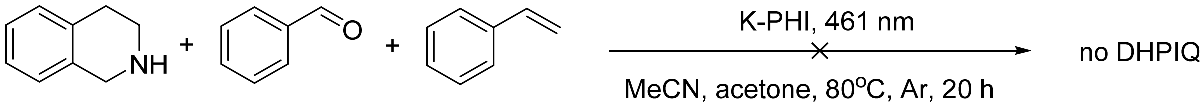

A glass tube with rubber-lined cap was evacuated and filled with argon three times. To this tube THIQ (150 μmol), styrene (50 μmol), benzaldehyde (50 μmol), acetone (9mg, 150 μmol), K-PHI (5 mg) and acetonitrile (2 mL) were added. The resulting mixture was stirred at 80°C under irradiation of blue LED (λ=461nm, 51.7±0.03 mW·cm^{-2}) for 20 hours. Then the reaction mixture was cooled to room temperature and centrifuged, clear solution was separated and solid residue

was washed with acetonitrile (2 mL) and centrifuged again. Organic solutions were combined and evaporated to dryness. The residue after evaporation was analyzed by ^{1}H NMR in CD_3CN. No DHPIQ was observed.

Supplementary Note 8

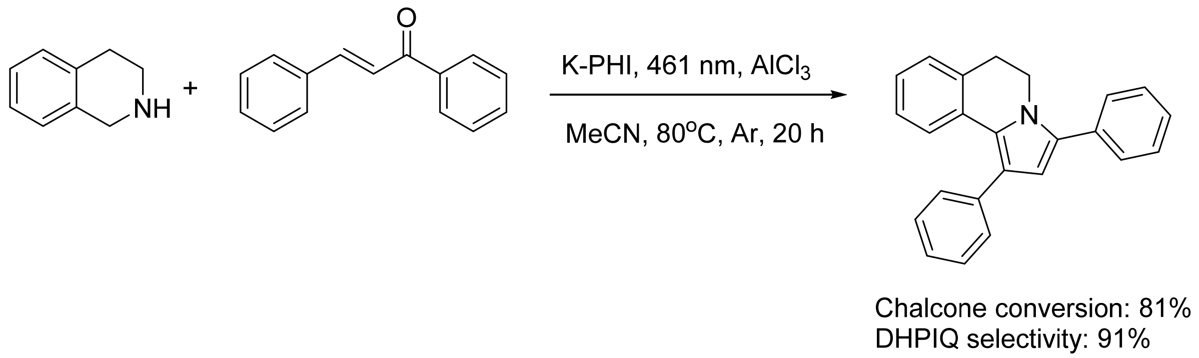

A glass tube with rubber-lined cap was evacuated and filled with argon three times. To this tube THIQ (150 µmol), chalcone (50 µmol), $AlCl_3$ (50 µmol), acetone (9mg, 150 µmol), K-PHI (5 mg) and acetonitrile (2 mL) were added. The resulting mixture was stirred at 80°C under irradiation of blue LED (λ=461nm, 51.7±0.03 mW·cm^{-2}) for 20 hours. Then the reaction mixture was cooled to room temperature and centrifuged, clear solution was separated and solid residue was washed with acetonitrile (2 mL) and centrifuged again. Organic solutions were combined and evaporated to dryness. The residue after evaporation was analyzed by ^{1}H NMR in CD_3CN. Chalcone conversion: 81%, DHPIQ yield: 91%.

Supplementary Methods

General method of chalcones (2a-k,p,q) preparation

To a mixture of acetophenone (1 g, 8.3 mmol) and corresponding aldehyde (9.1 mmol) in 50 mL of mixture methanol/water (1:4), solution of sodium hydroxide (667 mg, 16.6 mmol) in 2 mL of water was added dropwise at room temperature (in case of chalcone **2p** 1.334 g (33.2 mmol) of sodium hydroxide was used) and resulted solution was stirred for 20 hours at room temperature, in case of chalcones **2c,i -** for 20 hours at reflux. Then reaction mixture was cooled to room temperature and for chalcones **2a-f,h-j** 50 mL of water was added, obtained precipitate was filtered, washed with water (3 x 15 mL) then with 10 mL of mixture water/ethanol (4:1) and dried on air to give product as solid material. In case of chalcones **2g,q** precipitate after filtration was redisolved in 30 mL of mixture ethanol and aq. (2N) hydrochloride acid (1:1) and refluxed for 2 hours, then cooled to room temperature, dilluted with 50 mL of water and obtained precipitate was filtered, washed with water (3 x 15 mL) then with 10 mL of mixture water/ethanol (4:1) then dried on air to give product as solid material. For chalcone **2k** reaction mixture after cooling was dilluted with 50 mL of water and acidified with aq. (2N) hydrochloride acid to pH=5, then extracted with chloroform (3 x 15 mL), all organic solutions were combined, washed with brine (2 x 10 mL), dried over Na_2SO_4, evaporated to give product as orange oil. In case of chalcone **2p**, precipitate which was formed after cooling was separated by filtration, redissolved in 50 mL of water, obtained solution was acidified with aq. (2N) hydrochloride acid to pH=5 and obtained precipitate was filtered, washed with water (3 x 20 mL) then with 10 mL of mixture water/ethanol (4:1) and dried on air to give product as solid material.

Preparation of chalkone 2l

To stirring mixture of benzaldehyde (12 g, 0.11 mol) and acetone (40 g, 0.68 mol) in water (20 mL), aqueous (20%) solution of sodium hydroxide (1.5 mL) was added dropwise at 25°C. After addition reaction mixture was stirred for another 2 hours at 25°C. Then mixture was dilluted with water (100 mL) and acidified with aq. (2N) hydrochloride acid to pH=5, then extracted with chloroform (3 x 15 mL), all organic solutions were combined, washed with brine (2 x 10 mL), dried over Na_2SO_4. Residue after evaporation was redisolved in 50 mL of mixture ethanol and aq. (2N) hydrochloride acid (1:1) and refluxed for 2 hours. Then cooled to room temperature, dilluted with 50 mL of water, extracted with chloroform (3 x 15 mL), all organic solutions were combined, washed with brine (2 x 10 mL), dried over Na_2SO_4, evaporated to give as residue orange oil, vacuum distilation of which gives pure chalcone **2l** (yield 74%).

(E)-chalcone[1] (2a).

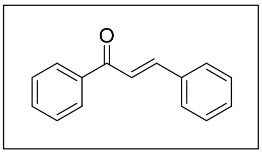

Yellowish solid (yield 73%). ^{1}H NMR (400 MHz, $CDCl_3$) δ 8.03 (dd, *J* = 8.3, 1.3 Hz, 2H), 7.82 (d, *J* = 15.7 Hz, 1H), 7.65 (dd, *J* = 6.8, 2.8 Hz, 2H), 7.60 (t, *J* = 7.3 Hz, 1H), 7.57 – 7.49 (m, 3H), 7.45 – 7.40 (m, 3H). ^{13}C NMR (101 MHz, $CDCl_3$) δ 190.5, 144.8, 138.2, 134.9, 132.8, 130.5, 128.9, 128.6, 128.5, 128.4, 122.1.

(E)-1-phenyl-3-(p-tolyl)prop-2-en-1-one[2] (2b).

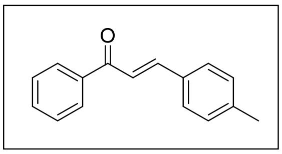

Yellowish solid (yield 82%). ^{1}H NMR (400 MHz, Chloroform-*d*) δ 8.05 – 8.00 (m, 2H), 7.81 (d, *J* = 15.7 Hz, 1H), 7.62 – 7.47 (m, 6H), 7.23 (d, *J* = 8.0 Hz, 2H), 2.40 (s, 3H). ^{13}C NMR (101 MHz, $CDCl_3$) δ 190.7, 145.0, 141.1, 138.3, 132.7, 132.1, 129.7, 128.6, 128.5, 128.5, 121.1, 21.6.

(E)-3-(4-methoxyphenyl)-1-phenylprop-2-en-1-one[1] (2c).

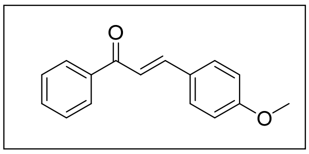

Yellowish solid (yield 82%). ^{1}H NMR (400 MHz, $CDCl_3$) δ 8.01 (dd, *J* = 8.3, 1.3 Hz, 2H), 7.79 (d, *J* = 15.6 Hz, 1H), 7.63 – 7.55 (m, 3H), 7.49 (t, *J* = 7.4 Hz, 2H), 7.42 (d, *J* = 15.6 Hz, 1H), 6.93 (d, *J* = 8.8 Hz, 2H), 3.85 (s, 3H). ^{13}C NMR (101 MHz, $CDCl_3$) δ 190.6, 161.7, 144.7, 138.5, 132.6, 130.28, 128.6, 128.4, 127.6, 119.7, 114.4, 55.5.

(E)-3-(4-fluorophenyl)-1-phenylprop-2-en-1-one[3] (2d).

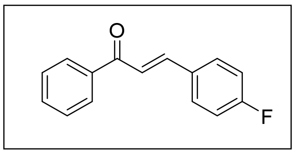

White solid (yield 85%). ^{1}H NMR (400 MHz, $CDCl_3$) δ 8.03 – 8.01 (m, 2H), 7.78 (d, *J* = 15.7 Hz, 1H), 7.69 – 7.55 (m, 3H), 7.54 – 7.41 (m, 3H), 7.12 (t, *J* = 8.6 Hz, 2H). ^{13}C NMR (101 MHz, $CDCl_3$) δ 190.35, 164.07 (d, J = 251.9 Hz), 143.56, 138.11, 132.90, 131.13 (d, J = 3.4 Hz), 130.38 (d, J = 8.5 Hz), 128.69, 128.50, 121.74 (d, J = 2.3 Hz), 116.17 (d, J = 21.9 Hz).

(E)-3-(3-fluorophenyl)-1-phenylprop-2-en-1-one (2e).

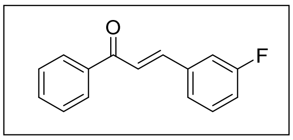

White solid (yield 80%). ^{1}H NMR (400 MHz, Chloroform-*d*) δ 8.06 – 8.00 (m, 2H), 7.76 (d, *J* = 15.7 Hz, 1H), 7.64 – 7.57 (m, 1H), 7.57 – 7.47 (m, 3H), 7.44 – 7.32 (m, 3H), 7.12 (t, *J* = 8.9 Hz, 1H). ^{13}C NMR (101 MHz, Chloroform-*d*) δ 190.19, 163.05 (d, *J* = 246.9 Hz), 143.32 (d, *J* = 2.7 Hz), 137.92, 137.13 (d, *J* = 7.6 Hz), 133.05, 130.54 (d, *J* = 8.3 Hz), 128.73, 128.55, 124.60 (d, *J* = 2.8 Hz), 123.16, 117.41 (d, *J* = 21.5 Hz), 114.49 (d, *J* = 21.9 Hz).

(E)-1-phenyl-3-(4-(trifluoromethyl)phenyl)prop-2-en-1-one[4] (2f).

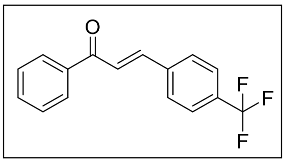

White solid (yield 81%). ^{1}H NMR (400 MHz, $CDCl_3$) δ 8.04 (d, *J* = 7.5 Hz, 2H), 7.81 (d, *J* = 15.7 Hz, 1H), 7.77 – 7.65 (m, 4H), 7.62 – 7.58 (m, 2H), 7.54 – 7.51 (m, 2H). ^{13}C NMR (101 MHz, $CDCl_3$) δ 190.1, 142.8, 138.3, 137.8, 133.2, 131.9 (q, *J* = 32.3 Hz), 128.8, 128.6, 128.5, 125.9 (q, *J* = 4 Hz), 124.2, 123.8(q, *J* = 272 Hz).

(E)-1-phenyl-3-(3-(trifluoromethyl)phenyl)prop-2-en-1-one[5] (2g).

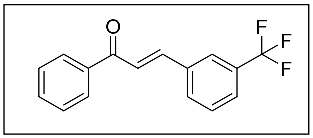

White solid (yield 78%). ^{1}H NMR (400 MHz, Chloroform-*d*) δ 8.07 – 8.02 (m, 2H), 7.89 (s, 1H), 7.85 – 7.79 (m, 2H), 7.69 – 7.50 (m, 6H). ^{13}C NMR (101 MHz, $CDCl_3$) δ 190.05, 142.87, 137.79, 135.66, 133.16, 131.68, 131.50 (q, *J* = 32.3 Hz), 129.56, 128.77, 128.59, 126.87 (q, *J* = 4 Hz), 124.70 (q, *J* = 4 Hz), 123.82 (q, *J* = 273.7 Hz), 123.61.

(E)-3-(3,4-difluorophenyl)-1-phenylprop-2-en-1-one (2h).

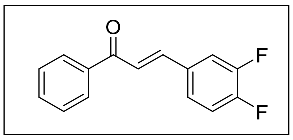

White solid (yield 78%). ^{1}H NMR (400 MHz, Chloroform-*d*) δ 8.04 – 7.99 (m, 2H), 7.71 (d, *J* = 15.7 Hz, 1H), 7.63 – 7.58 (m, 1H), 7.55 – 7.42 (m, 4H), 7.40 – 7.34 (m, 1H), 7.21 (dt, *J* = 9.7, 8.3 Hz, 1H). ^{13}C NMR (101 MHz, Chloroform-*d*) δ 189.94, 151.64 (dd, *J* = 254,5 13.1 Hz), 150.66 (dd, *J* = 250.5, 13.1 Hz), 142.36 (t, *J* = 2.0 Hz), 137.84, 133.11, 132.13 (dd, *J* = 5.9, 4.0 Hz), 128.75, 128.53, 125.36 (dd, *J* = 6.6, 3.4 Hz), 122.83 (d, *J* = 2.4 Hz), 117.94 (d, *J* = 17.7 Hz), 116.51 (d, *J* = 17.6 Hz).

(E)-1-phenyl-3-(thiophen-2-yl)prop-2-en-1-one[6] (2i).

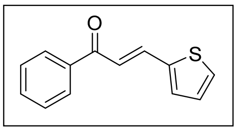

Orange solid (yield 68%). ^{1}H NMR (400 MHz, $CDCl_3$) δ 8.01 (dd, *J* = 8.3, 1.3 Hz, 2H), 7.95 (d, *J* = 15.3 Hz, 1H), 7.61 – 7.55 (m, 1H), 7.53 – 7.47 (m, 2H), 7.43 (d, *J* = 5.0 Hz, 1H), 7.37 (d, *J* = 5.0 Hz, 2H), 7.10 (dd, *J* = 5.0, 3.6 Hz, 1H). ^{13}C NMR (101 MHz, $CDCl_3$) δ 189.9, 140.4, 138.1, 137.3, 132.8, 132.2, 128.9, 128.7, 128.43, 128.41, 120.7.

(E)-1-phenyl-3-(1H-pyrrol-2-yl)prop-2-en-1-one[7] (2j).

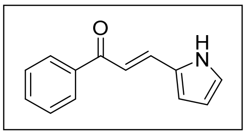

Yellow solid (yield 67%). ^{1}H NMR (400 MHz, DMSO-d_6) δ 11.74 (br s, 1H), 8.10 – 7.97 (m, 2H), 7.68 – 7.51 (m, 5H), 7.21 – 7.09 (m, 1H), 6.75– 6.74 (m, 1H), 6.30 – 6.16 (m, 1H). ^{13}C NMR (101 MHz, DMSO-d_6) δ 188.8, 138.8, 134.8, 133.0, 129.6, 129.2, 128.4, 124.8, 117.0, 115.0, 111.1.

(E)-1-phenyl-3-(thiophen-2-yl)prop-2-en-1-one[6] (2k).

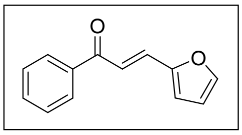

Orange oil (yield 62%). ^{1}H NMR (400 MHz, $CDCl_3$) δ 8.06 – 8.00 (m, 2H), 7.64 – 7.43 (m, 6H), 6.72 (d, *J* = 3.4 Hz, 1H), 6.52 (dd, *J* = 3.4, 1.8 Hz, 1H). ^{13}C NMR (101 MHz, $CDCl_3$) δ 189.9, 151.7, 145.0, 138.2, 132.9, 130.8, 128.7, 128.5, 119.3, 116.4, 112.8.

(E)-4-phenylbut-3-en-2-one[8] (2l)

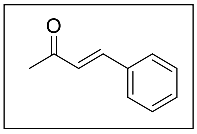

Yellow solid (yield 74%). ^{1}H NMR (400 MHz, Chloroform-*d*) δ 7.57 – 7.49 (m, 3H), 7.43 – 7.38 (m, 3H), 6.72 (d, *J* = 16.3 Hz, 1H), 2.39 (s, 3H). ^{13}C NMR (101 MHz, $CDCl_3$) δ 198.54, 143.52, 134.39, 130.57, 129.00, 128.28, 127.14, 27.56.

(E)-3-(2-hydroxy-3-methoxyphenyl)-1-phenylprop-2-en-1-one[9] (2p).

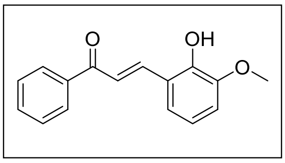

Yellow solid (yield 88%). ^{1}H NMR (400 MHz, $CDCl_3$) δ 8.09 – 7.97 (m, 3H), 7.75 (d, *J* = 15.9 Hz, 1H), 7.59 – 7.48 (m,3H), 7.20 (d, *J* = 7.6 Hz, 1H), 6.90 – 6.87 (m, 2H), 6.26 (s, 1H), 3.94 (s, 3H). ^{13}C

NMR (101 MHz, $CDCl_3$) δ 191.2, 146.8, 145.8, 140.1, 138.5, 132.6, 128.6, 128.6, 123.6, 121.8, 121.3, 119.7, 111.9, 56.3.

(E)-3-(perfluorophenyl)-1-phenylprop-2-en-1-one[10] (2q).

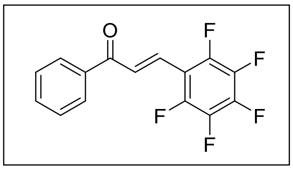

White solid (yield 50%). ^{1}H NMR (400 MHz, Chloroform-*d*) δ 8.05 – 8.00 (m, 2H), 7.90 – 7.74 (m, 2H), 7.66 – 7.60 (m, 1H), 7.53 (t, *J* = 7.6 Hz, 2H). ^{13}C NMR (101 MHz, Chloroform-*d*) δ 189.37, 145.90 (dm, *J* = 254.7 Hz), 141.7 (dm, *J* = 258.6 Hz), 137.9 (dm, *J* = 254.5 Hz), 137.25, 133.54, 128.91 (m), 128.87, 128.66, 127.84 (m), 110.46 (m).

^{1}H and ^{13}C NMR spectra of chalcones (2a-m).

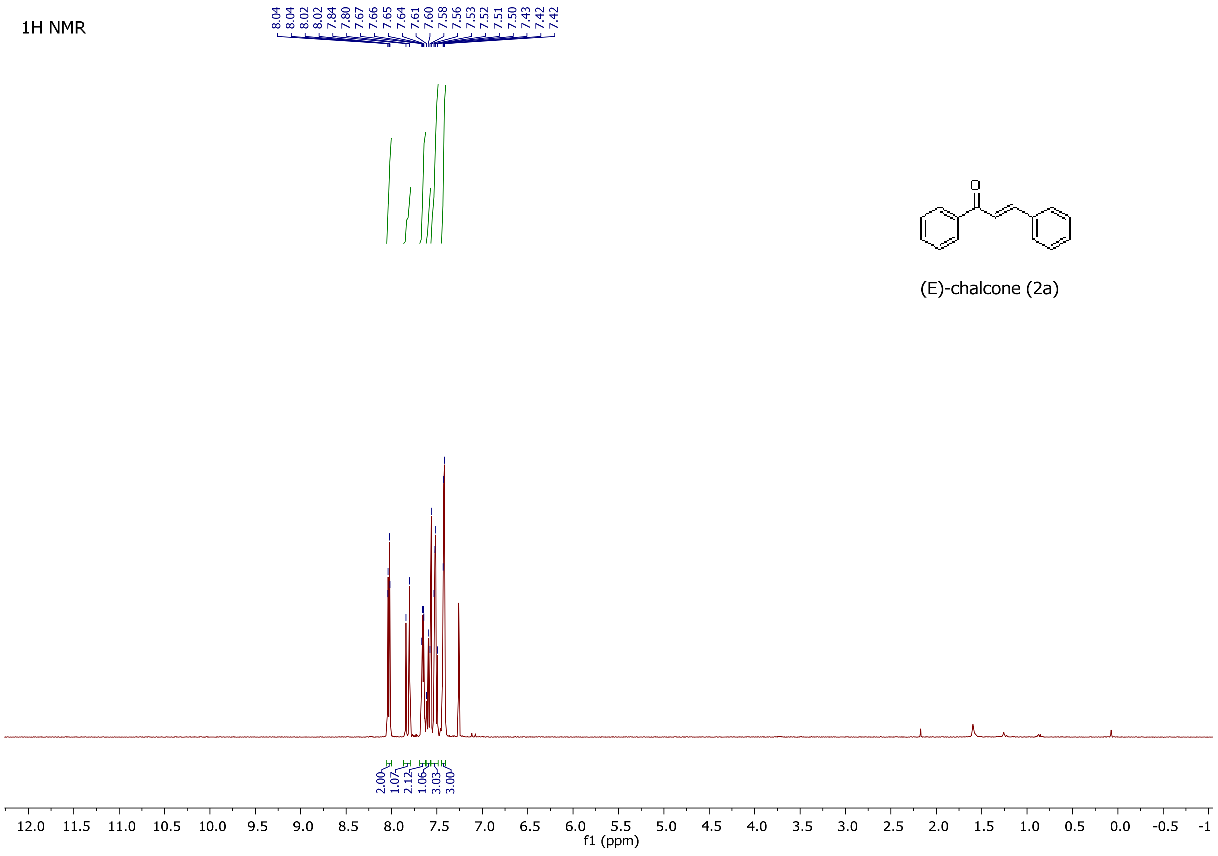

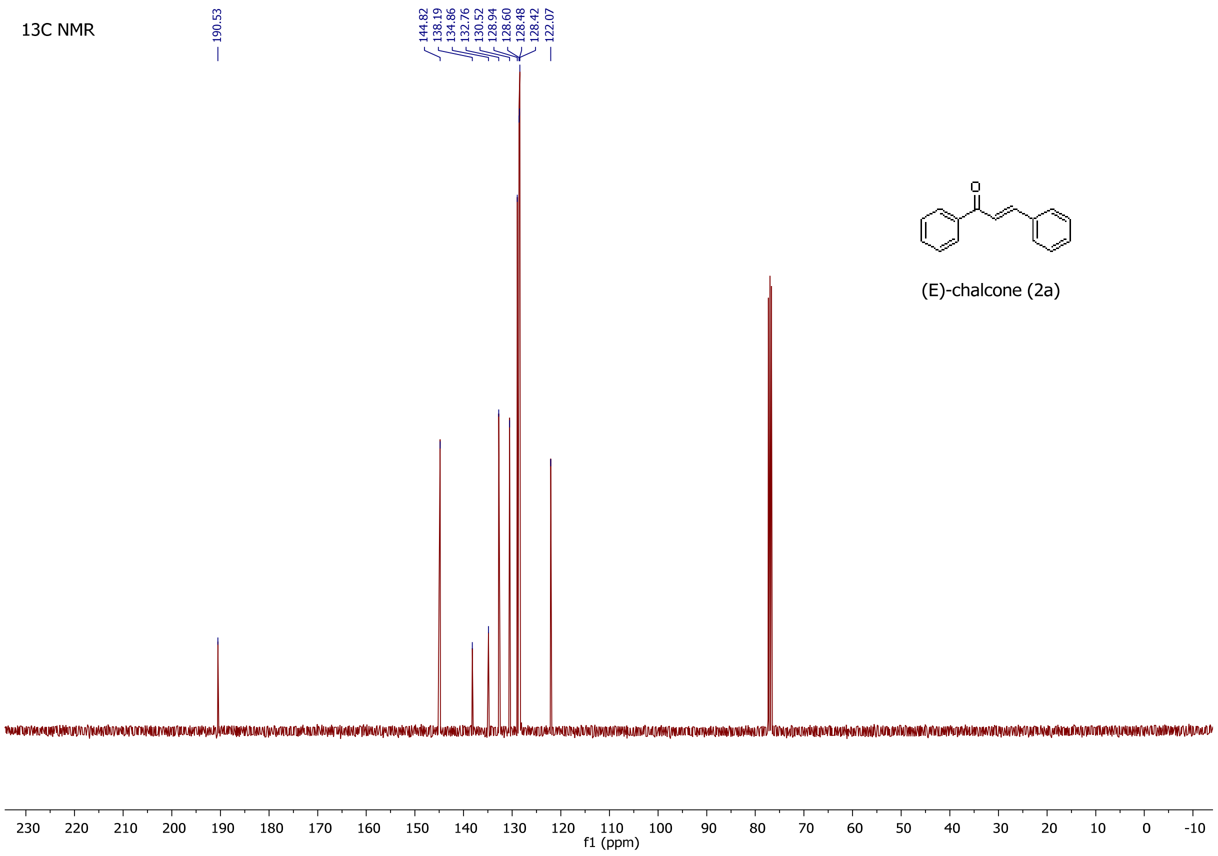

1H NMR

(E)-1-phenyl-3-(p-tolyl)prop-2-en-1-one (2b)

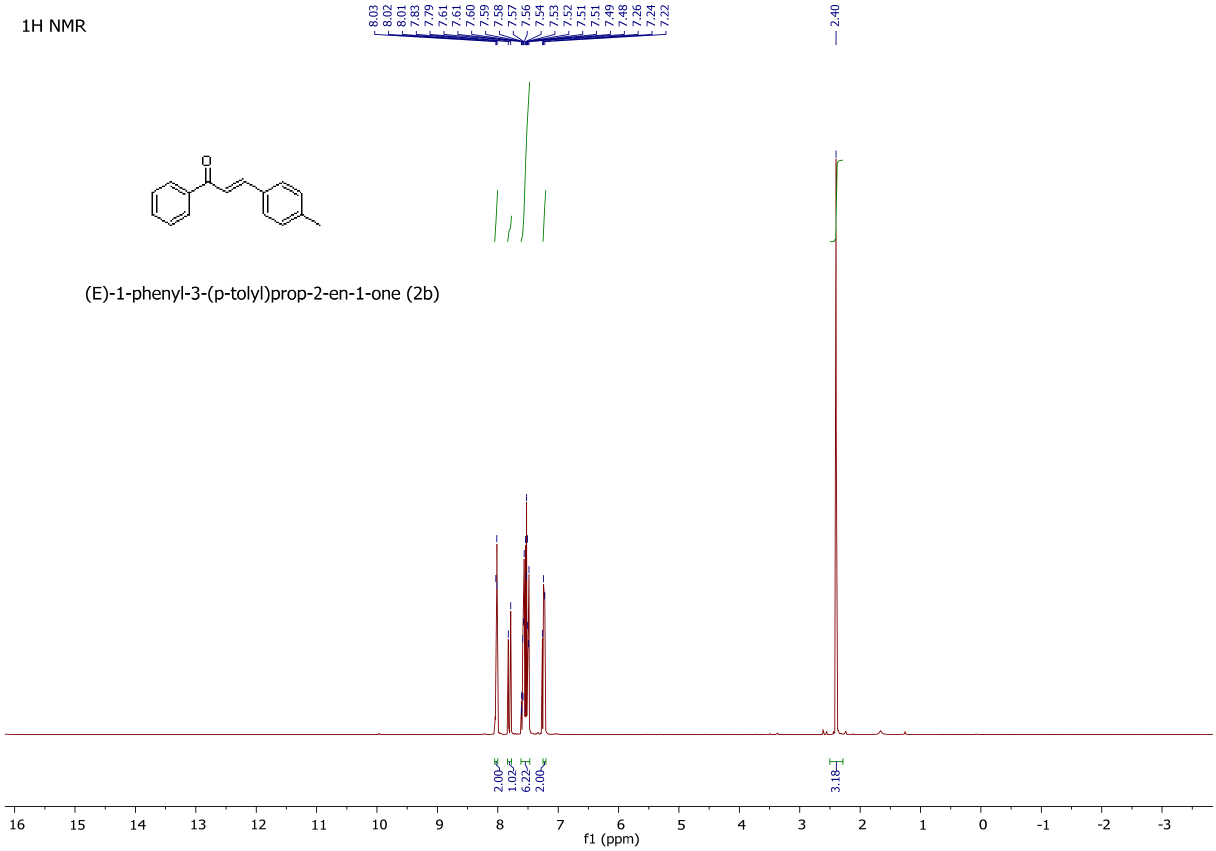

13C NMR

(E)-1-phenyl-3-(p-tolyl)prop-2-en-1-one (2b)

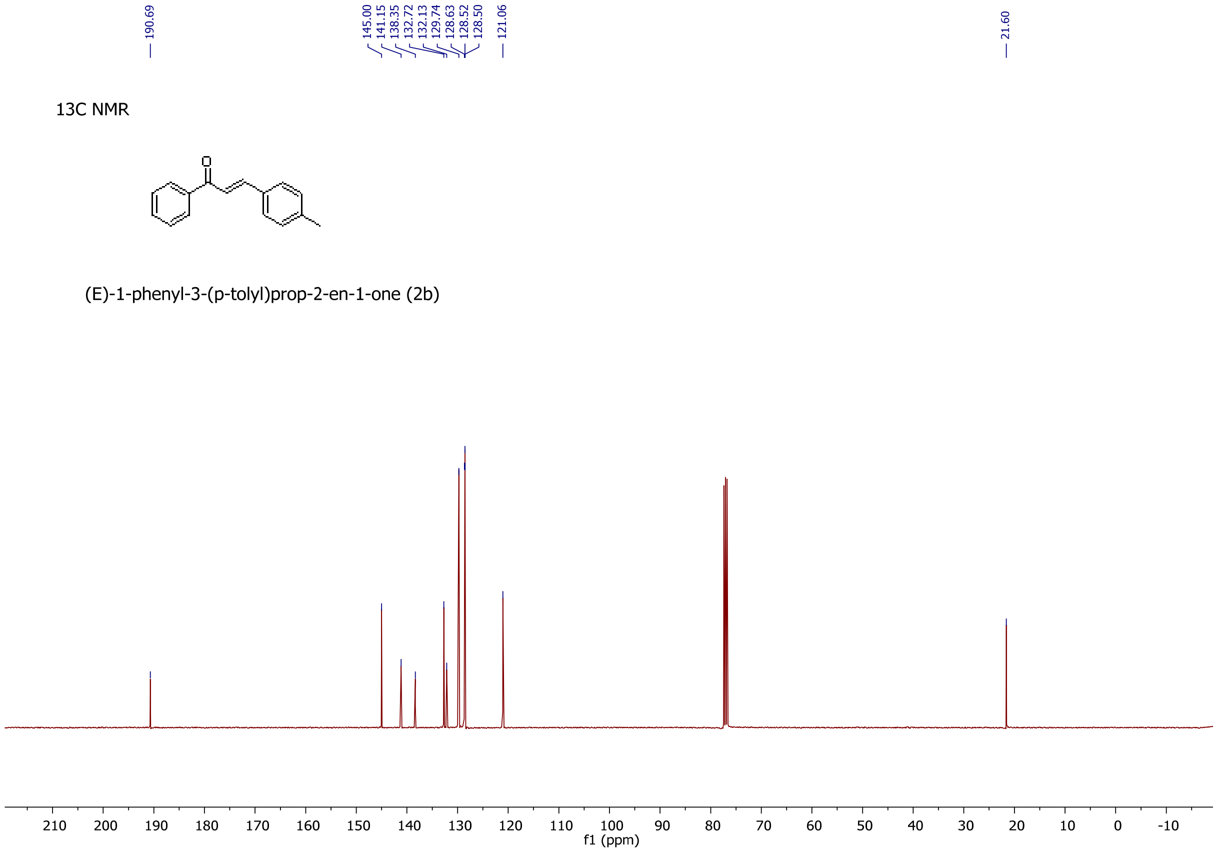

1H NMR

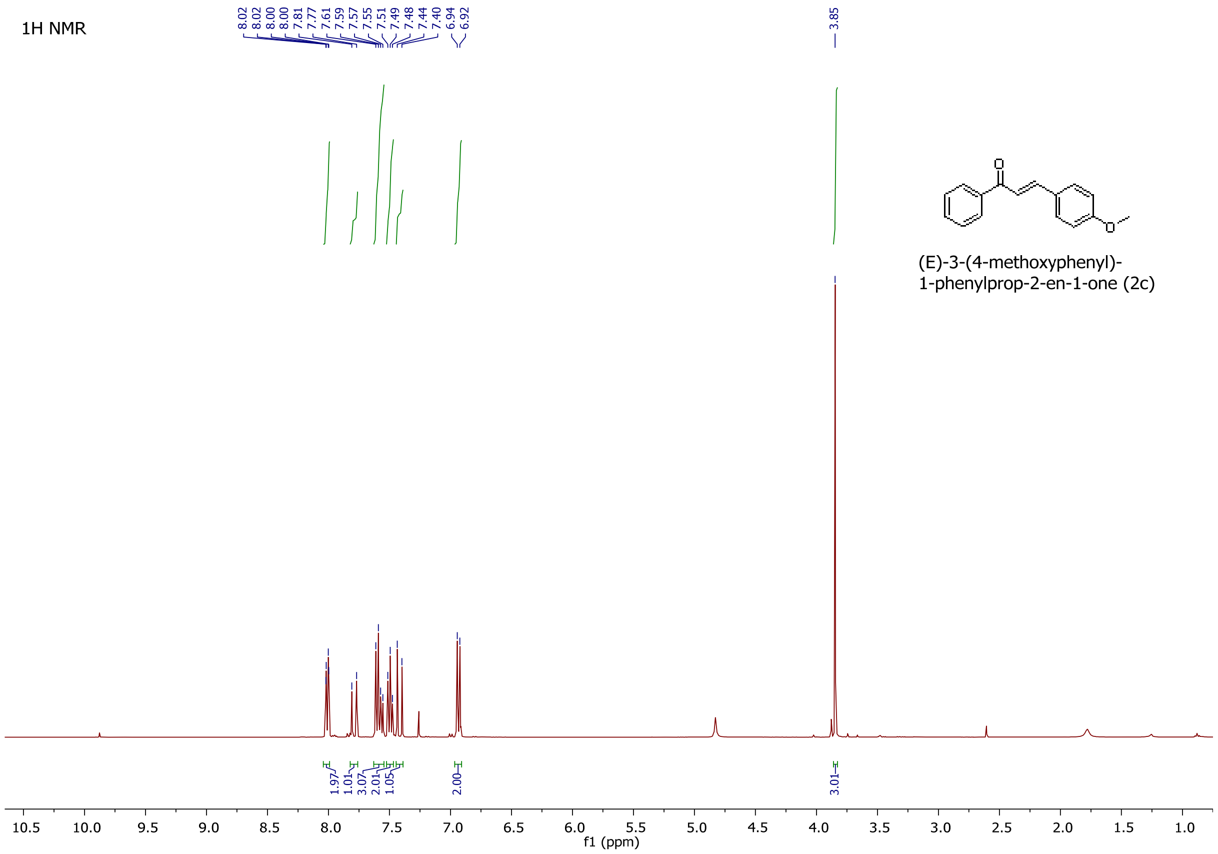

(E)-3-(4-methoxyphenyl)-
1-phenylprop-2-en-1-one (2c)

13C NMR

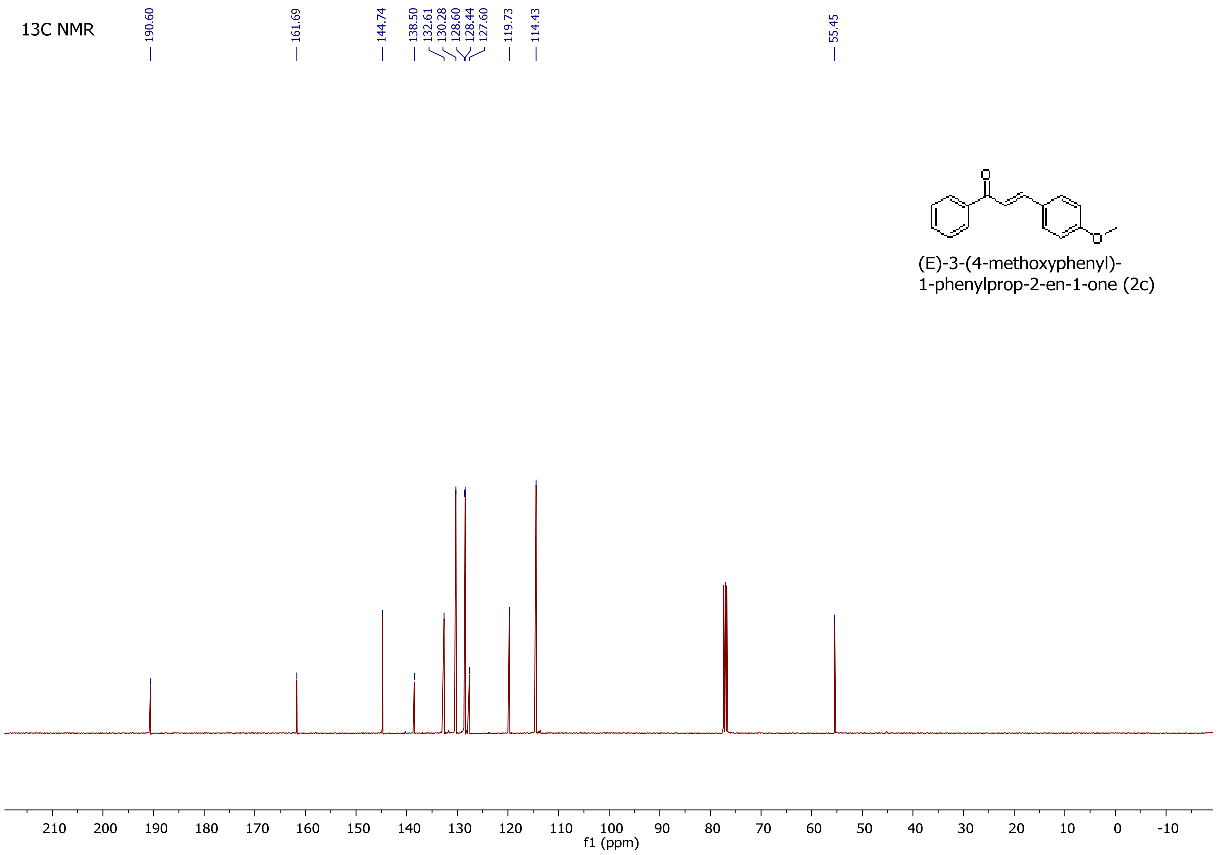

(E)-3-(4-methoxyphenyl)-
1-phenylprop-2-en-1-one (2c)

1H NMR

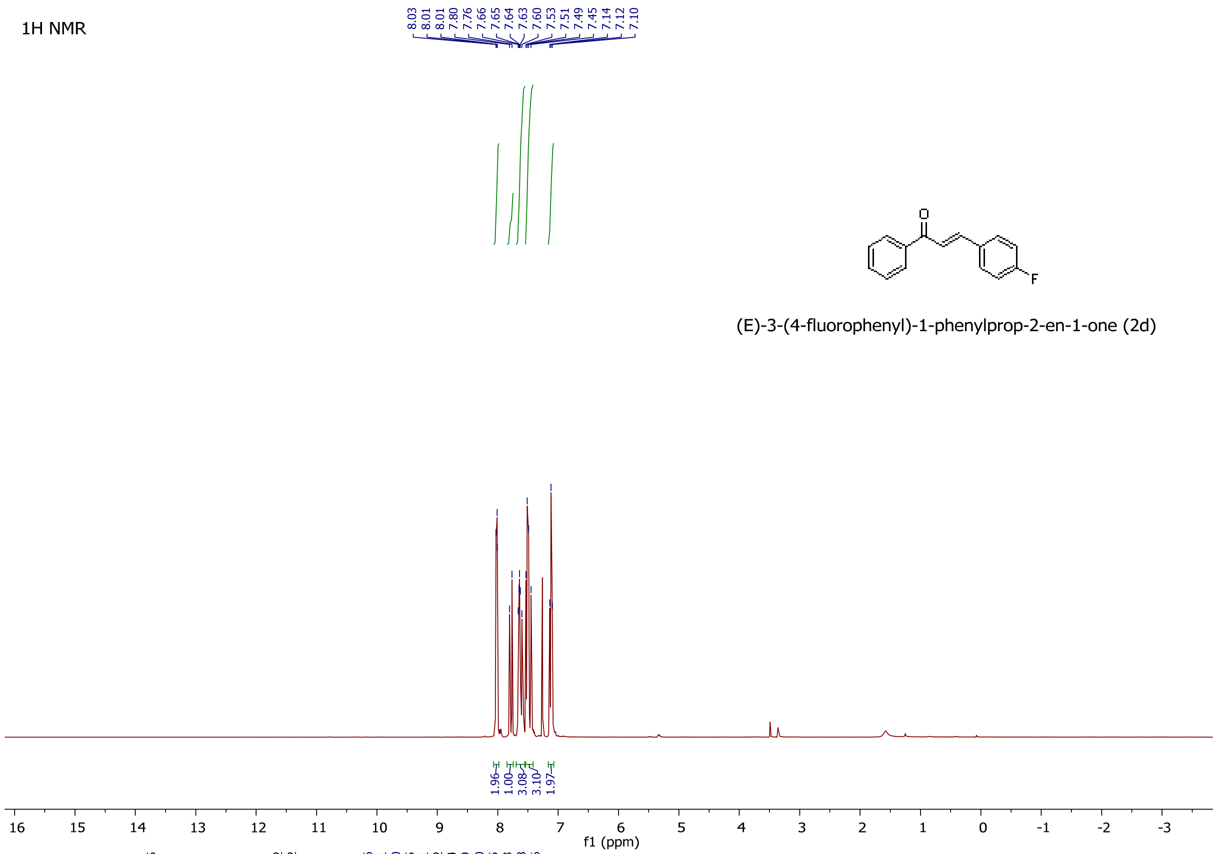

(E)-3-(4-fluorophenyl)-1-phenylprop-2-en-1-one (2d)

13C NMR

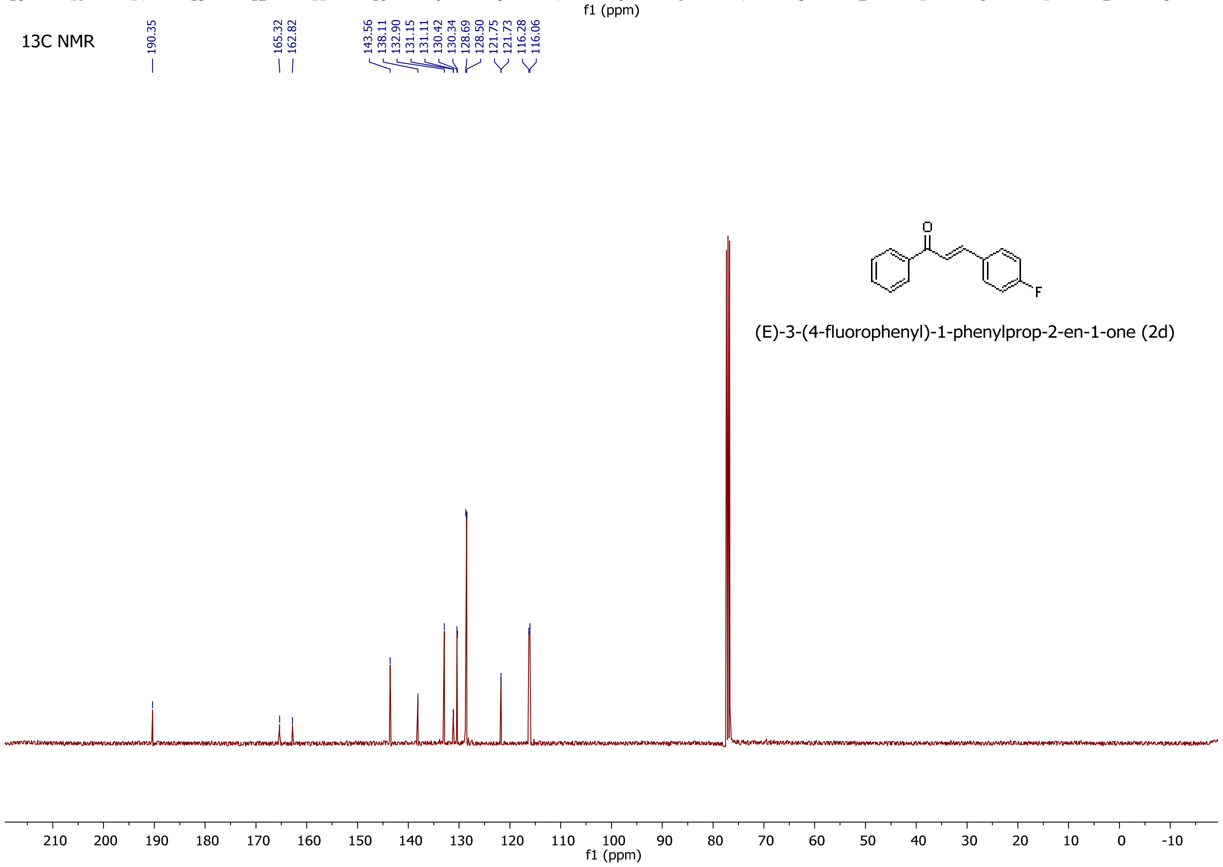

(E)-3-(4-fluorophenyl)-1-phenylprop-2-en-1-one (2d)

1H NMR

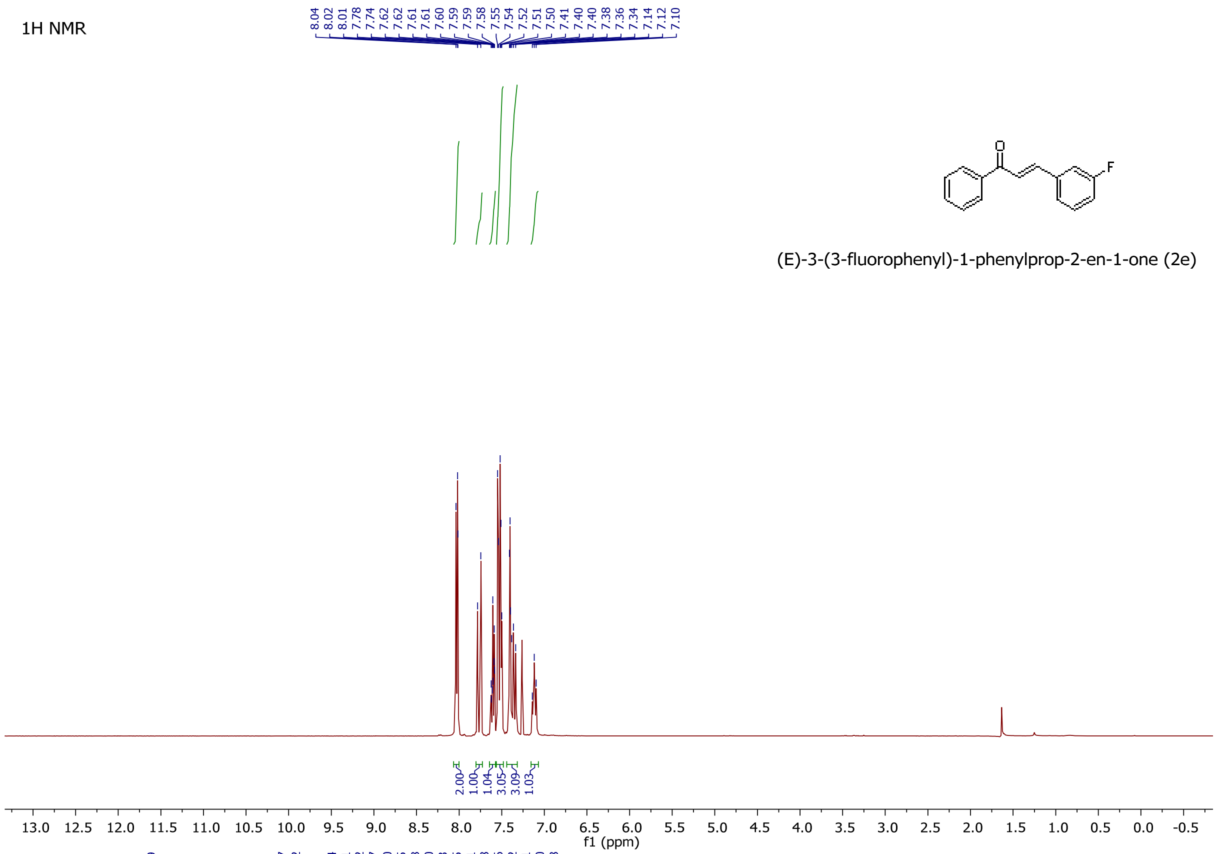

(E)-3-(3-fluorophenyl)-1-phenylprop-2-en-1-one (2e)

13C NMR

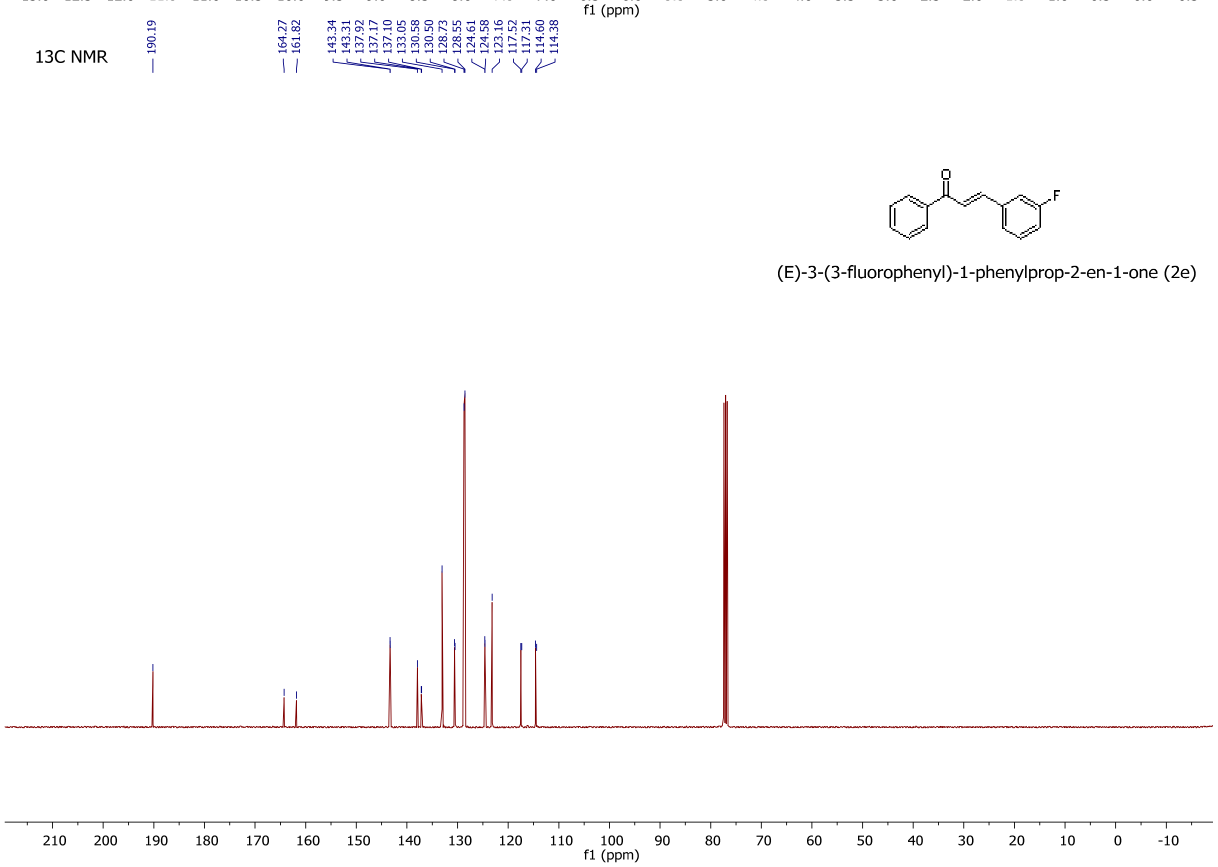

(E)-3-(3-fluorophenyl)-1-phenylprop-2-en-1-one (2e)

1H NMR

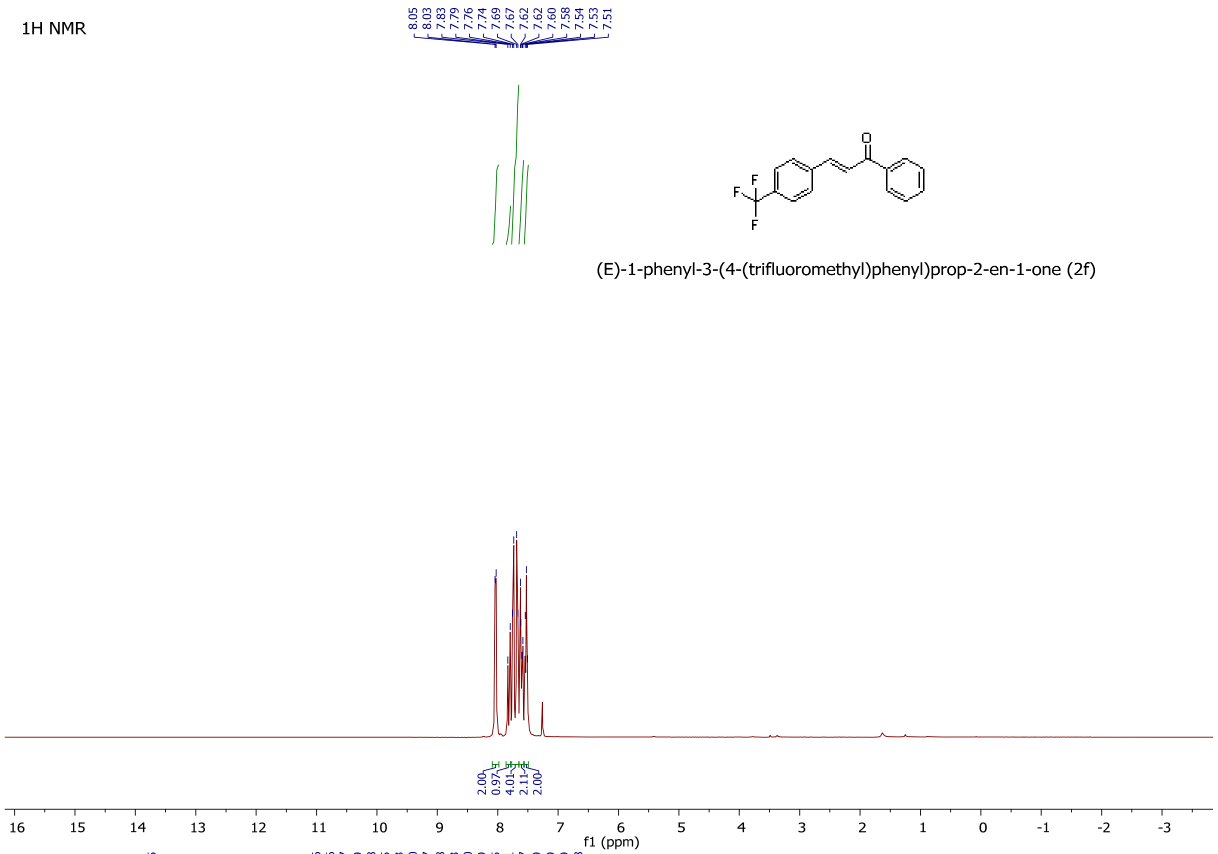

(E)-1-phenyl-3-(4-(trifluoromethyl)phenyl)prop-2-en-1-one (2f)

13C NMR

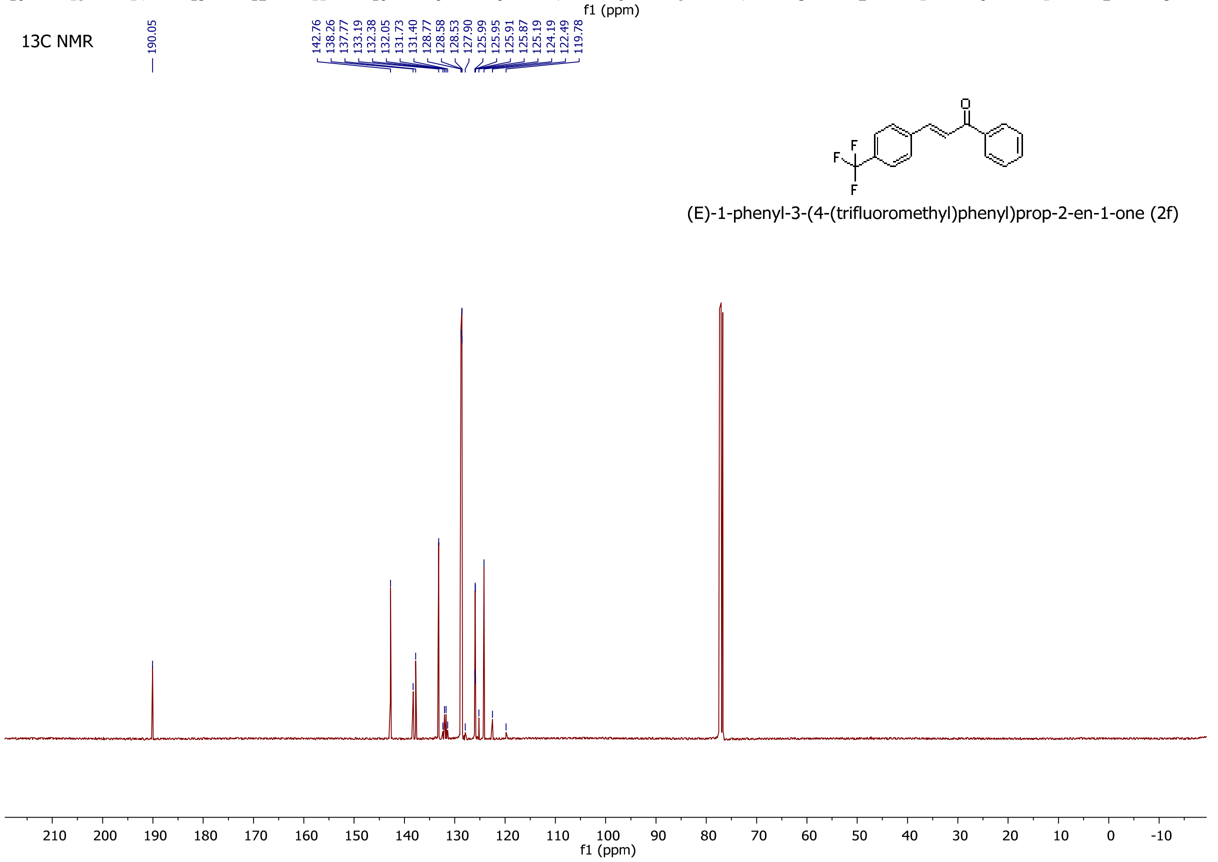

(E)-1-phenyl-3-(4-(trifluoromethyl)phenyl)prop-2-en-1-one (2f)

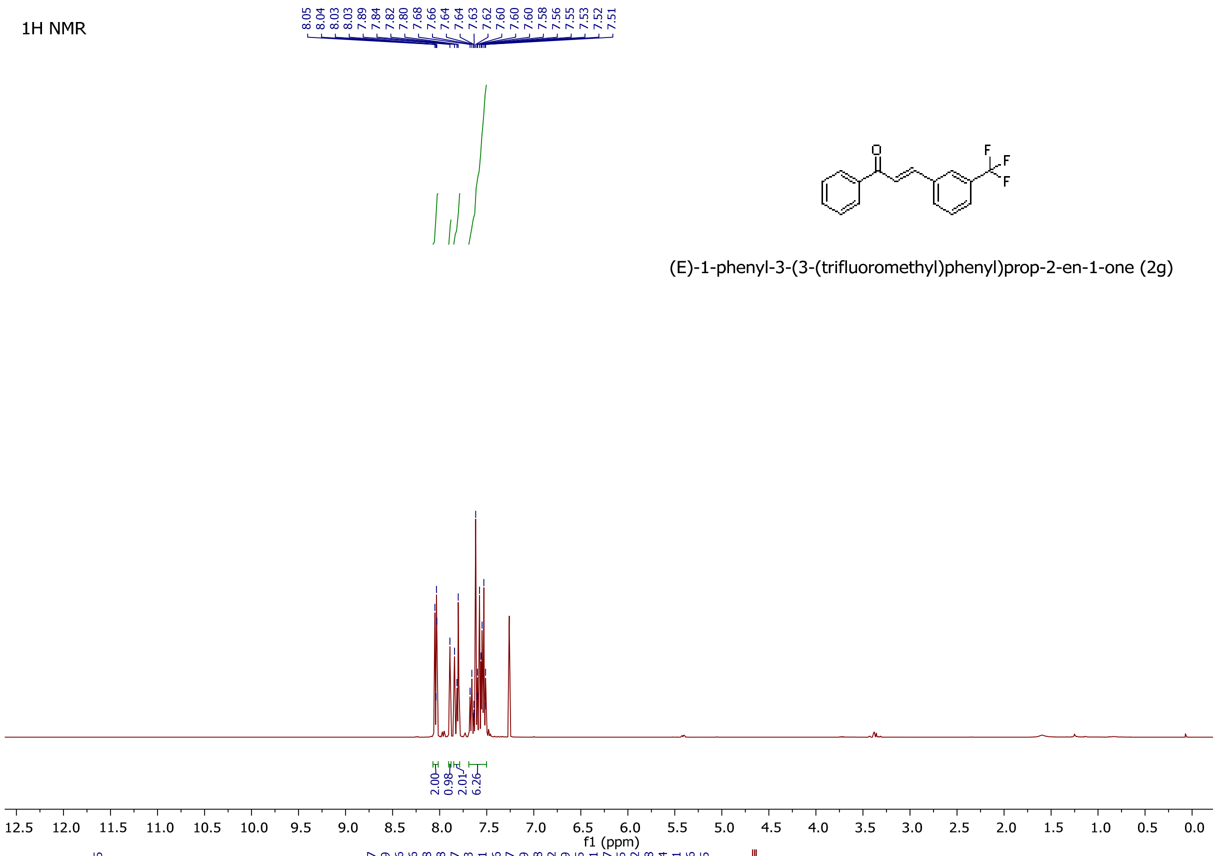
1H NMR
8.05 8.04 8.03 8.03 7.89 7.84 7.82 7.80 7.68 7.66 7.64 7.64 7.63 7.62 7.60 7.60 7.58 7.56 7.55 7.53 7.52 7.51
(E)-1-phenyl-3-(3-(trifluoromethyl)phenyl)prop-2-en-1-one (2g)
2.00 0.98 2.01 6.26
f1 (ppm)

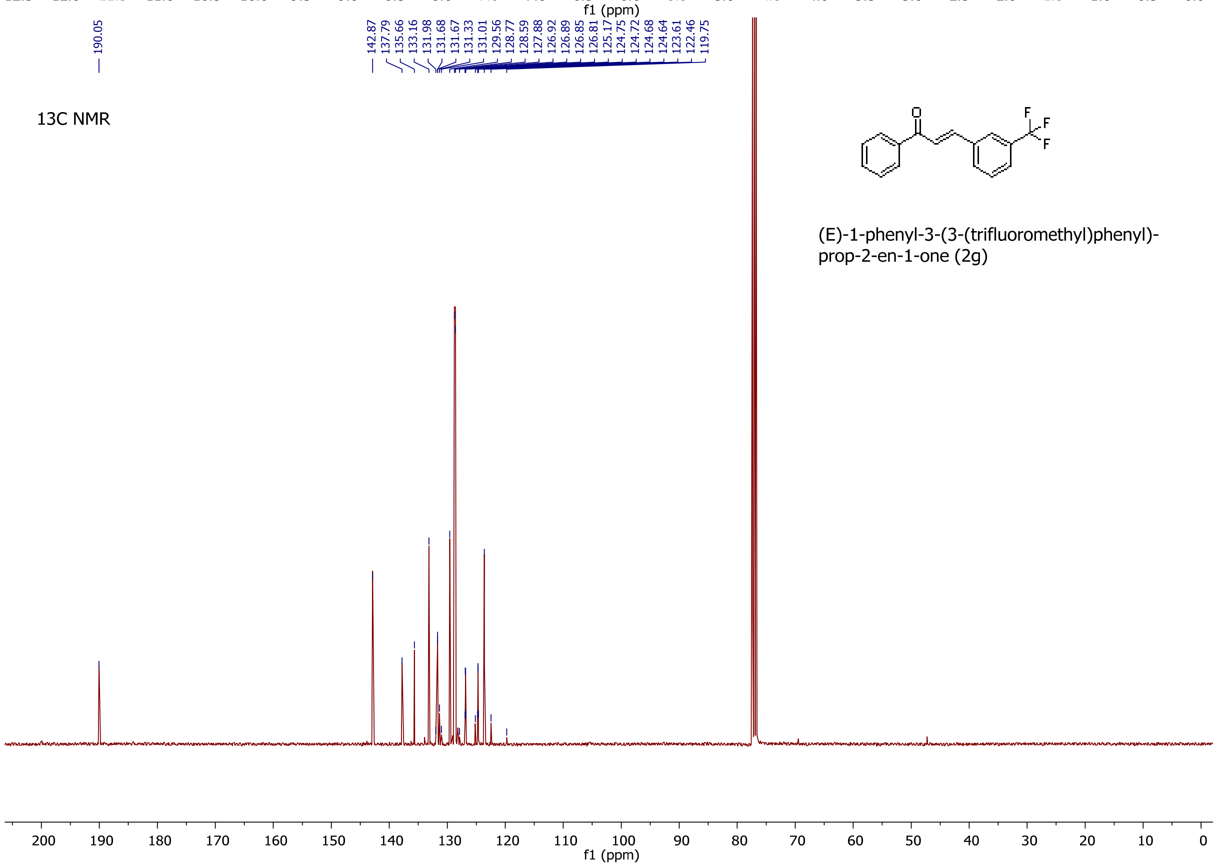
190.05
142.87 137.79 135.66 133.16 131.98 131.68 131.67 131.33 131.01 129.56 128.77 128.59 127.88 126.92 126.89 126.85 126.81 125.17 124.75 124.72 124.68 124.64 123.61 122.46 119.75
13C NMR
(E)-1-phenyl-3-(3-(trifluoromethyl)phenyl)-
prop-2-en-1-one (2g)
f1 (ppm)

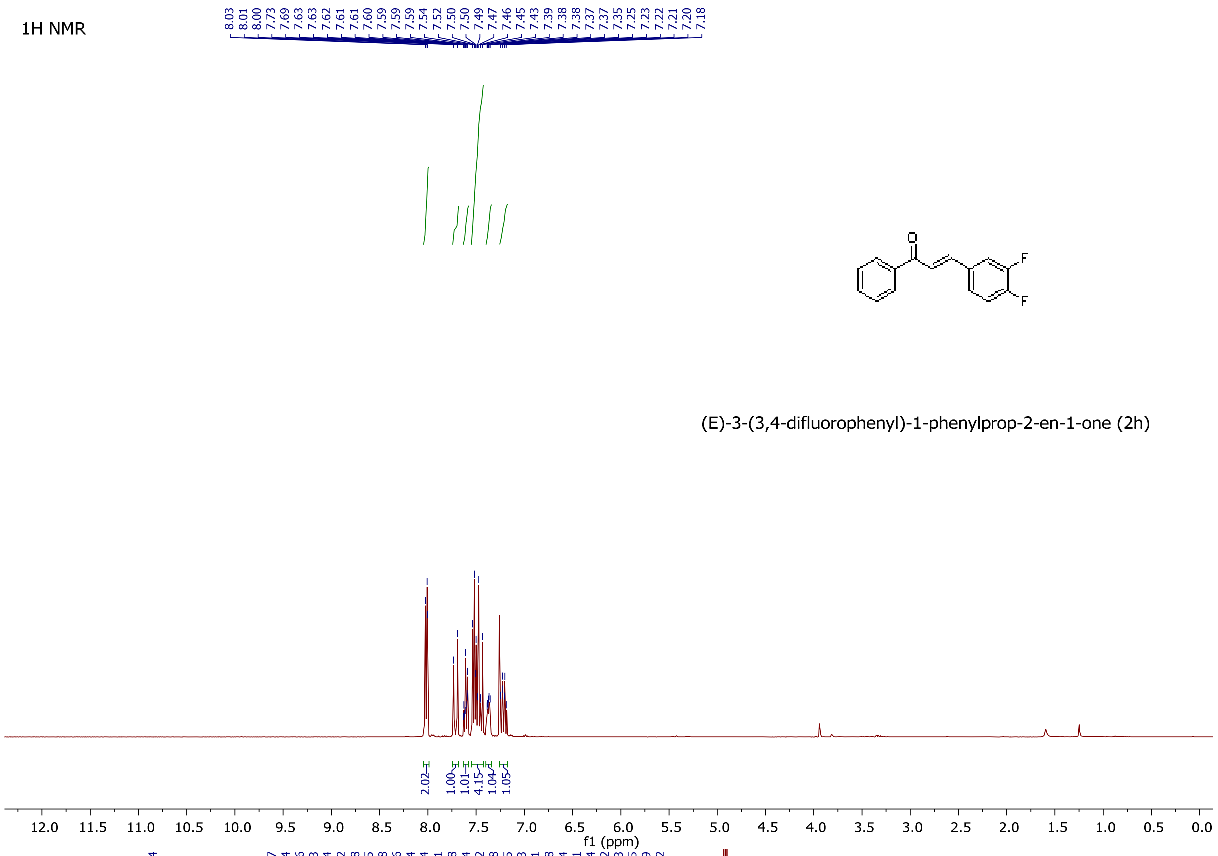
1H NMR
8.03
8.01
8.00
7.73
7.69
7.63
7.63
7.62
7.61
7.61
7.60
7.59
7.59
7.59
7.54
7.52
7.50
7.50
7.49
7.47
7.46
7.45
7.43
7.39
7.38
7.38
7.37
7.35
7.25
7.23
7.22
7.21
7.20
7.18
O
F
F
(E)-3-(3,4-difluorophenyl)-1-phenylprop-2-en-1-one (2h)
2.02
1.00
1.01
4.15
1.04
1.05
12.0 11.5 11.0 10.5 10.0 9.5 9.0 8.5 8.0 7.5 7.0 6.5 6.0 5.5 5.0 4.5 4.0 3.5 3.0 2.5 2.0 1.5 1.0 0.5 0.0
f1 (ppm)

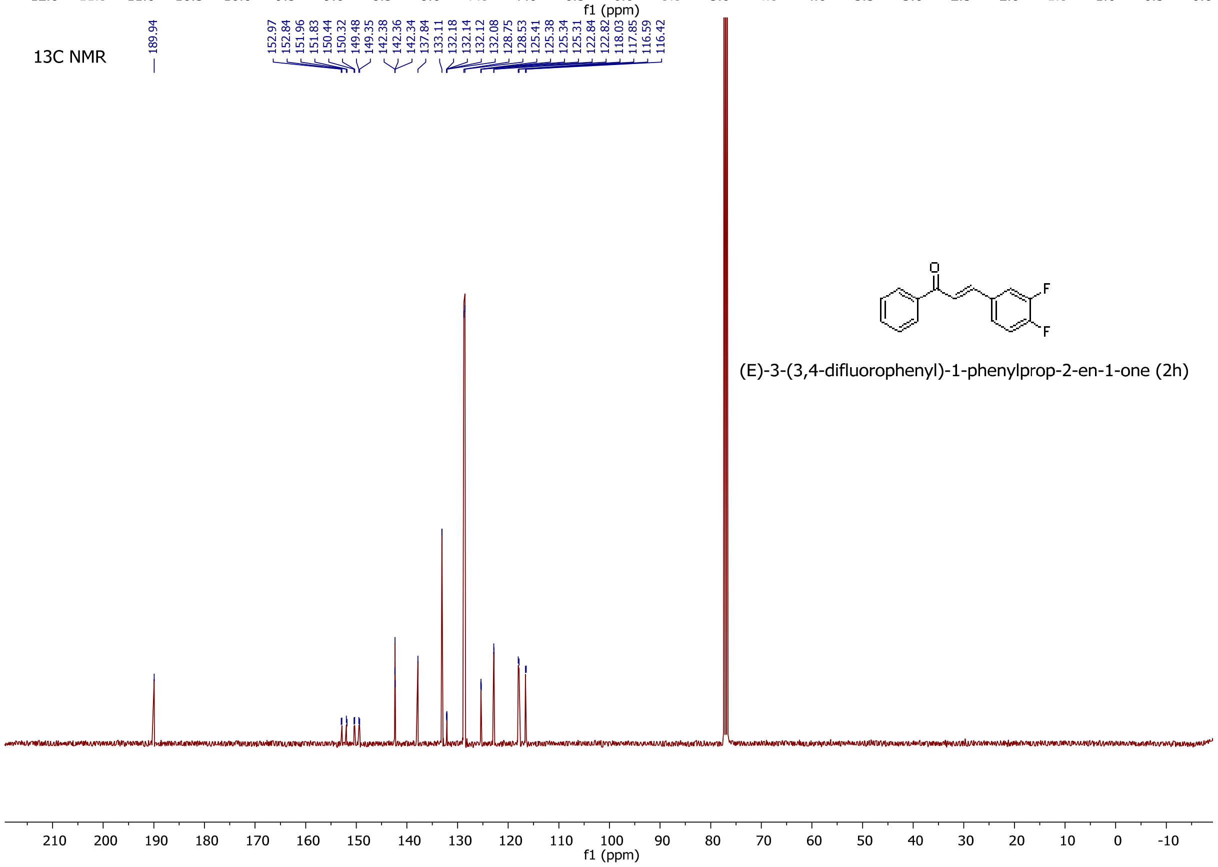
13C NMR
189.94
152.97
152.84
151.96
151.83
150.44
150.32
149.48
149.35
142.38
142.36
142.34
137.84
133.11
132.18
132.14
132.12
132.08
128.75
128.53
125.41
125.38
125.34
125.31
122.84
122.82
118.03
117.85
116.59
116.42
O
F
F
(E)-3-(3,4-difluorophenyl)-1-phenylprop-2-en-1-one (2h)
210 200 190 180 170 160 150 140 130 120 110 100 90 80 70 60 50 40 30 20 10 0 -10
f1 (ppm)

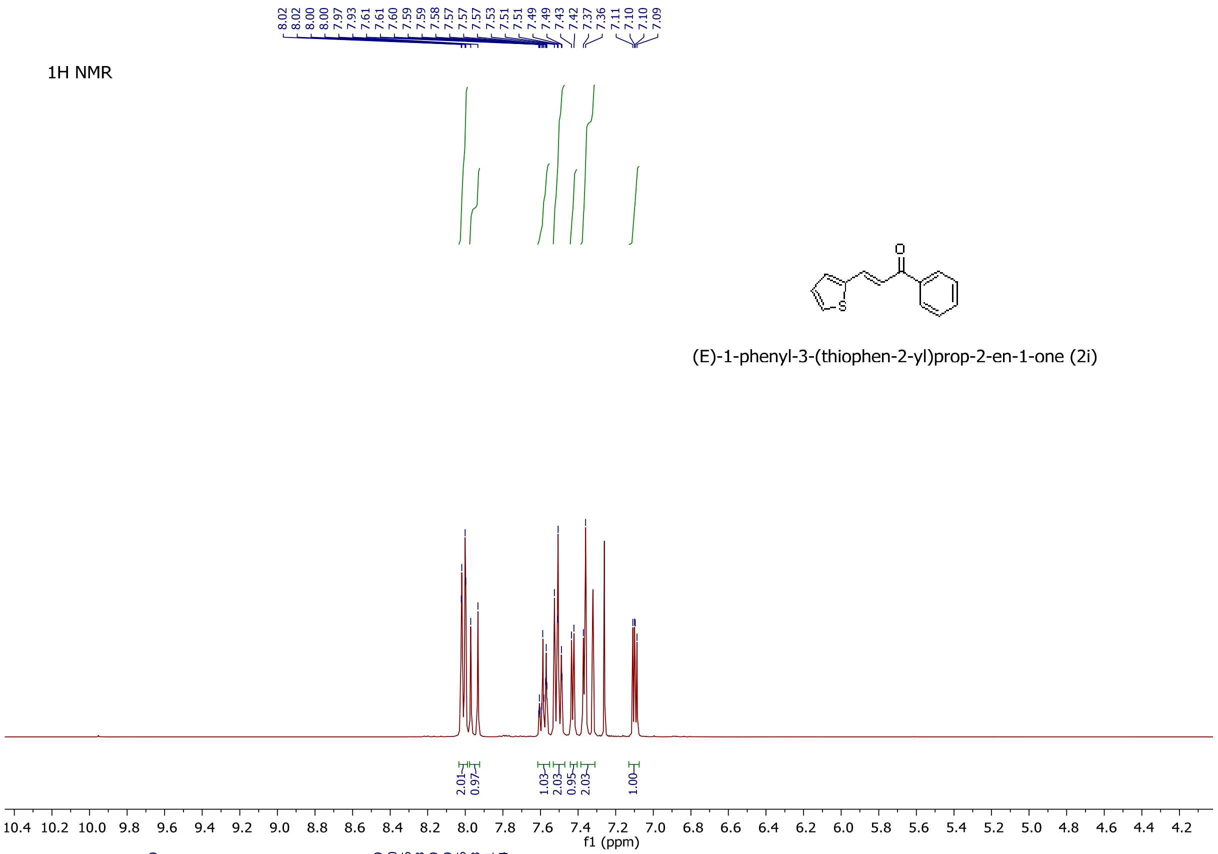
1H NMR
8.02
8.02
8.00
7.97
7.93
7.61
7.61
7.60
7.59
7.59
7.58
7.57
7.57
7.57
7.53
7.51
7.51
7.49
7.49
7.43
7.42
7.37
7.36
7.11
7.10
7.10
7.09
O
S
(E)-1-phenyl-3-(thiophen-2-yl)prop-2-en-1-one (2i)
2.01
0.97
1.03
2.03
0.95
2.03
1.00
f1 (ppm)

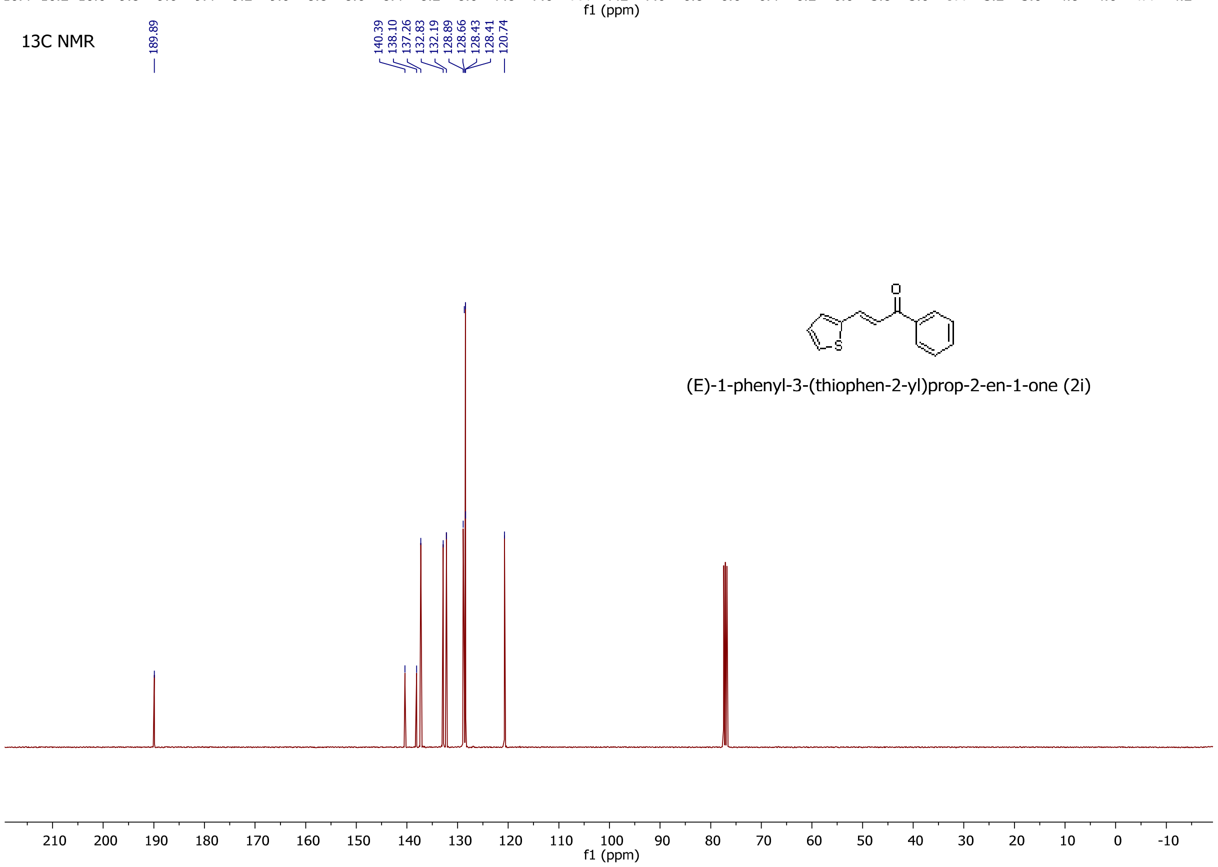
13C NMR
189.89
140.39
138.10
137.26
132.83
132.19
128.89
128.66
128.43
128.41
120.74
O
S
(E)-1-phenyl-3-(thiophen-2-yl)prop-2-en-1-one (2i)
f1 (ppm)

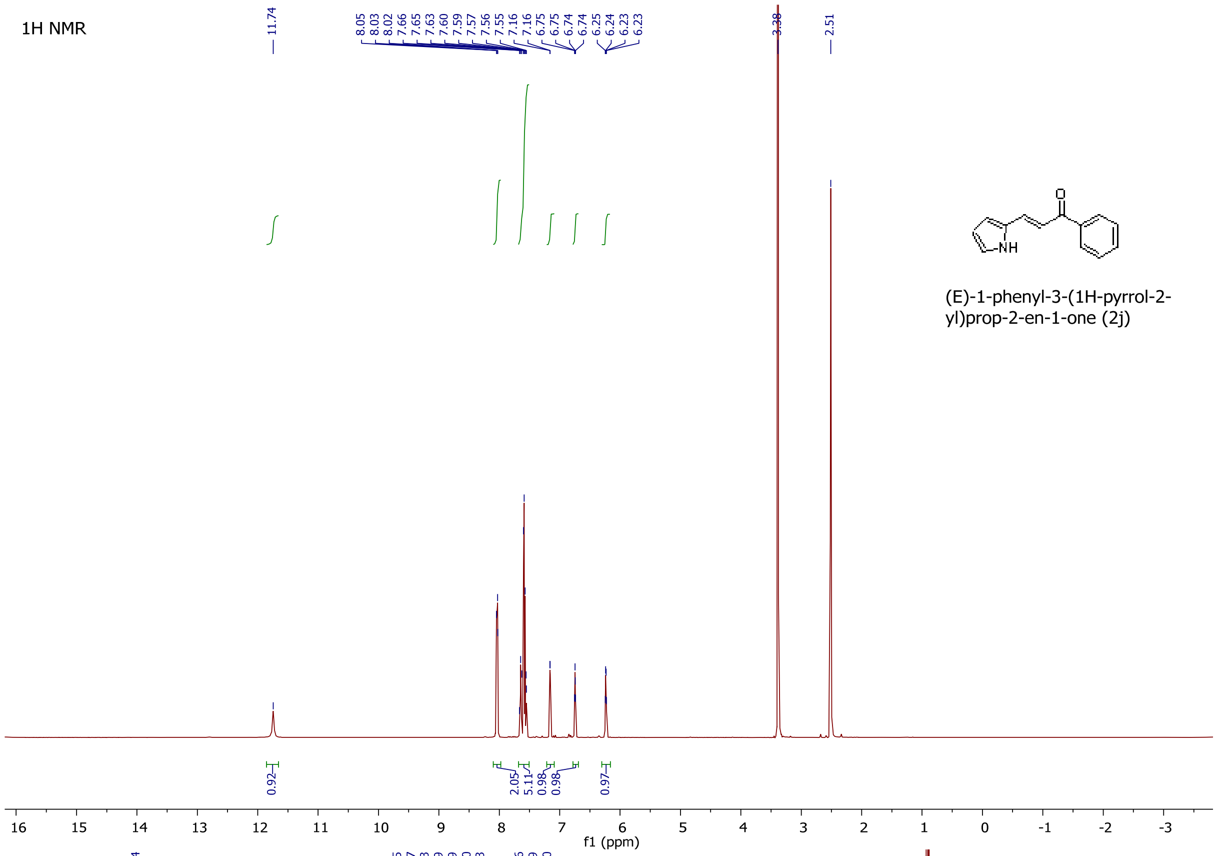
1H NMR
11.74
8.05
8.03
8.02
7.66
7.65
7.63
7.60
7.59
7.57
7.56
7.55
7.16
7.16
6.75
6.75
6.74
6.74
6.25
6.24
6.23
6.23
3.38
2.51
NH
O
(E)-1-phenyl-3-(1H-pyrrol-2-yl)prop-2-en-1-one (2j)
0.92
2.05
5.11
0.98
0.98
0.97
16 15 14 13 12 11 10 9 8 7 6 5 4 3 2 1 0 -1 -2 -3
f1 (ppm)

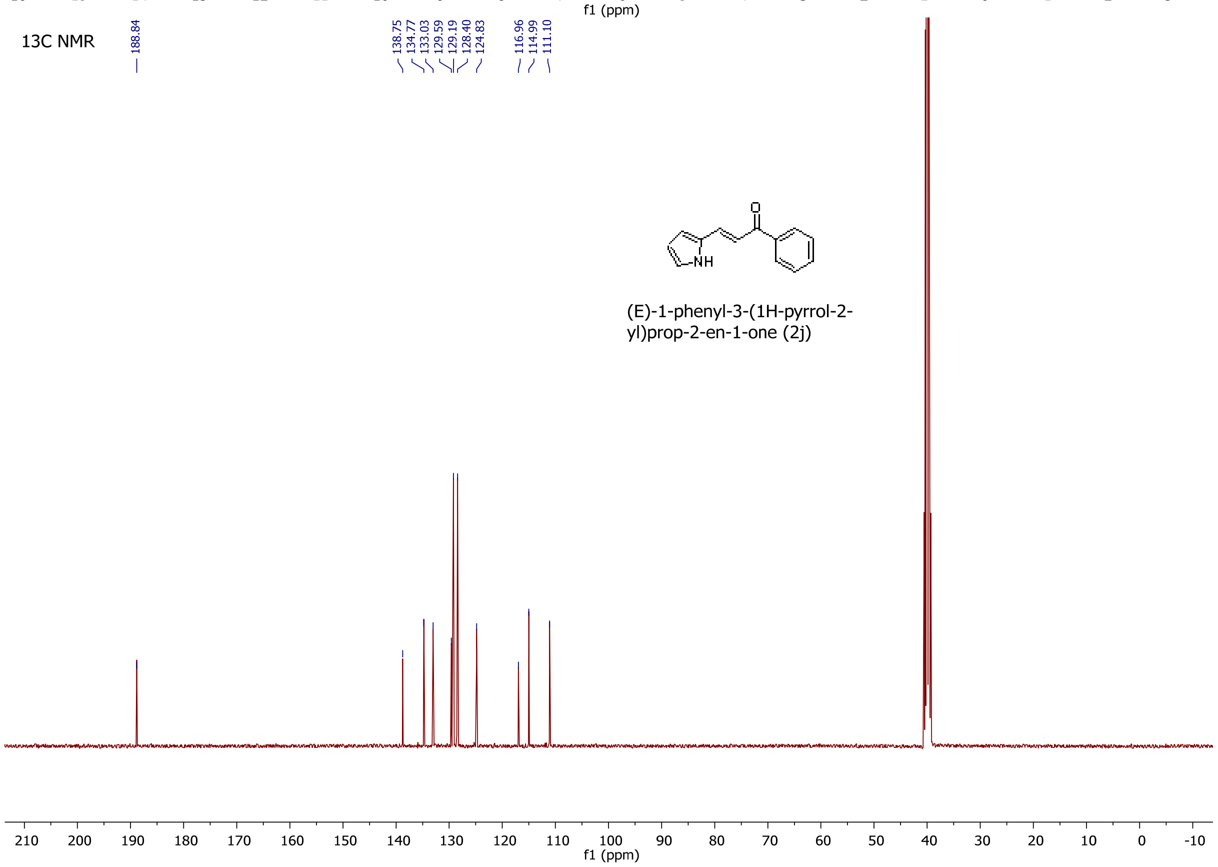
13C NMR
188.84
138.75
134.77
133.03
129.59
129.19
128.40
124.83
116.96
114.99
111.10
NH
O
(E)-1-phenyl-3-(1H-pyrrol-2-yl)prop-2-en-1-one (2j)
210 200 190 180 170 160 150 140 130 120 110 100 90 80 70 60 50 40 30 20 10 0 -10
f1 (ppm)

1H NMR

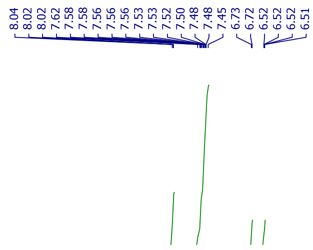

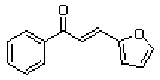

(E)-1-phenyl-3-(thiophen-2-yl)prop-2-en-1-one (2k)

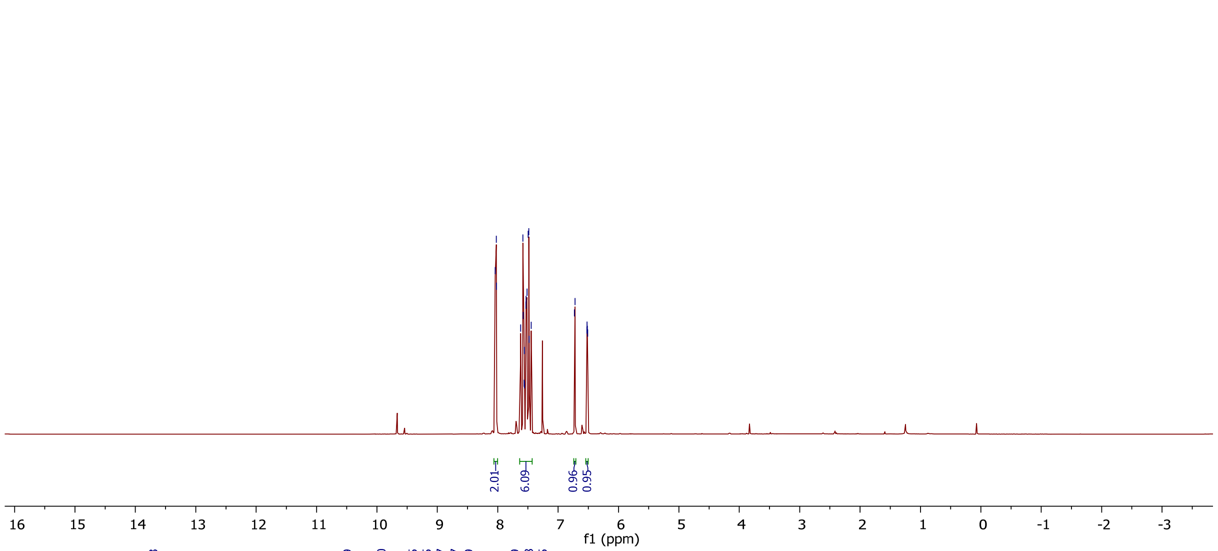

13C NMR

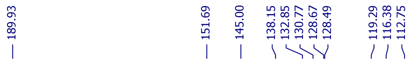

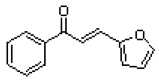

(E)-1-phenyl-3-(thiophen-2-yl)prop-2-en-1-one (2k)

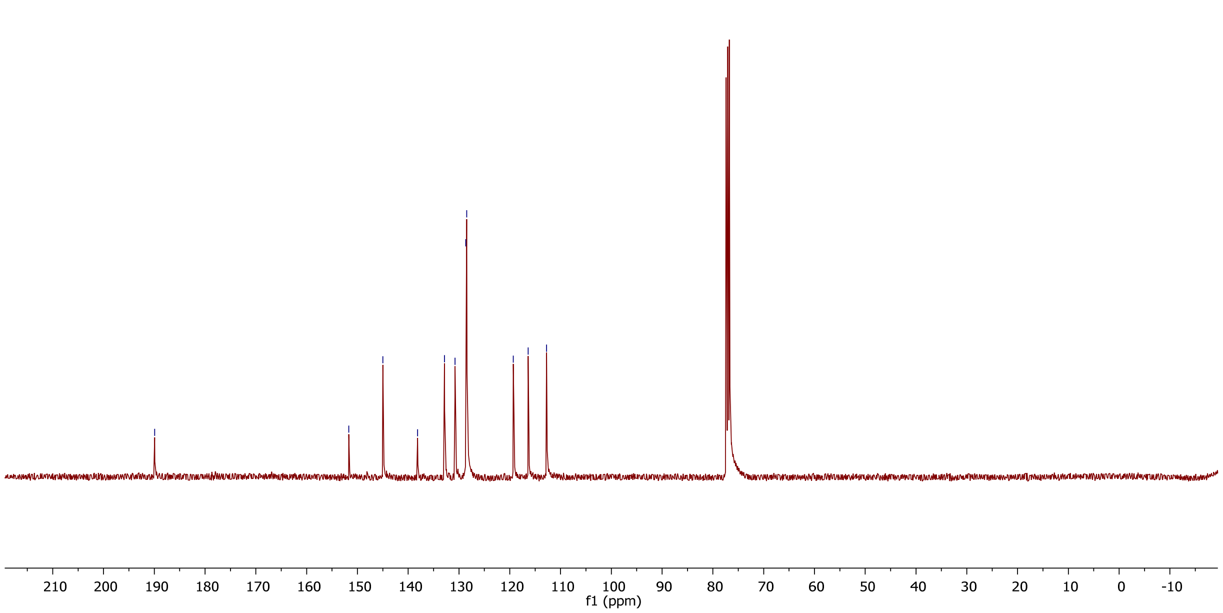

1H NMR

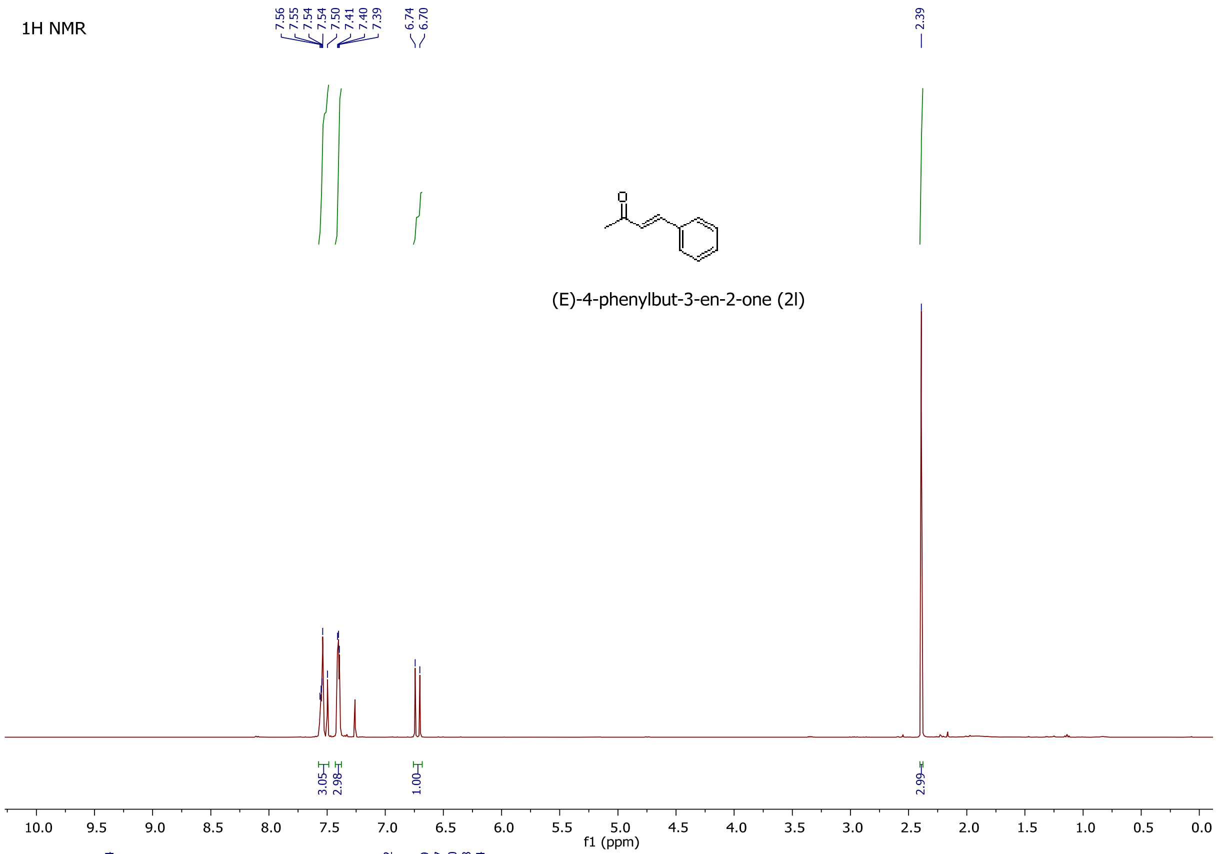

(E)-4-phenylbut-3-en-2-one (2l)

13C NMR

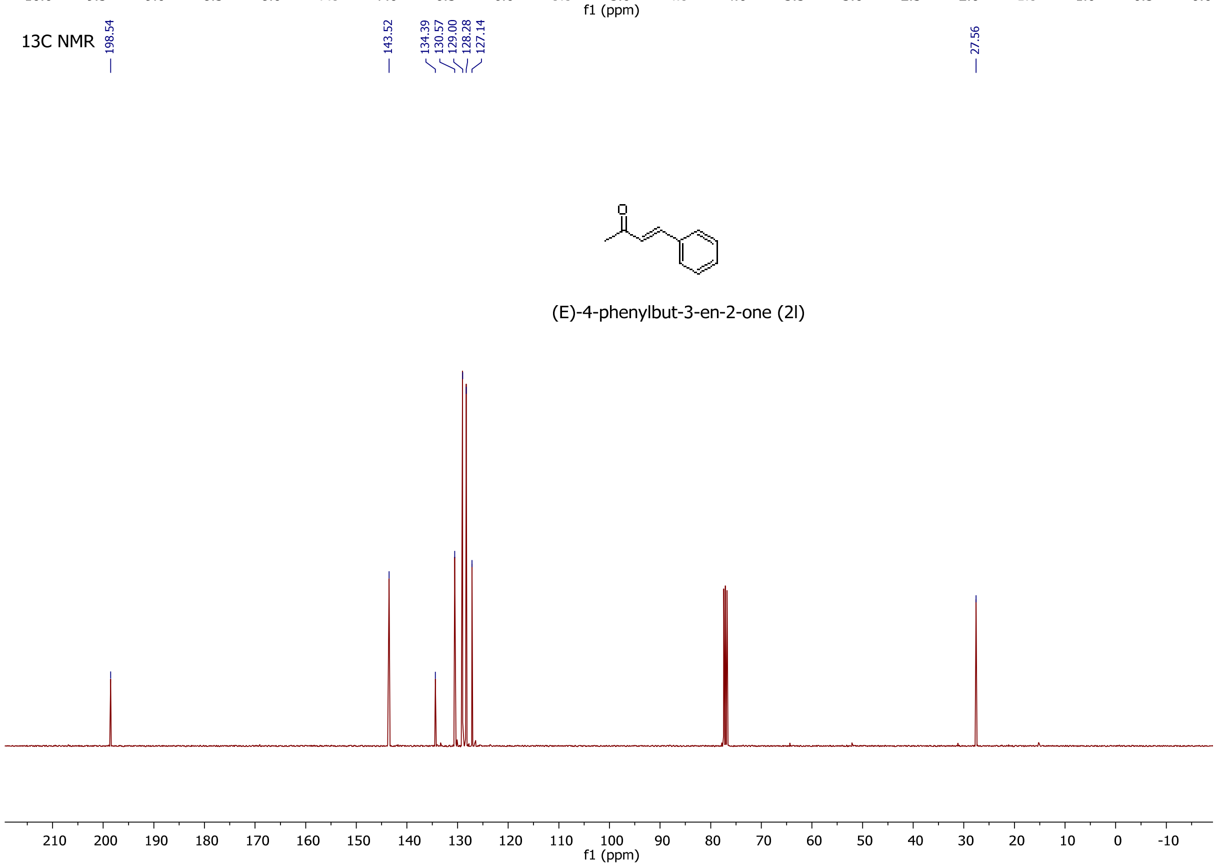

(E)-4-phenylbut-3-en-2-one (2l)

1H NMR

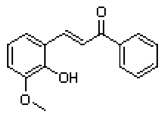

(E)-3-(2-hydroxy-3-methoxyphenyl)-1-phenylprop-2-en-1-one (2p)

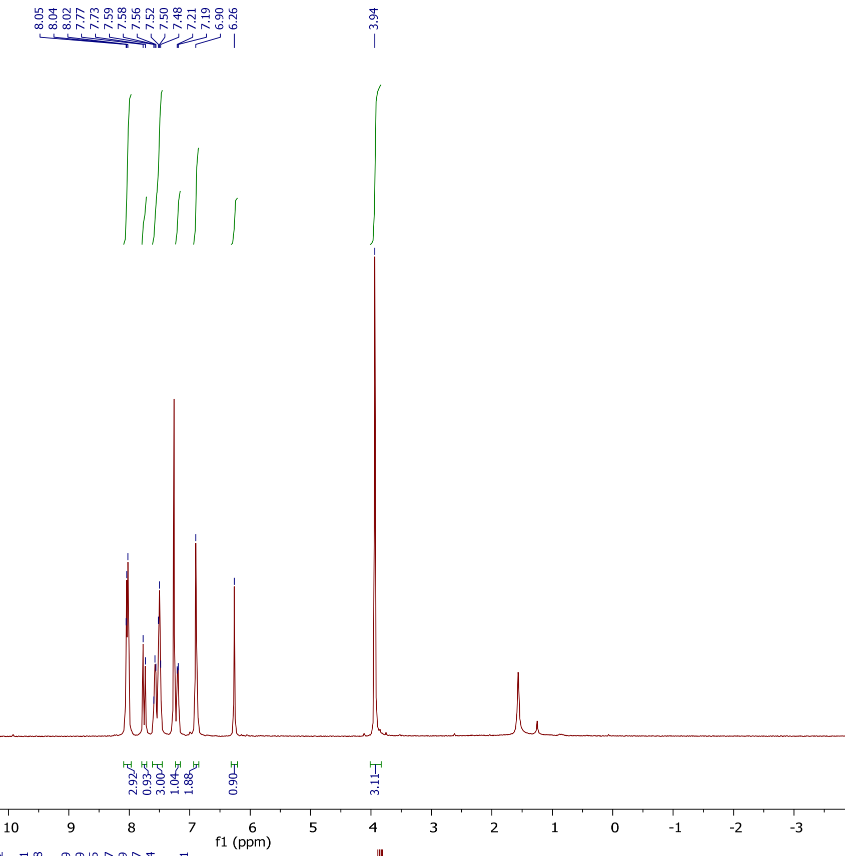

13C NMR

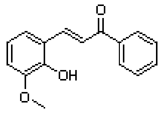

(E)-3-(2-hydroxy-3-methoxyphenyl)-1-phenylprop-2-en-1-one (2p)

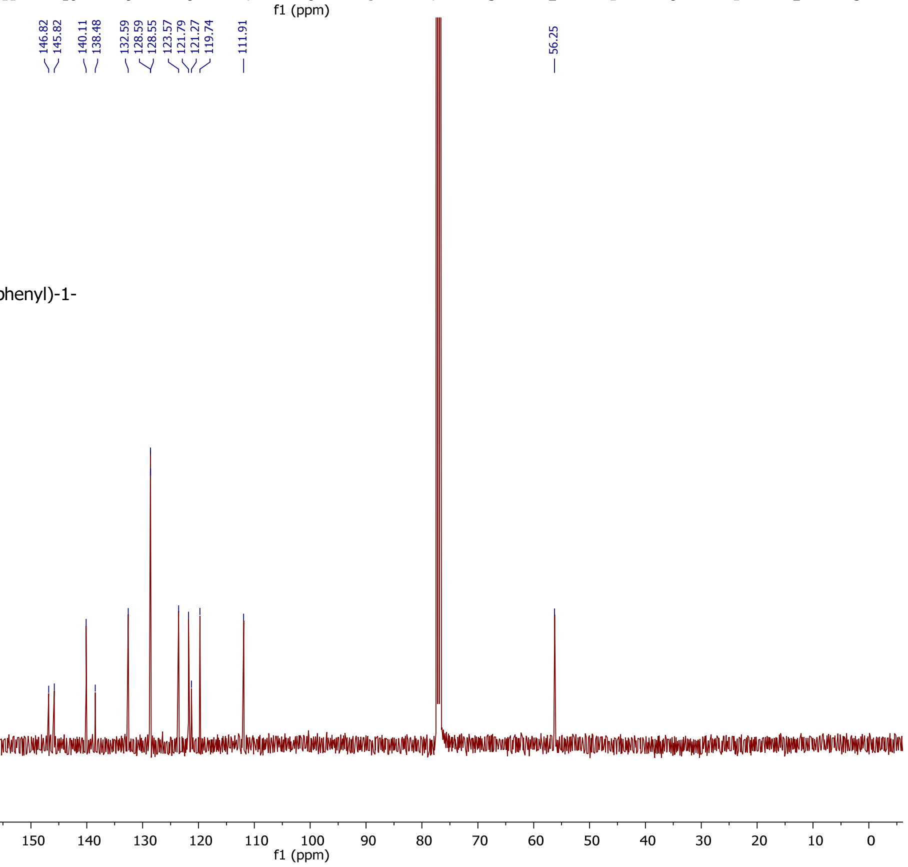

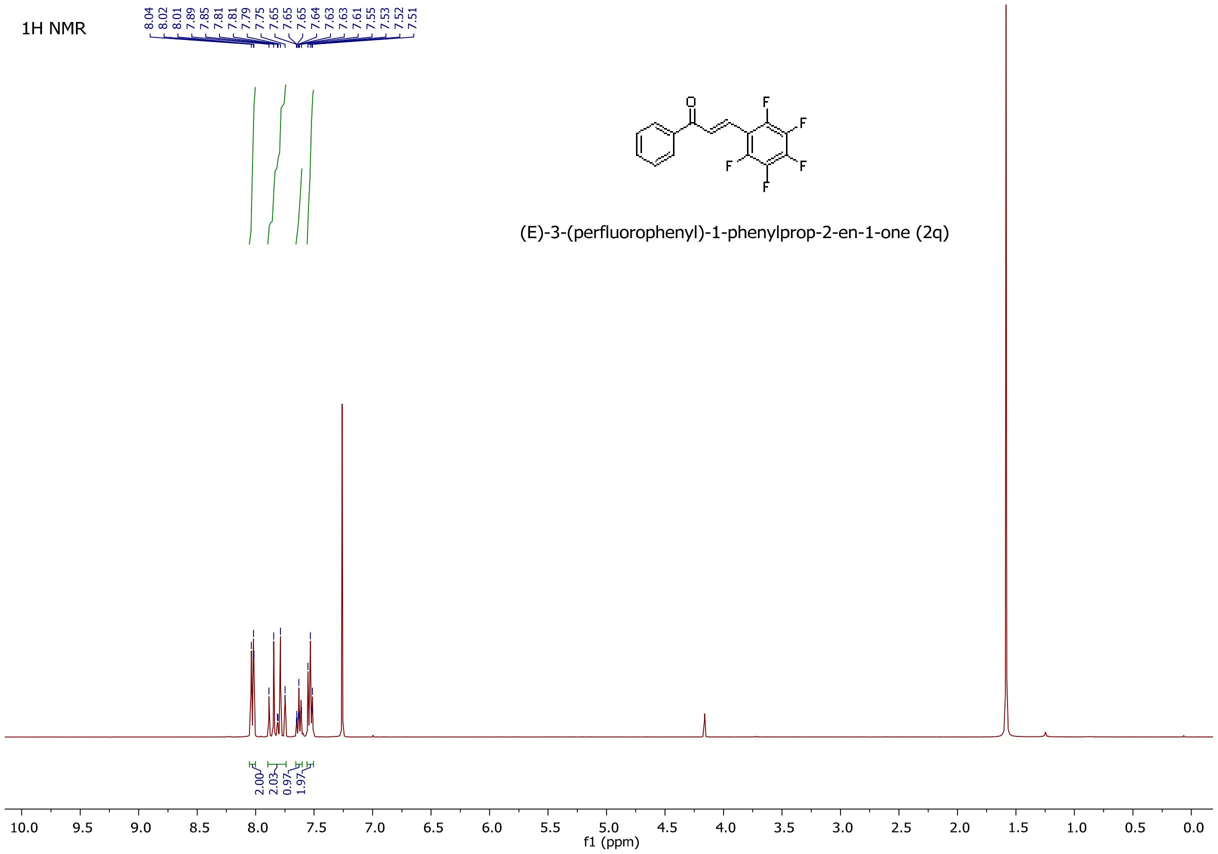
1H NMR
8.04 8.02 8.01 7.89 7.85 7.81 7.79 7.75 7.65 7.65 7.65 7.64 7.63 7.61 7.55 7.53 7.52 7.51
(E)-3-(perfluorophenyl)-1-phenylprop-2-en-1-one (2q)
2.00 2.03 0.97 1.97
10.0 9.5 9.0 8.5 8.0 7.5 7.0 6.5 6.0 5.5 5.0 4.5 4.0 3.5 3.0 2.5 2.0 1.5 1.0 0.5 0.0
f1 (ppm)

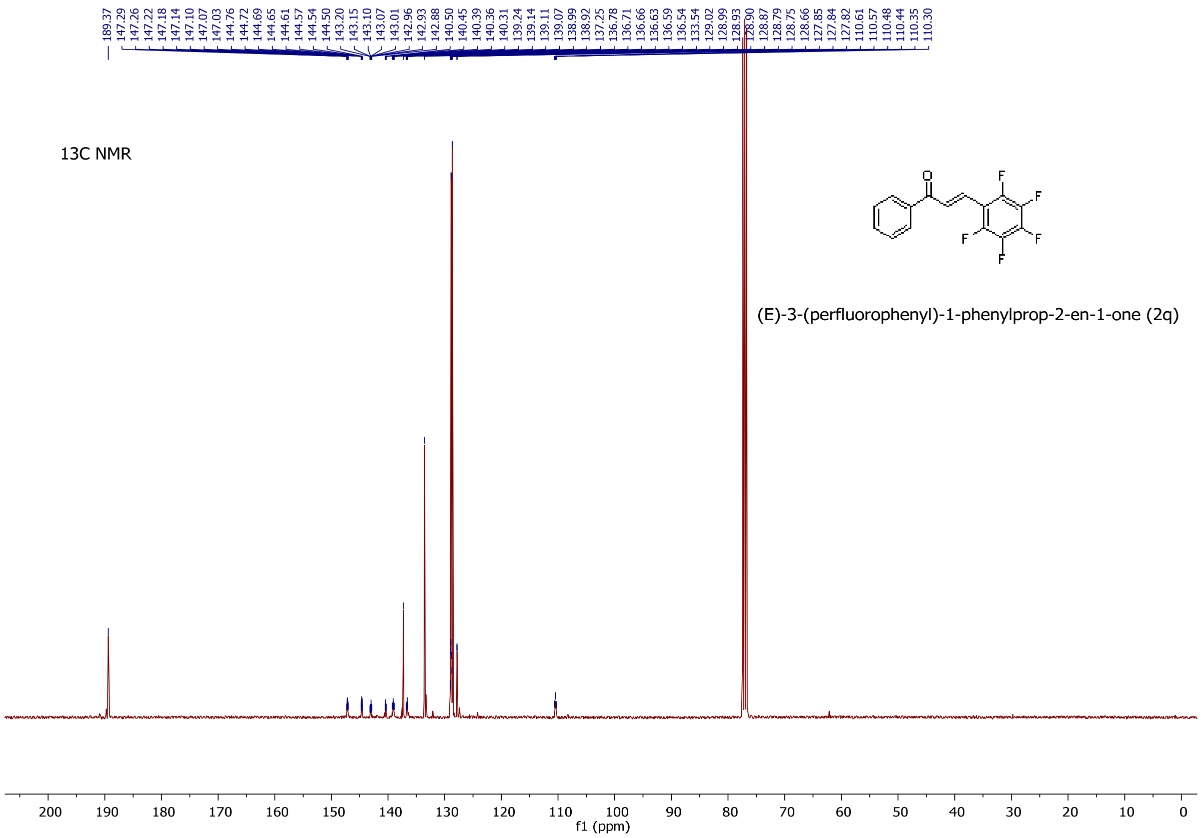
189.37 147.29 147.26 147.22 147.18 147.14 147.10 147.07 147.03 144.76 144.72 144.69 144.65 144.61 144.57 144.54 144.50 143.20 143.15 143.10 143.07 143.01 142.96 142.93 142.88 140.50 140.45 140.39 140.36 140.31 139.24 139.14 139.11 139.07 138.99 138.92 137.25 136.78 136.71 136.66 136.63 136.59 136.54 133.54 129.02 128.99 128.93 128.90 128.87 128.79 128.75 128.66 127.85 127.84 127.82 110.61 110.57 110.48 110.44 110.35 110.30
13C NMR
(E)-3-(perfluorophenyl)-1-phenylprop-2-en-1-one (2q)
200 190 180 170 160 150 140 130 120 110 100 90 80 70 60 50 40 30 20 10 0
f1 (ppm)

General method of 5,6-dihydropyrrolo[2,1-a]isoquinolines (3a-o) and 9p preparation.

A glass tube with rubber-lined cap was evacuated and filled with argon three times. To this tube THIQ (20mg, 150 µmol), corresponding chalcone (50 µmol), acetone (9mg, 150 µmol), K-PHI (5 mg) and acetonitrile (2 mL) were added (Figure 2). The resulting mixture was stirred at 80°C under irradiation of blue LED (λ=461nm, 51.7±0.03 mW·cm^{-2}) for 20 hours. Then the reaction mixture was cooled to room temperature and centrifuged, clear solution was separated and solid residue was washed with acetonitrile (2 mL) and centrifuged again. Organic solutions were combined and evaporated to dryness. The residue after evaporation was purified by silica gel column chromatography using mixture of hexane/diethyl ether (98:2) as an eluent.

1,3-diphenyl-5,6-dihydropyrrolo[2,1-a]isoquinoline*[11] *(3a).

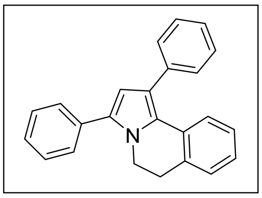

Yellowish oil (yield 82%). ^{1}H NMR (400 MHz, CD_3CN) δ 7.52 – 7.44 (m, 6H), 7.41 – 7.33 (m, 3H), 7.33 – 7.23 (m, 3H), 7.10 (td, *J* = 7.4, 1.3 Hz, 1H), 7.00 (td, *J* = 7.7, 1.3 Hz, 1H), 6.37 (s, 1H), 4.20 – 4.04 (m, 2H), 3.03 (t, *J* = 6.3 Hz, 2H). ^{13}C NMR (101 MHz, CD_3CN) δ 137.4, 133.6, 133.2, 132.3, 129.8, 128.7, 128.6, 128.6, 128.5, 128.0, 127.1, 126.3, 125.8, 125.6, 123.9, 122.6, 110.8, 42.3, 29.8. HRMS m/z (EI, $[M+H]^+$): $C_{24}H_{20}N$ calcd 322.1596, found 322.1576.

3-phenyl-1-(p-tolyl)-5,6-dihydropyrrolo[2,1-a]isoquinoline (3b).

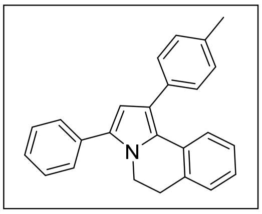

Yellowish oil (yield 83%). 1H NMR (400 MHz, CD3CN) δ 7.52 – 7.43 (m, 4H), 7.39 – 7.33 (m, 3H), 7.27 (dd, J = 7.8, 1.1 Hz, 2H), 7.21 (d, J = 7.8 Hz, 2H), 7.09 (td, J = 7.4, 1.3 Hz, 1H), 7.00 (td, J = 7.5, 1.1 Hz, 1H), 6.34 (s, 1H), 4.16 – 4.06 (m, 2H), 3.02 (t, J = 6.4 Hz, 2H), 2.37 (s, 3H). 13C NMR (101 MHz, CD3CN) δ 136.5, 135.0, 134.0, 133.6, 132.9, 130.4, 129.7, 129.2, 129.1, 129.0, 128.5, 127.6, 126.8, 126.3, 126.0, 124.4, 123.1, 111.4, 42.8, 30.4, 20.7. HRMS m/z (EI, $[M]^+$): $C_{25}H_{21}N$ calcd 335.1674, found 335.1666.

1-(4-methoxyphenyl)-3-phenyl-5,6-dihydropyrrolo[2,1-a]isoquinoline (3c).

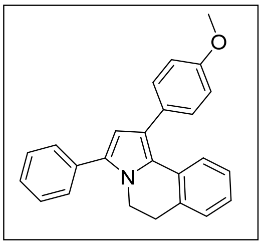

Yellowish oil (yield 80%). ^{1}H NMR (400 MHz, CD_3CN) δ 7.51 – 7.43 (m, 4H), 7.40 – 7.33 (m, 3H), 7.29 – 7.24 (m, 2H), 7.09 (td, *J* = 7.4, 1.3 Hz, 1H), 7.01 (td, *J* = 7.6, 1.1 Hz, 1H), 6.97 – 6.92 (m, 2H), 6.32 (s, 1H), 4.14 – 4.08 (m, 2H), 3.82 (s, 3H), 3.02 (t, *J* = 6.3 Hz, 2H). ^{13}C NMR (101 MHz, CD_3CN) δ 158.4, 133.4, 133.1, 132.4, 130.0, 129.9, 129.7, 128.6, 128.5, 127.9, 127.0, 126.3, 125.7, 125.4, 123.7, 122.3, 113.9, 110.8, 54.9, 42.3, 29.9. HRMS m/z (EI, $[M]^+$): $C_{25}H_{21}NO$ calcd 351.1623, found 351.1609.

1-(4-fluorophenyl)-3-phenyl-5,6-dihydropyrrolo[2,1-a]isoquinoline (3d).

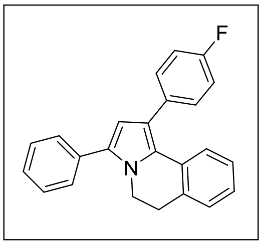

Yellowish oil (yield 90%). ^{1}H NMR (400 MHz, CD_3CN) δ 7.53 – 7.43 (m, 6H), 7.39 – 7.33 (m, 1H), 7.28 (d, *J* = 7.5 Hz, 1H), 7.22 (d, *J* = 7.7 Hz, 1H), 7.17 – 7.08 (m, 3H), 7.02 (t, *J* = 7.5 Hz, 1H), 6.35 (s, 1H), 4.23 – 4.01 (m, 2H), 3.02 (t, *J* = 6.4 Hz, 2H). ^{13}C NMR (101 MHz, CD_3CN) δ 161.6 (d, *J* = 242.4 Hz), 133.8, 133.7, 133.5, 133.2, 130.52 (d, *J* = 8.1 Hz), 129.7, 128.6, 128.5, 128.0, 127.2, 126.4, 125.9, 125.7, 123.8, 121.4, 115.2 (d, *J* = 21.2 Hz), 110.7, 42.3, 29.8. ^{19}F NMR (376 MHz, CD_3CN) δ -118.38 (tt, *J* = 9.1, 5.6 Hz). HRMS m/z (EI, $[M]^+$): $C_{24}H_{18}FN$ calcd 339.1415, found 339.1423.

1-(3-fluorophenyl)-3-phenyl-5,6-dihydropyrrolo[2,1-a]isoquinoline (3e).

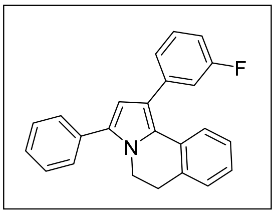

Yellowish oil (yield 81%). ^{1}H NMR (400 MHz, CD_3CN) δ 7.52 – 7.44 (m, 4H), 7.42 – 7.34 (m, 2H), 7.32 – 7.26 (m, 3H), 7.22 (ddd, *J* = 10.5, 2.5, 1.5 Hz, 1H), 7.13 (td, *J* = 7.4, 1.3 Hz, 1H), 7.07 – 7.00 (m, 2H), 6.40 (s, 1H), 4.18 – 4.05 (m, 2H), 3.03 (t, *J* = 6.3 Hz, 2H). ^{13}C NMR (101 MHz, CD_3CN) δ 167.7 (d, *J* = 299 Hz), 139.94, 133.73, 133.39, 132.17, 130.33, 130.25, 129.45, 128.61, 128.53, 128.01, 127.23, 126.44, 126.12, 124.67 (d, *J* = 3 Hz), 124.03, 121.13, 115.17(d, *J* = 21 Hz), 112.80 (d, *J* = 21 Hz), 110.63, 42.32, 29.80. ^{19}F NMR (376 MHz, CD_3CN) δ -115.23 (ddd, *J* = 10.6, 9.1, 6.2 Hz). HRMS m/z (EI, $[M]^+$): $C_{24}H_{18}FN$ calcd 339.1415, found 339.1418.

3-phenyl-1-(4-(trifluoromethyl)phenyl)-5,6-dihydropyrrolo[2,1-a]isoquinoline (3f).

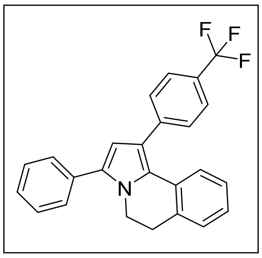

Yellowish solid (yield 69%). ^{1}H NMR (400 MHz, $CDCl_3$) δ 7.63 (q, *J* = 8.4 Hz, 4H), 7.47 – 7.42 (m, 4H), 7.35 (ddd, *J* = 15.9, 6.8, 1.9 Hz, 2H), 7.25 – 7.22 (m, 1H), 7.12 (td, *J* = 7.4, 1.4 Hz, 1H), 7.06 (td, *J* = 7.6, 1.4 Hz, 1H), 6.38 (s, 1H), 4.16 – 4.10 (m, 2H), 3.05 (t, *J* = 6.3 Hz, 2H). ^{13}C NMR (101 MHz, $CDCl_3$) δ 141.14, 133.97, 132.71, 132.14, 130.95, 129.54, 128.98, 128.77, 128.57, 127.88, 127.30, 126.75, 126.27, 126.10, 125.47, 125.44, 125.40, 125.36, 124.49, 123.16, 121.19, 110.81, 42.44, 30.34. ^{19}F NMR (376 MHz, CD_3CN) δ -62.71. HRMS m/z (EI, $[M]^+$): $C_{25}H_{18}F_3N$ calcd 389.1391, found 389.1383.

3-phenyl-1-(3-(trifluoromethyl)phenyl)-5,6-dihydropyrrolo[2,1-a]isoquinoline (3g).

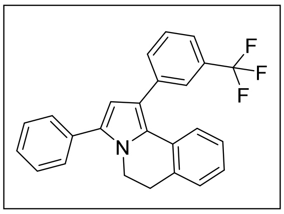

Yellowish solid (yield 70%). ^{1}H NMR (400 MHz, CD_3CN) δ 7.79 (s, 1H), 7.75 (d, *J* = 7.5 Hz, 1H), 7.63 – 7.53 (m, 2H), 7.52 – 7.45 (m, 4H), 7.38 (ddt, *J* = 7.5, 6.1, 1.9 Hz, 1H), 7.33 – 7.29 (m, 1H), 7.25 – 7.20 (m, 1H), 7.14 (td, *J* = 7.5, 1.3 Hz, 1H), 7.03 (td, *J* = 7.7, 1.3 Hz, 1H), 6.45 (s, 1H), 4.16 – 4.08 (m, 2H), 3.04 (t, *J* = 6.4 Hz, 2H). ^{13}C NMR (101 MHz, CD_3CN) δ 138.41, 133.93, 133.53, 132.53 – 132.27 (m), 132.11, 130.15 (q, *J* = 31.3 Hz), 129.41, 129.36, 128.63, 128.54, 128.10, 127.28, 126.42, 126.26, 125.17 (q, *J* = 3.9 Hz), 123.89, 122.74 (q, *J* = 3.9 Hz), 122.38 (q, *J* = 228.3 Hz) 120.76, 118.99, 110.57, 42.33, 29.81. ^{19}F NMR (376 MHz, CD_3CN) δ -63.14. HRMS m/z (EI, $[M]^+$): $C_{25}H_{18}F_3N$ calcd 389.1391, found 389.1386.

1-(3,4-difluorophenyl)-3-phenyl-5,6-dihydropyrrolo[2,1-a]isoquinoline (3h).

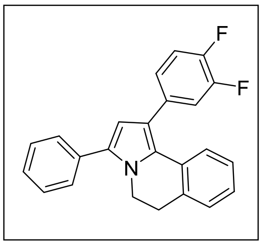

Yellowish oil (yield 65%). ^{1}H NMR (400 MHz, CD_3CN) δ 7.52 – 7.44 (m, 4H), 7.40 – 7.32 (m, 2H), 7.31 – 7.22 (m, 4H), 7.13 (td, *J* = 7.4, 1.2 Hz, 1H), 7.05 (td, *J* = 7.6, 1.2 Hz, 1H), 6.37 (s, 1H), 4.18 – 4.01 (m, 2H), 3.02 (t, *J* = 6.3 Hz, 2H). ^{13}C NMR (101 MHz, CD_3CN) δ 150.67 (d, *J* = 246.4 Hz), 149.41 (d, *J* = 232.3 Hz), 134.23, 133.93, 132.83 (d, *J* = 3 Hz), 132.63, 131.91 (d, *J* = 3 Hz), 129.99, 129.87, 129.14, 129.05, 128.56, 127.78, 127.03, 126.67, 126.51, 125.71 (m), 124.46, 120.79,

111.09, 42.83, 30.31. ^{19}F NMR (376 MHz, Acetonitrile-d_3) δ -140.33 – -140.48 (m), -143.72 – -143.86 (m). HRMS m/z (EI, [M]+): $C_{24}H_{17}F_2N$ calcd 357.1329, found 357.1328.

3-phenyl-1-(thiophen-2-yl)-5,6-dihydropyrrolo[2,1-a]isoquinoline (3i).

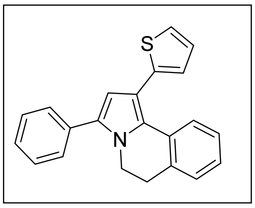

Yellowish oily solid (yield 78%). ^{1}H NMR (400 MHz, CD_3CN) δ 7.54 – 7.44 (m, 5H), 7.40 (tt, J = 5.9, 2.2 Hz, 2H), 7.31 (d, J = 7.0 Hz, 1H), 7.19 – 7.06 (m, 4H), 6.40 (s, 1H), 4.19 – 4.10 (m, 2H), 3.04 (t, J = 6.4 Hz, 2H). ^{13}C NMR (101 MHz, CD3CN) δ 139.1, 133.6, 133.2, 132.0, 129.4, 128.6, 128.0, 127.7, 127.6, 127.3, 126.7, 126.5, 126.2, 125.5, 124.7, 123.8, 114.3, 111.6, 42.3, 29.7. HRMS m/z (EI, [M]+): $C_{22}H_{17}NS$ calcd 327.1082, found 327.1077.

3-phenyl-1-(1H-pyrrol-2-yl)-5,6-dihydropyrrolo[2,1-a]isoquinoline (3j).

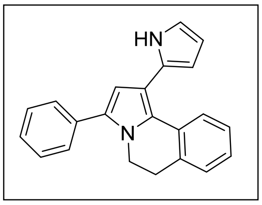

Yellowish solid (yield 85%). ^{1}H NMR (400 MHz, CD_3CN) δ 9.14 (s, 1H), 7.52 – 7.43 (m, 4H), 7.42 – 7.33 (m, 2H), 7.28 – 7.26 (m, 1H), 7.14 – 7.07 (m, 2H), 6.77 (td, *J* = 2.6, 1.7 Hz, 1H), 6.33 (s, 1H), 6.19 – 6.14 (m, 2H), 4.15 – 4.08 (m, 2H), 3.00 (t, *J* = 6.4 Hz, 2H). ^{13}C NMR (101 MHz, CD_3CN) δ 145.67, 134.32, 133.74, 133.26, 130.93, 129.53, 129.44, 128.79, 128.05, 127.95, 127.55, 126.98, 126.71, 124.32, 115.31, 111.38, 109.42, 107.31, 43.29, 30.69. HRMS m/z (EI, [M+H]+): $C_{22}H_{19}N_2$ calcd 311.1548, found 311.1537.

1-(furan-2-yl)-3-phenyl-5,6-dihydropyrrolo[2,1-a]isoquinoline (3k).

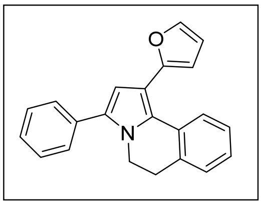

Yellowish oil (yield 82%). ^{1}H NMR (400 MHz, CD_3CN) δ 7.56 – 7.46 (m, 6H), 7.44 – 7.37 (m, 1H), 7.35 – 7.29 (m, 1H), 7.23 – 7.17 (m, 2H), 6.56 (dd, *J* = 3.2, 1.9 Hz, 1H), 6.50 (dd, *J* = 3.2, 0.7 Hz, 1H), 6.48 (s, 1H), 4.16 – 4.11 (m, 2H), 3.03 (t, *J* = 6.4 Hz, 2H). ^{13}C NMR (101 MHz, CD_3CN) δ 150.8, 141.0, 133.8, 133.2, 132.1, 129.4, 128.6, 128.5, 127.8, 127.3, 126.7, 126.3, 124.2, 111.3, 109.7, 106.2, 42.3, 29.6. HRMS m/z (EI, [M]+): $C_{22}H_{17}NO$ calcd 311.1310, found 311.1309.

3-methyl-1-phenyl-5,6-dihydropyrrolo[2,1-a]isoquinoline (3l).

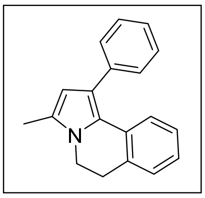

Yellowish oil (yield 50%). ^{1}H NMR (400 MHz, Acetonitrile-d_3) δ 7.43 – 7.38 (m, 2H), 7.34 (t, J = 7.5 Hz, 2H), 7.28 – 7.18 (m, 3H), 7.03 (td, J = 7.4, 1.1 Hz, 1H), 6.98 – 6.92 (m, 1H), 5.93 (s, 1H), 3.98 – 3.80 (m, 2H), 3.03 (t, J = 6.4 Hz, 2H), 2.26 (s, 3H). ^{13}C NMR (101 MHz, CD_3CN) δ 138.0, 132.1, 130.0, 128.6, 128.5, 128.1, 127.0, 126.3, 126.0, 125.2, 123.4, 123.1, 121.7, 109.1, 40.7, 29.5, 10.9. HRMS m/z (EI, $[M]^+$): $C_{19}H_{17}N$ calcd 259.1361, found 259.1333.

1-methyl-3-phenyl-5,6-dihydropyrrolo[2,1-a]isoquinoline (3m).

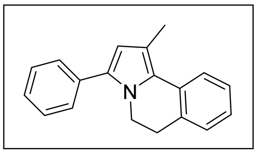

Yellowish oil (yield 15%). ^{1}H NMR (400 MHz, Acetonitrile-d_3) δ 7.63 (d, J = 7.5 Hz, 1H), 7.46 – 7.40 (m, 4H), 7.34 – 7.25 (m, 3H), 7.12 (td, J = 7.4, 1.2 Hz, 1H), 6.16 (s, 1H), 4.14 – 4.05 (m, 2H), 2.95 (t, J = 6.4 Hz, 2H), 2.38 (s, 3H). HRMS m/z (EI, $[M]^+$): $C_{19}H_{17}N$ calcd 259.1361, found 259.1358.

8-fluoro-1,3-diphenyl-5,6-dihydropyrrolo[2,1-a]isoquinoline (3n).

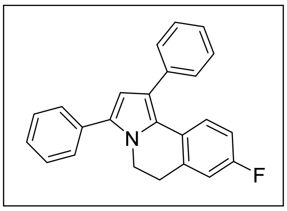

White solid (yield 63%). ^{1}H NMR (400 MHz, Acetonitrile-d_3) δ 7.51 – 7.44 (m, 6H), 7.41 – 7.36 (m, 3H), 7.33 – 7.30 (m, 1H), 7.24 (dd, J = 8.7, 5.7 Hz, 1H), 7.07 (dd, J = 9.4, 2.7 Hz, 1H), 6.77 (td, J = 8.9, 2.7 Hz, 1H), 6.36 (s, 1H), 4.15 – 4.08 (m, 2H), 3.03 (t, J = 6.4 Hz, 2H). ^{13}C NMR (101 Hz, CD_3CN) δ 160.6 (d, J = 278 Hz), 137.3, 135.8 (d, J = 7.1 Hz), 133.5, 132.3, 130.3, 130.1, 128.7, 128.6, 128.6, 128.5, 127.2, 126.4, 125.6 (d, J = 8.1 Hz), 122.2, 114.6 (d, J = 22.2 Hz), 113.1 (d, J = 22.2 Hz), 110.7, 42.0, 29.8. HRMS m/z (EI, $[M]^+$): $C_{24}H_{18}FN$ calcd 339.1423, found 339.1426.

1,3-diphenyl-8-(trifluoromethyl)-5,6-dihydropyrrolo[2,1-a]isoquinoline (3o).

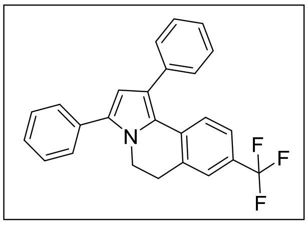

Yellowish solid (yield 81%). ^{1}H NMR (400 MHz, Acetonitrile-d_3) δ 7.60 (s, 1H), 7.53 – 7.45 (m, 6H), 7.43 – 7.27 (m, 6H), 6.41 (s, 1H), 4.22 – 4.09 (m, 2H), 3.10 (t, J = 6.4 Hz, 2H). ^{13}C NMR (101

MHz, CD_3CN) δ 136.9, 134.6, 133.8, 133.6, 132.0, 128.8, 128.7, 128.6, 128.6, 127.5, 126.7, 126.2 (q, J = 32.3 Hz), 124.7 (q, J = 4 Hz), 124.6 (q, J = 271.7 Hz), 124.5, 124.4, 123.9, 123.2 (q, J = 4 Hz), 111.3, 42.1, 29.6. HRMS m/z (EI, $[M]^+$): $C_{25}H_{18}F_3N$ calcd 389.1391, found 389.1394.

1-(2-hydroxy-3-methoxyphenyl)-3-phenyl-1,2,3,5,6,10b-hexahydropyrrolo[2,1-a]isoquinolin-3-ol (7p).

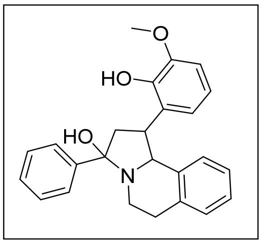

White solid. ^1H NMR (400 MHz, CD_3CN) δ 7.79 – 7.74 (m, 2H), 7.49 – 7.44 (m, 2H), 7.43 – 7.36 (m, 1H), 7.25 (d, J = 7.7 Hz, 1H), 7.15 (t, J = 7.2 Hz, 1H), 6.97 (t, J = 7.2 Hz, 1H), 6.84 (d, J = 7.5 Hz, 1H), 6.64 (dd, J = 8.1, 1.4 Hz, 1H), 6.42 (t, J = 7.8 Hz, 1H), 6.22 (dd, J = 7.6, 1.4 Hz, 1H), 5.09 (d, J = 3.8 Hz, 1H), 3.76 (s, 3H), 3.58 – 3.49 (m, 1H), 3.04 (dt, J = 13.3, 4.2 Hz, 1H), 2.88 – 2.77 (m, 1H), 2.62 (dd, J = 11.4, 3.7 Hz, 1H), 2.40 – 2.33 (m, 2H), 2.27 (d, J = 11.4 Hz, 1H). ^{13}C NMR (101 MHz, CD_3CN) δ 148.06, 142.87, 141.66, 136.37, 136.36, 128.58, 128.48, 128.27, 128.26, 127.96, 127.17, 126.99, 126.97, 125.76, 125.44, 121.36, 118.78, 110.77, 100.07, 67.36, 55.44, 44.61, 42.02, 39.80, 28.89. HRMS m/z (EI, $[M+H-H_2O]^+$): $C_{25}H_{24}NO_2$ calcd 370.1807, found 370.1805.

1-(2-hydroxy-3-methoxyphenyl)-3-phenyl-1,2,3,5,6,10b-hexahydropyrrolo[2,1-a]isoquinolin-10b-d-3-ol (7p-D_1).

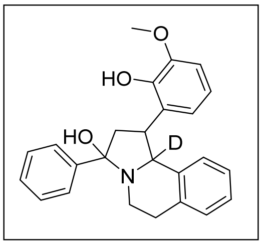

White solid. ^1H NMR (400 MHz, Acetonitrile-d_3) δ 7.77 (dd, J = 8.4, 1.3 Hz, 2H), 7.50 – 7.43 (m, 2H), 7.42 – 7.37 (m, 1H), 7.27 – 7.23 (m, 1H), 7.15 (td, J = 7.8, 1.2 Hz, 1H), 6.97 (td, J = 7.5, 1.3 Hz, 1H), 6.84 (d, J = 8.2 Hz, 1H), 6.64 (dd, J = 8.1, 1.4 Hz, 1H), 6.42 (t, J = 7.8 Hz, 1H), 6.25 – 6.18 (m, 1H), 3.76 (s, 3H), 3.52 (d, J = 3.7 Hz, 1H), 3.04 (dt, J = 13.3, 4.2 Hz, 1H), 2.89 – 2.76 (m, 1H), 2.62 (dd, J = 11.4, 3.7 Hz, 1H), 2.37 – 6.34 (m, 2H), 2.27 (dd, J = 11.4, 1.0 Hz, 1H). ^{13}C NMR (101 MHz, CD_3CN) δ 148.0, 142.9, 141.7, 136.4, 128.3, 128.3, 128.0, 127.2, 127.0, 127.0, 126.9, 125.8, 125.5, 121.3, 118.8, 110.7, 100.1, 67.4(t), 55.4, 44.5, 42.0, 39.8, 28.9.

^{1}H and ^{13}C NMR spectra of DHIQs (3a-o) and 7p.

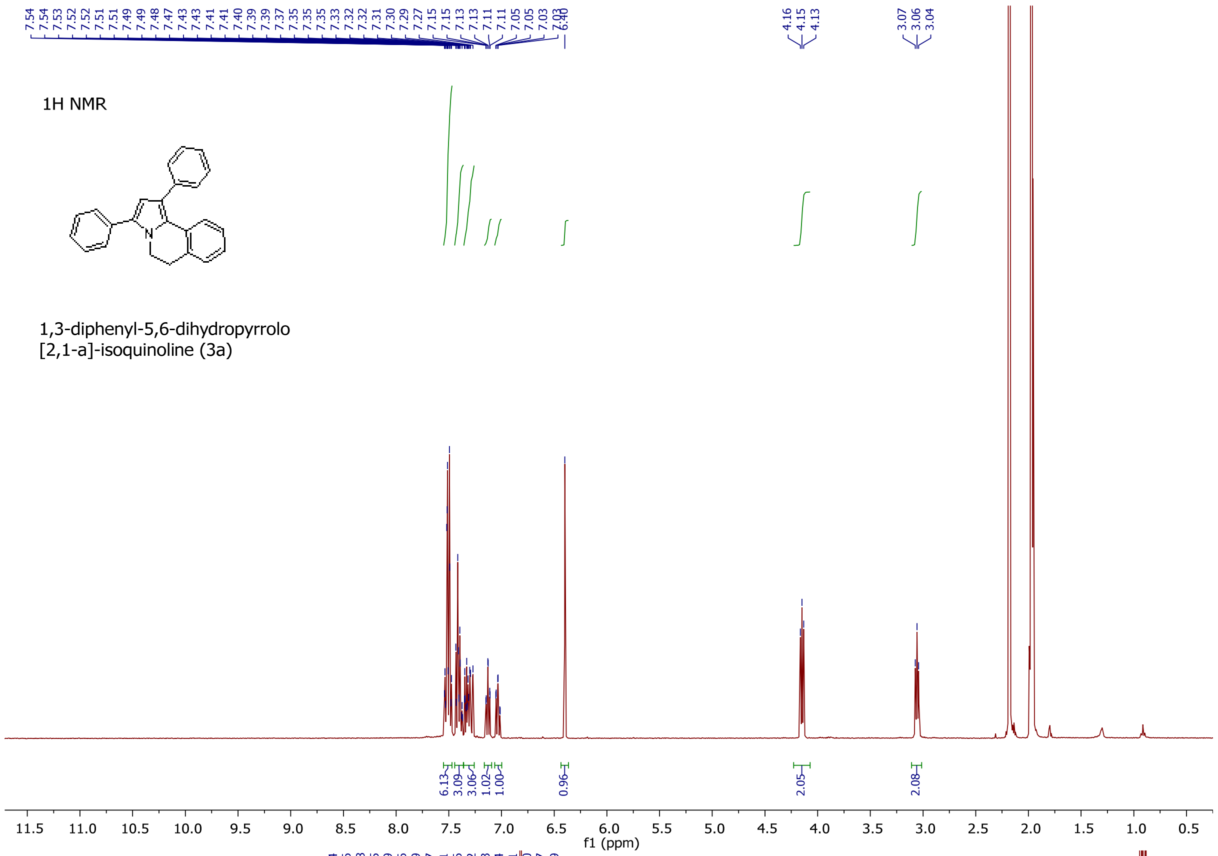

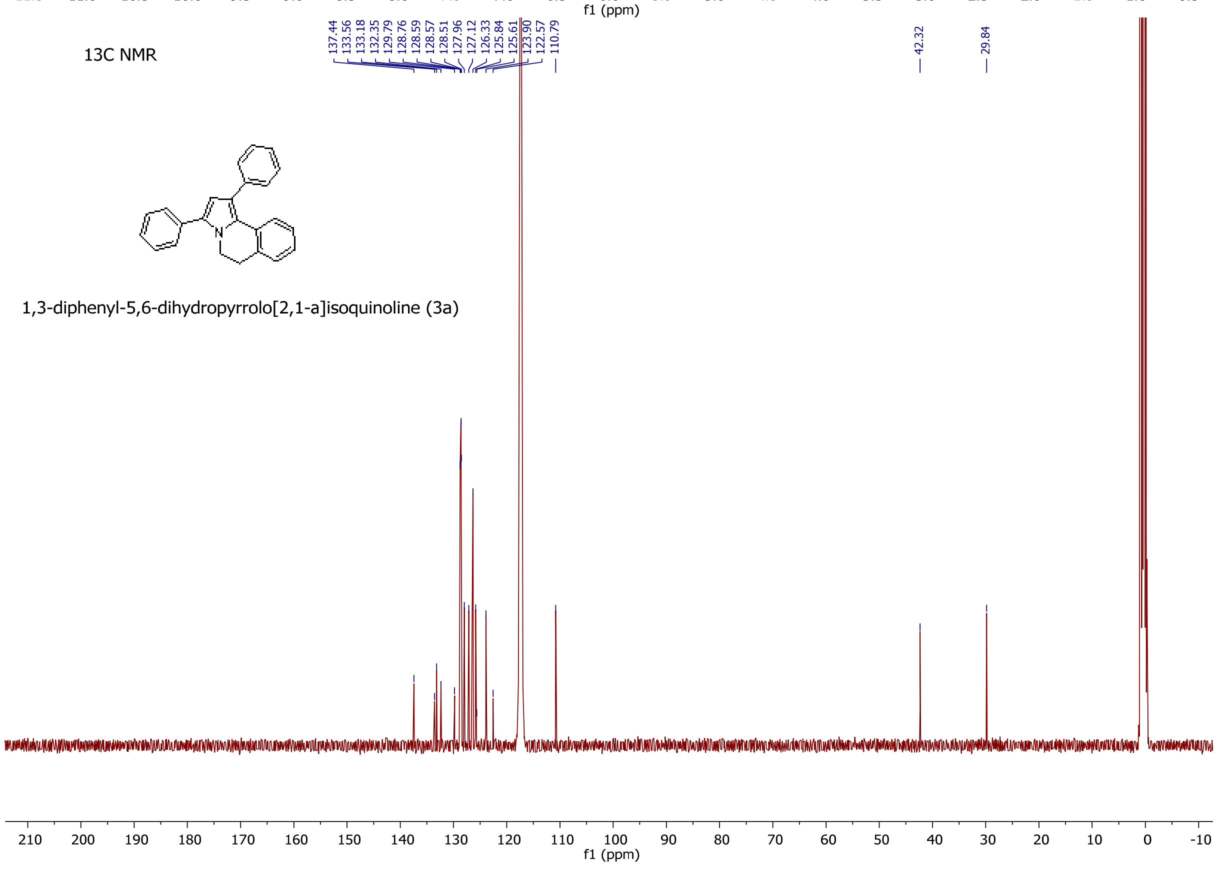

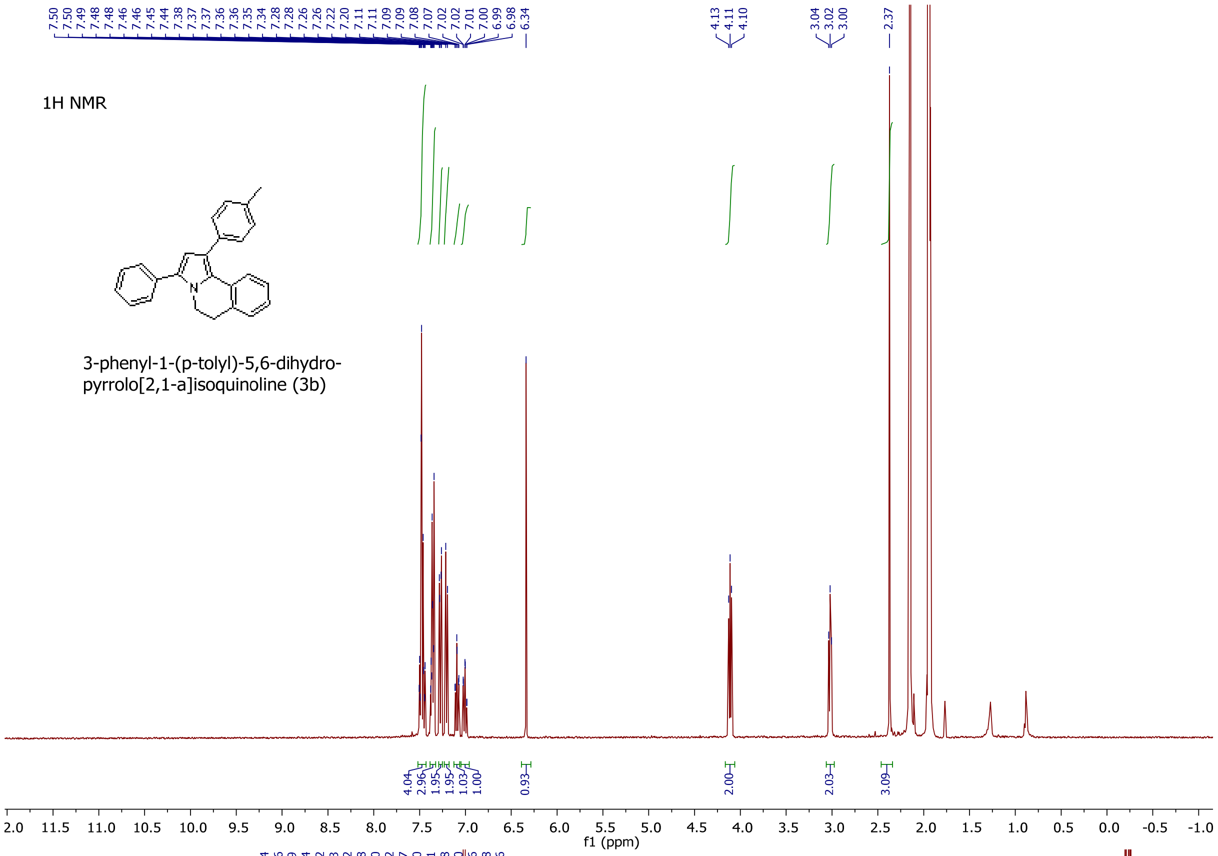

1H NMR
3-phenyl-1-(p-tolyl)-5,6-dihydro-pyrrolo[2,1-a]isoquinoline (3b)
7.50 7.50 7.49 7.48 7.48 7.46 7.45 7.44 7.38 7.37 7.37 7.36 7.36 7.35 7.34 7.28 7.28 7.26 7.26 7.22 7.20 7.11 7.11 7.09 7.09 7.08 7.07 7.02 7.02 7.01 7.00 6.99 6.98 6.34
4.13 4.11 4.10
3.04 3.02 3.00
2.37
4.04 2.96 1.95 1.95 1.03 1.00 0.93 2.00 2.03 3.09
f1 (ppm)

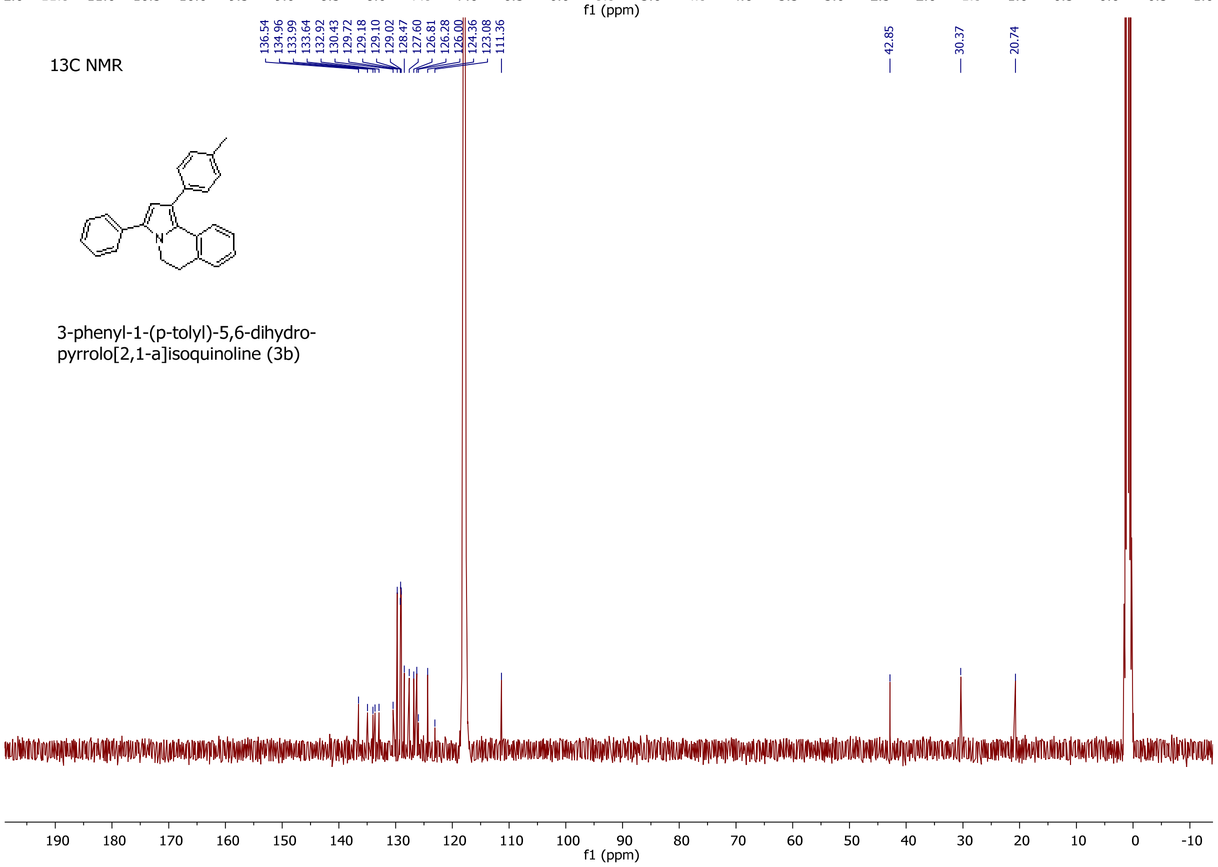

13C NMR
3-phenyl-1-(p-tolyl)-5,6-dihydro-pyrrolo[2,1-a]isoquinoline (3b)
136.54 134.96 133.99 133.64 132.92 130.43 129.72 129.18 129.10 129.02 128.47 127.60 126.81 126.28 126.00 124.36 123.08 111.36
42.85
30.37
20.74
f1 (ppm)

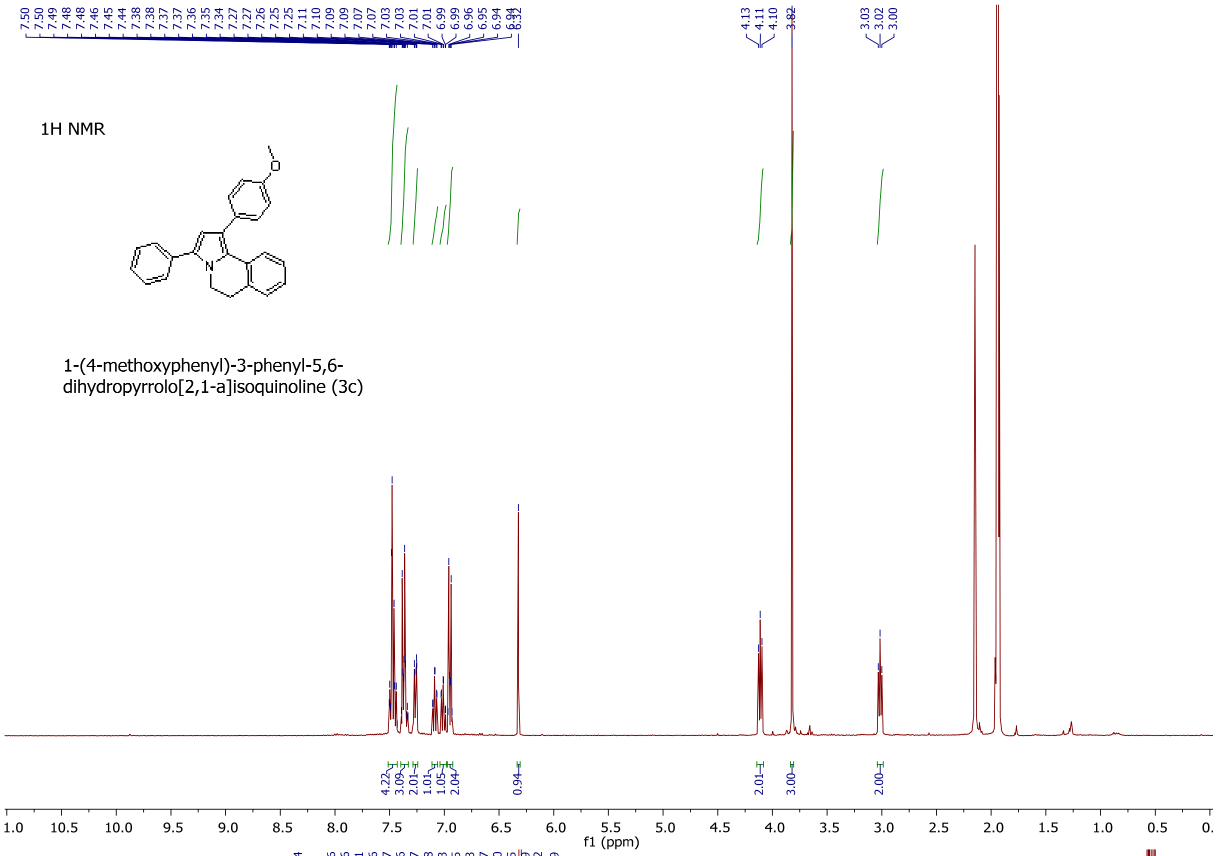
7.50 7.50 7.49 7.48 7.48 7.46 7.45 7.44 7.38 7.38 7.37 7.37 7.36 7.35 7.34 7.27 7.27 7.26 7.25 7.25 7.11 7.10 7.09 7.09 7.07 7.07 7.03 7.03 7.01 7.01 6.99 6.99 6.96 6.95 6.94 6.94 6.32
4.13 4.11 4.10 3.82
3.03 3.02 3.00
1H NMR
1-(4-methoxyphenyl)-3-phenyl-5,6-
dihydropyrrolo[2,1-a]isoquinoline (3c)
4.22 3.09 2.01 1.01 1.05 2.04 0.94 2.01 3.00 2.00
f1 (ppm)

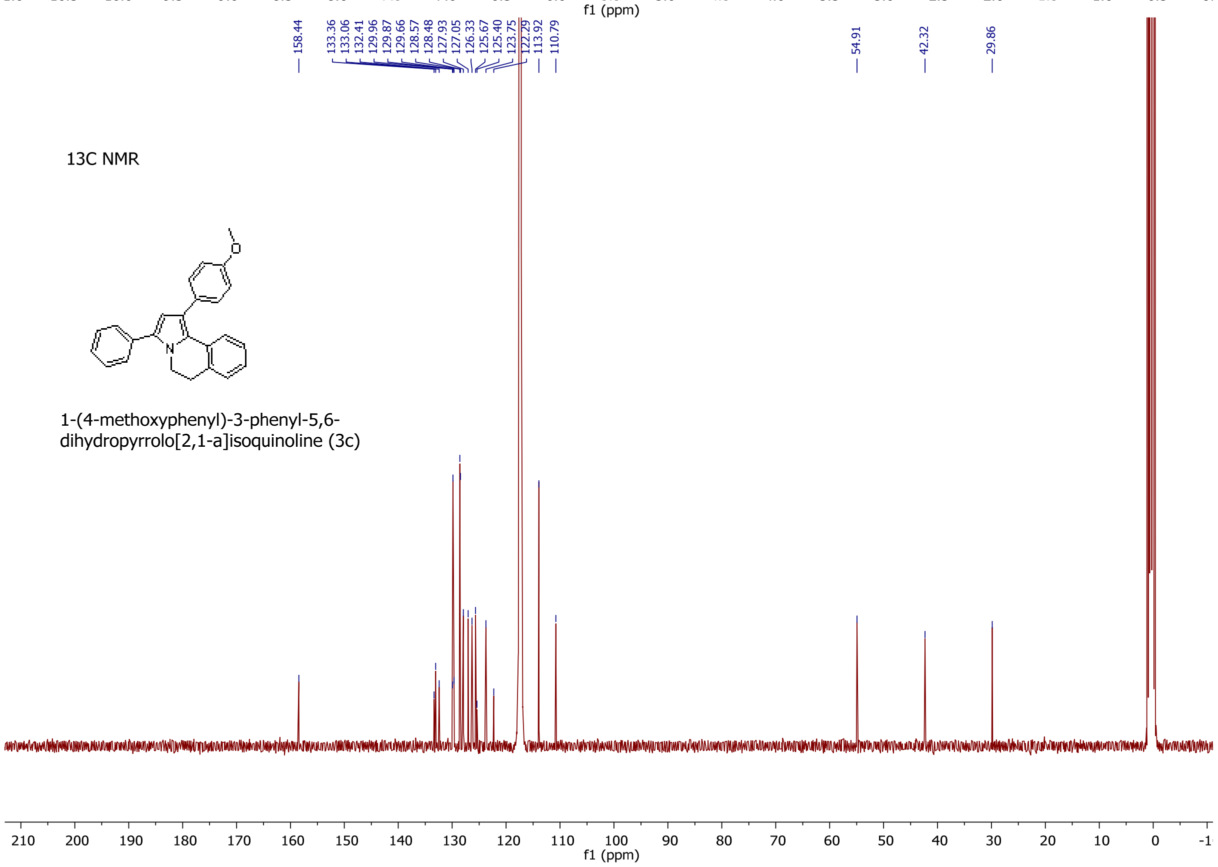
158.44
133.36 133.06 132.41 129.96 129.87 129.66 128.57 128.48 127.93 127.05 126.33 125.67 125.40 123.75 122.29 113.92 110.79
54.91 42.32 29.86
13C NMR
1-(4-methoxyphenyl)-3-phenyl-5,6-
dihydropyrrolo[2,1-a]isoquinoline (3c)
f1 (ppm)

1H NMR

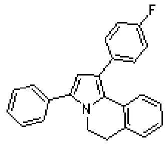

1-(4-fluorophenyl)-3-phenyl-5,6-dihydropyrrolo[2,1-a]isoquinoline (3d)

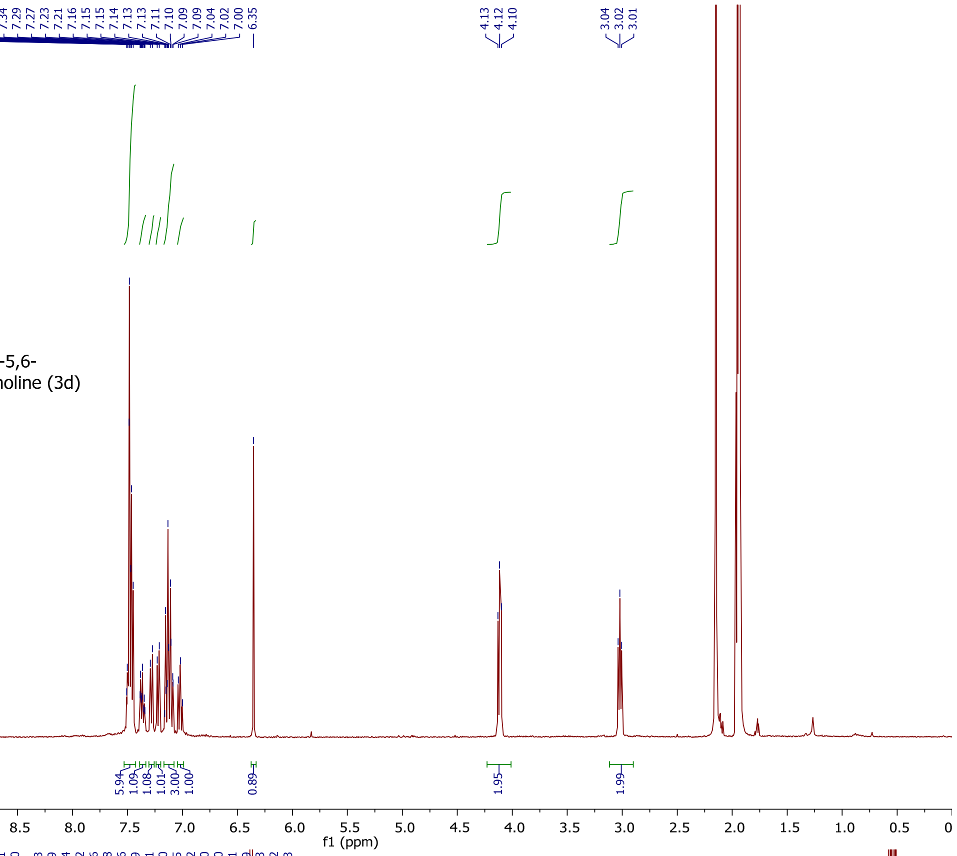

13C NMR

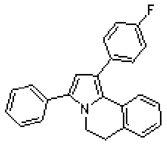

1-(4-fluorophenyl)-3-phenyl-5,6-dihydropyrrolo[2,1-a]isoquinoline (3d)

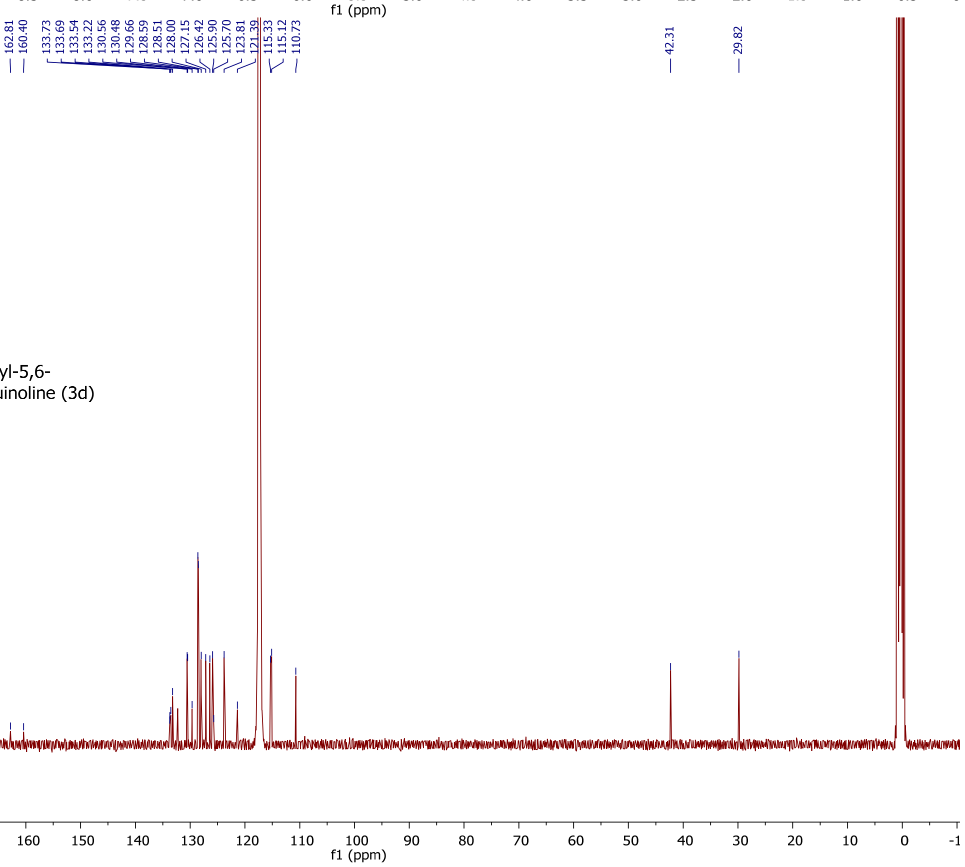

19F NMR

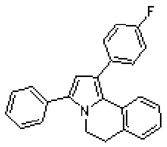

1-(4-fluorophenyl)-3-phenyl-5,6-dihydropyrrolo[2,1-a]isoquinoline (3d)

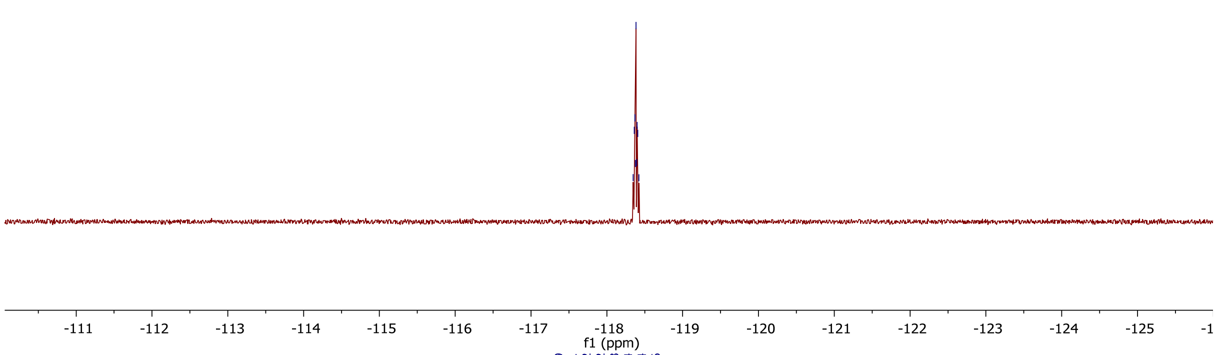

19F NMR

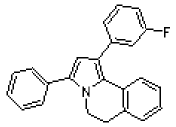

1-(3-fluorophenyl)-3-phenyl-5,6-dihydropyrrolo[2,1-a]isoquinoline (3e)

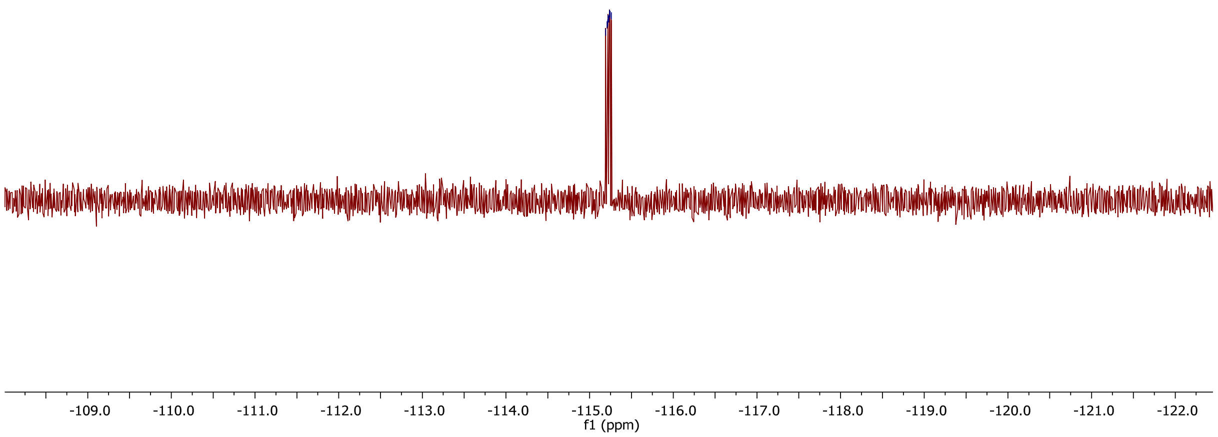

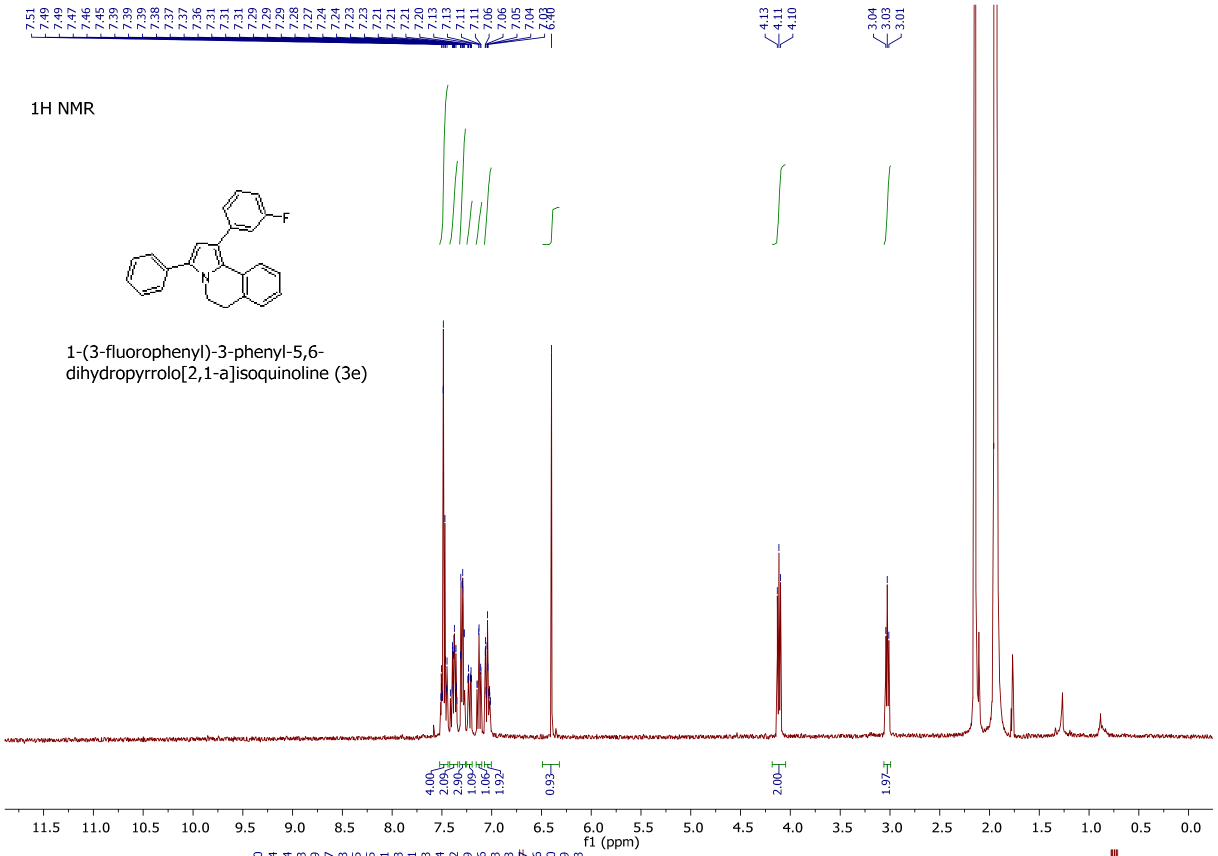
1H NMR
F
N
1-(3-fluorophenyl)-3-phenyl-5,6-
dihydropyrrolo[2,1-a]isoquinoline (3e)
7.51 7.49 7.49 7.47 7.46 7.45 7.39 7.39 7.39 7.38 7.37 7.37 7.36 7.31 7.31 7.31 7.31 7.29 7.29 7.29 7.28 7.27 7.24 7.24 7.23 7.23 7.21 7.21 7.21 7.20 7.13 7.13 7.11 7.11 7.06 7.06 7.05 7.04 7.03 6.40
4.13 4.11 4.10
3.04 3.03 3.01
4.00 2.09 2.90 1.09 1.06 1.92 0.93 2.00 1.97
f1 (ppm)

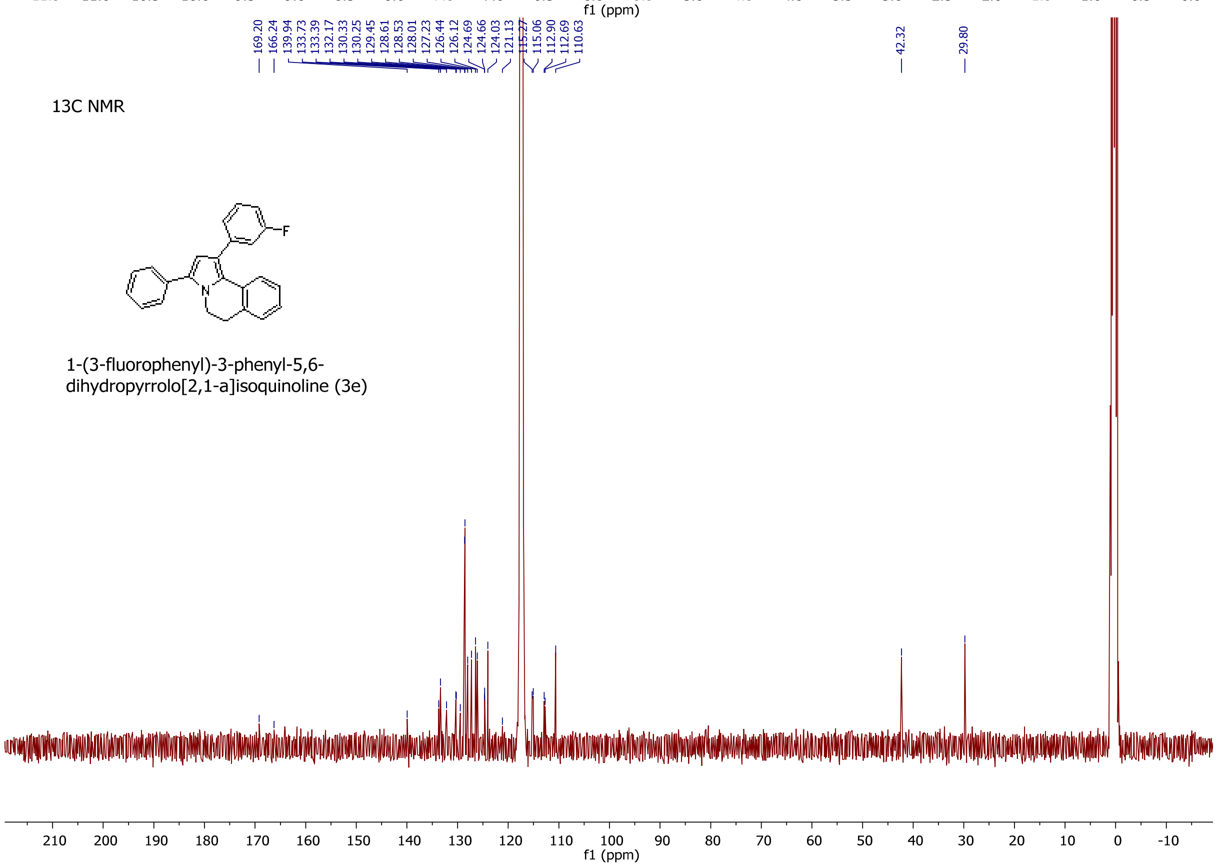
13C NMR
F
N
1-(3-fluorophenyl)-3-phenyl-5,6-
dihydropyrrolo[2,1-a]isoquinoline (3e)
169.20 166.24 139.94 133.73 133.39 132.17 130.33 130.25 129.45 128.61 128.53 128.01 127.23 126.44 126.12 124.69 124.66 124.03 121.13 115.27 115.06 112.90 112.69 110.63
42.32
29.80
f1 (ppm)

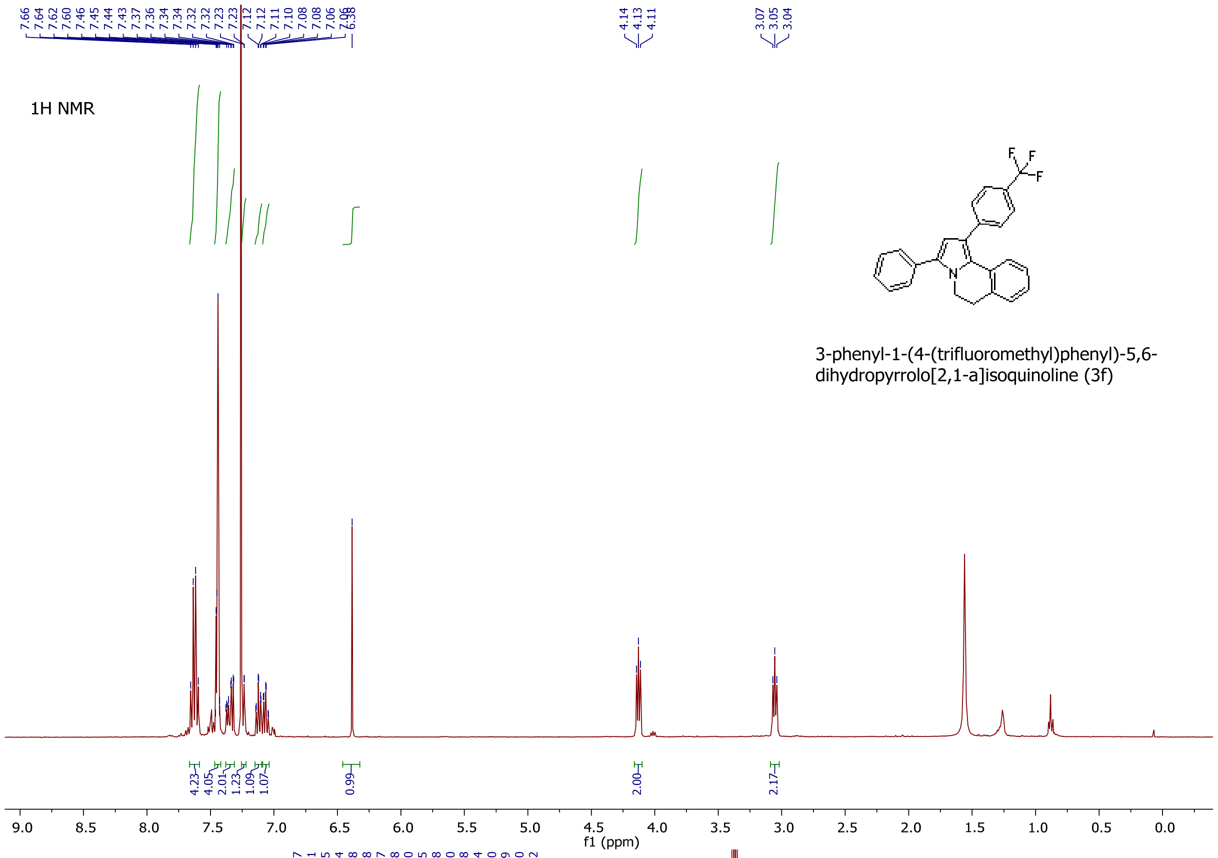

7.66 7.64 7.62 7.60 7.46 7.45 7.44 7.43 7.37 7.36 7.34 7.34 7.32 7.32 7.23 7.23 7.12 7.12 7.11 7.10 7.08 7.08 7.06 7.06 6.38
4.14 4.13 4.11
3.07 3.05 3.04
1H NMR
F
F
F
N
3-phenyl-1-(4-(trifluoromethyl)phenyl)-5,6-dihydropyrrolo[2,1-a]isoquinoline (3f)
4.23 4.05 2.01 1.23 1.09 1.07 0.99 2.00 2.17
9.0 8.5 8.0 7.5 7.0 6.5 6.0 5.5 5.0 4.5 4.0 3.5 3.0 2.5 2.0 1.5 1.0 0.5 0.0
f1 (ppm)

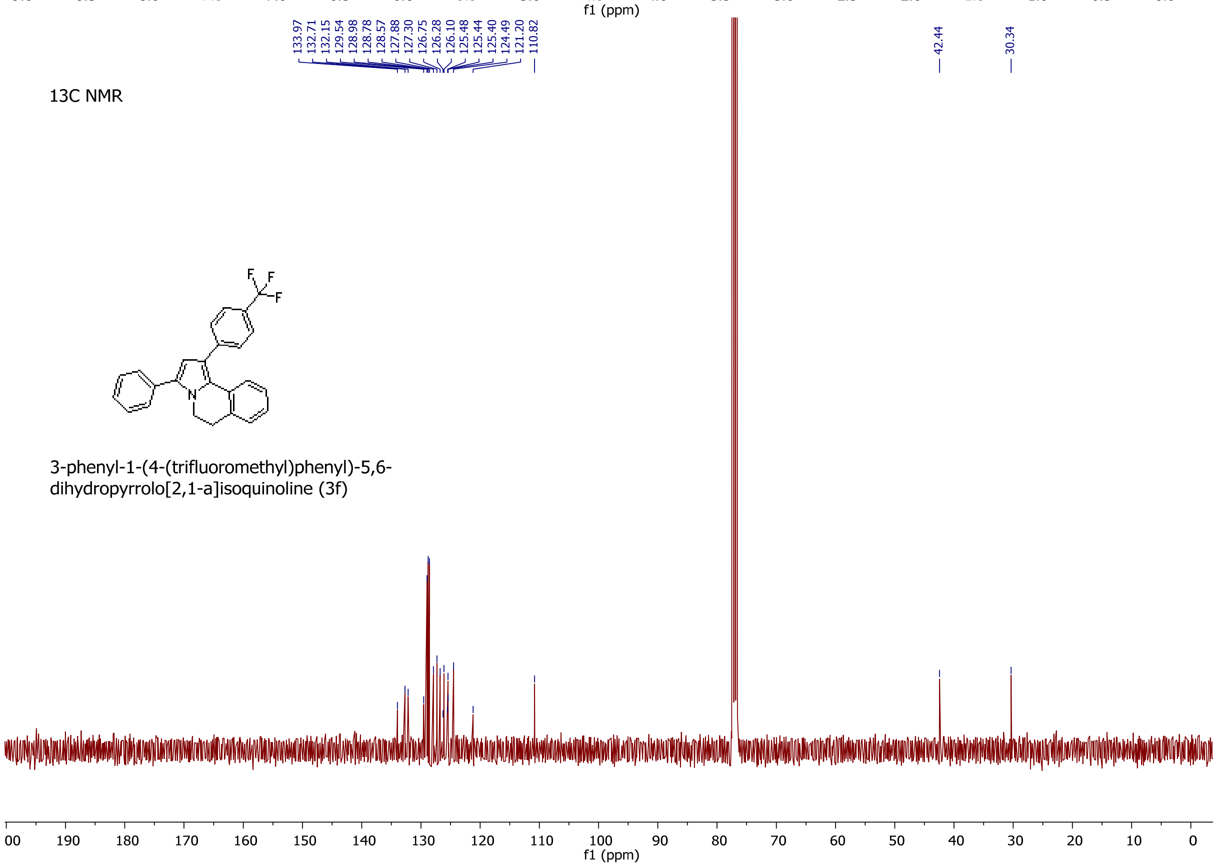

133.97 132.71 132.15 129.54 128.98 128.78 128.57 127.88 127.30 126.75 126.28 126.10 125.48 125.44 125.40 124.49 121.20 110.82
42.44
30.34
13C NMR
F
F
F
N
3-phenyl-1-(4-(trifluoromethyl)phenyl)-5,6-dihydropyrrolo[2,1-a]isoquinoline (3f)
00 190 180 170 160 150 140 130 120 110 100 90 80 70 60 50 40 30 20 10 0
f1 (ppm)

19F NMR

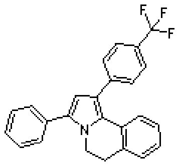

3-phenyl-1-(4-(trifluoromethyl)phenyl)-5,6-dihydropyrrolo[2,1-a]isoquinoline (3f)

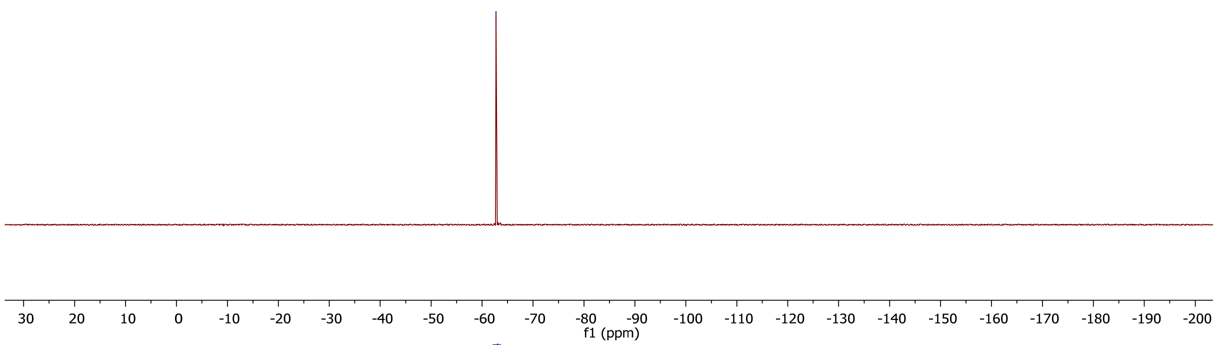

19F NMR

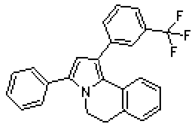

3-phenyl-1-(3-(trifluoromethyl)phenyl)-5,6-dihydropyrrolo[2,1-a]isoquinoline (3g)

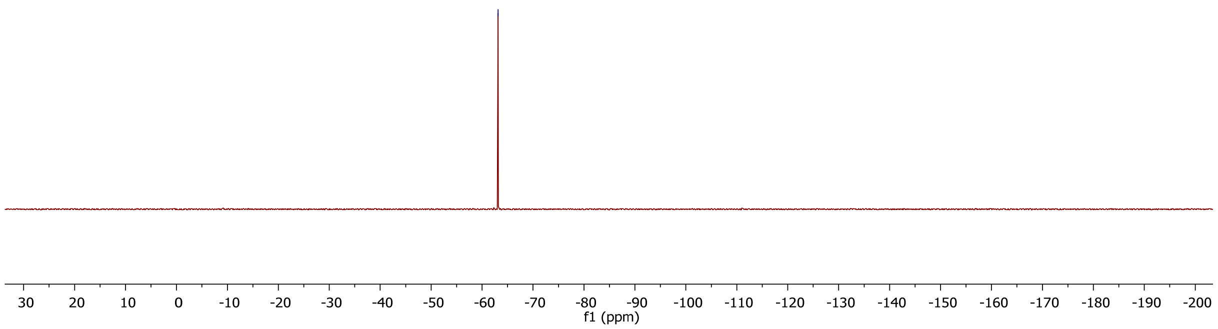

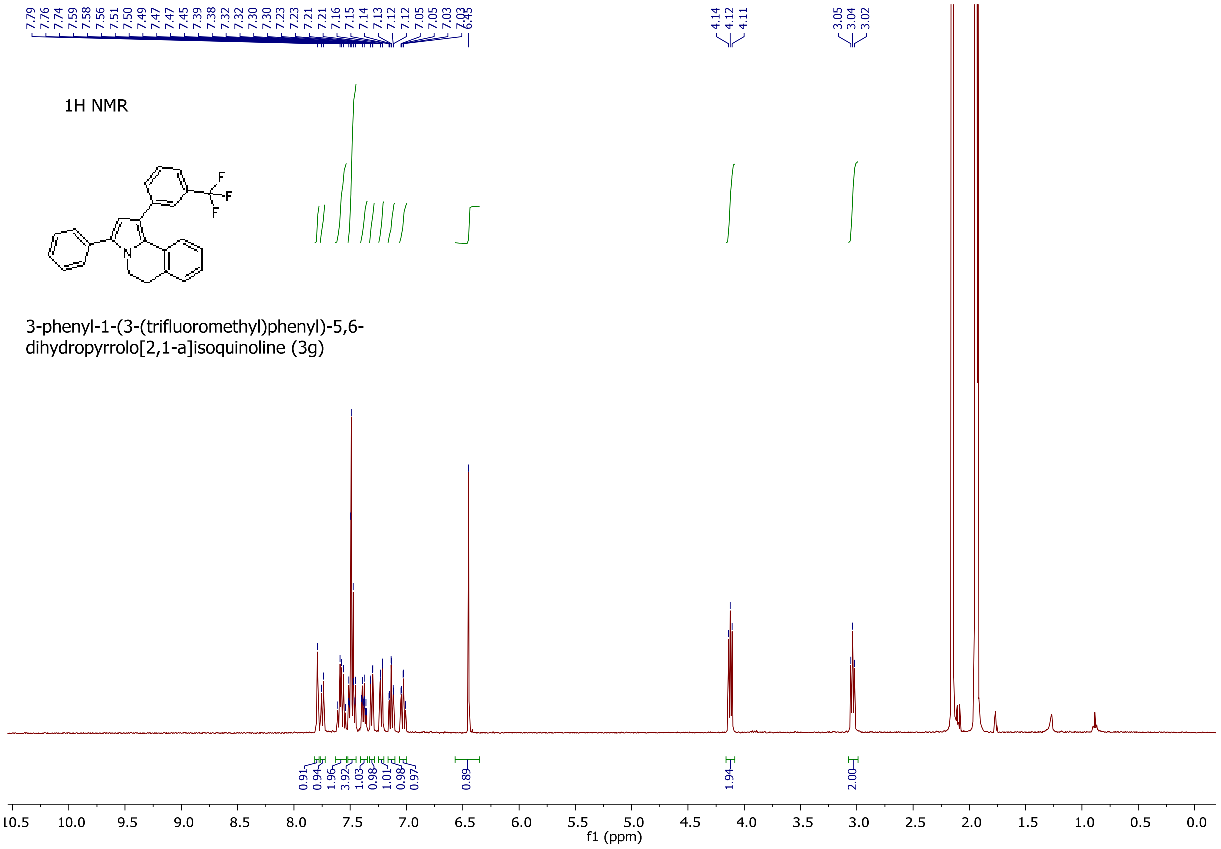
7.79 7.76 7.74 7.59 7.58 7.56 7.51 7.50 7.49 7.47 7.47 7.45 7.39 7.38 7.32 7.32 7.30 7.30 7.23 7.23 7.21 7.21 7.16 7.15 7.14 7.13 7.12 7.12 7.05 7.05 7.03 7.03 6.45
4.14 4.12 4.11
3.05 3.04 3.02
1H NMR
F
F
F
N
3-phenyl-1-(3-(trifluoromethyl)phenyl)-5,6-dihydropyrrolo[2,1-a]isoquinoline (3g)
0.91 0.94 1.96 3.92 1.03 0.98 1.01 0.98 0.97 0.89 1.94 2.00
10.5 10.0 9.5 9.0 8.5 8.0 7.5 7.0 6.5 6.0 5.5 5.0 4.5 4.0 3.5 3.0 2.5 2.0 1.5 1.0 0.5 0.0
f1 (ppm)

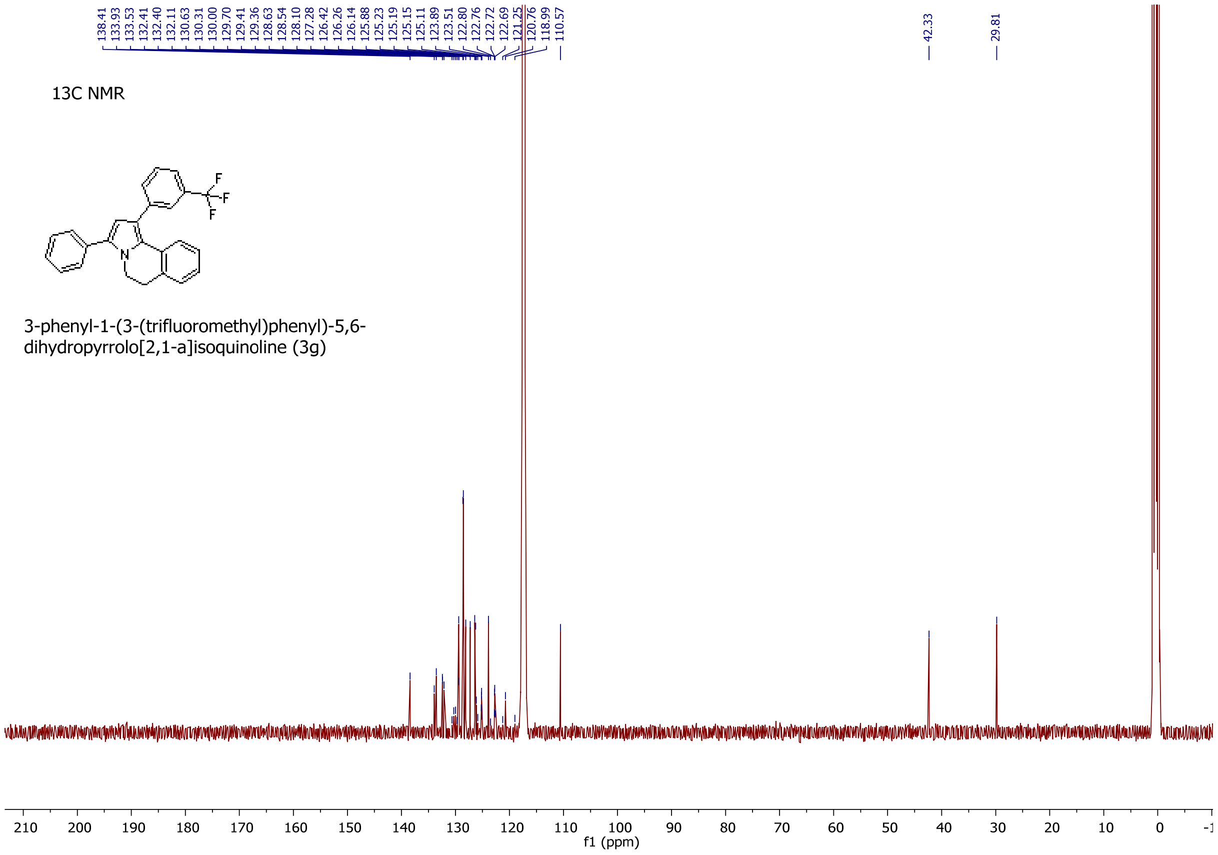
138.41 133.93 133.53 132.41 132.40 132.11 130.63 130.31 130.00 129.70 129.41 129.36 128.63 128.54 128.10 127.28 126.42 126.26 126.14 125.88 125.23 125.19 125.15 125.11 123.89 123.51 122.80 122.76 122.72 122.69 121.25 120.76 118.99 110.57
42.33
29.81
13C NMR
F
F
F
N
3-phenyl-1-(3-(trifluoromethyl)phenyl)-5,6-dihydropyrrolo[2,1-a]isoquinoline (3g)
210 200 190 180 170 160 150 140 130 120 110 100 90 80 70 60 50 40 30 20 10 0 -1
f1 (ppm)

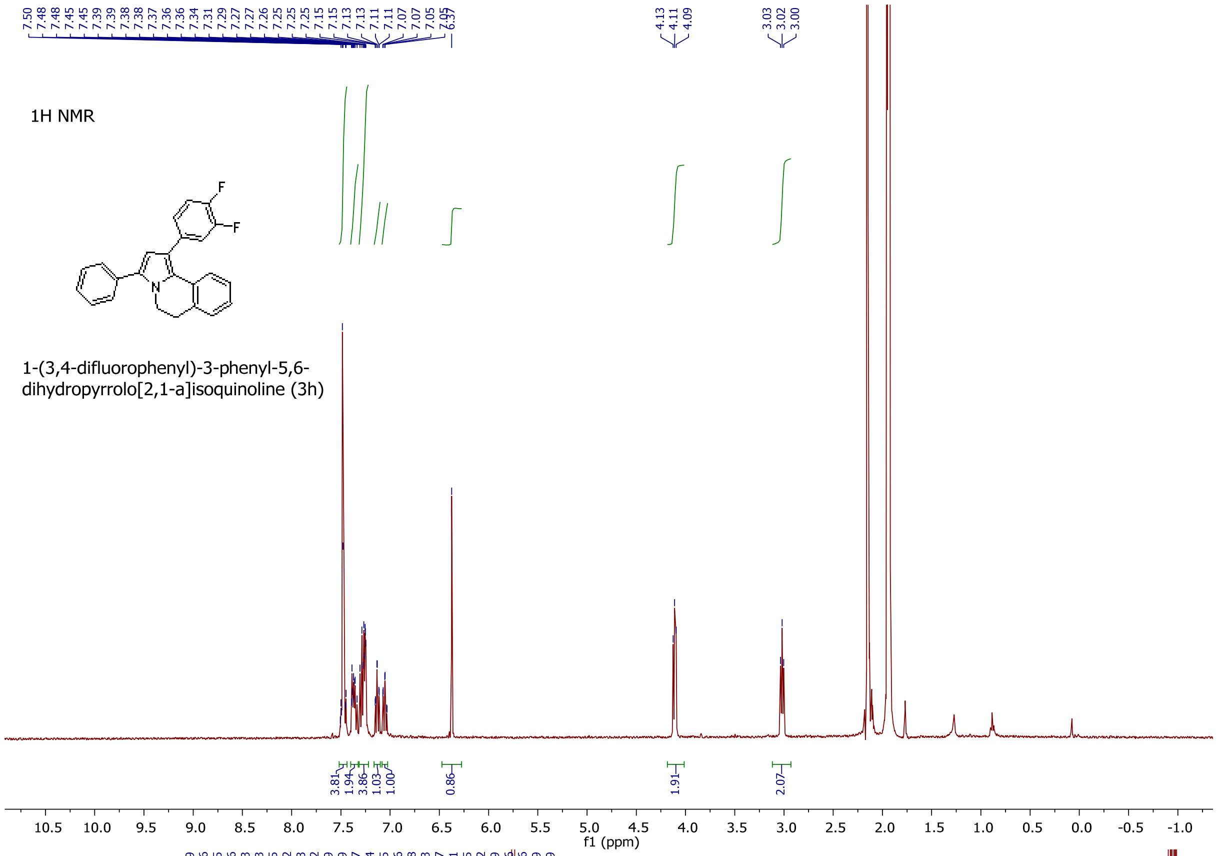
7.50 7.48 7.48 7.45 7.45 7.39 7.39 7.38 7.38 7.37 7.36 7.36 7.34 7.31 7.29 7.27 7.27 7.26 7.26 7.25 7.25 7.15 7.15 7.13 7.13 7.11 7.11 7.07 7.07 7.05 7.05 6.37
4.13 4.11 4.09
3.03 3.02 3.00
1H NMR
F
F
N
1-(3,4-difluorophenyl)-3-phenyl-5,6-
dihydropyrrolo[2,1-a]isoquinoline (3h)
3.81 1.94 3.86 1.03 1.00 0.86 1.91 2.07
10.5 10.0 9.5 9.0 8.5 8.0 7.5 7.0 6.5 6.0 5.5 5.0 4.5 4.0 3.5 3.0 2.5 2.0 1.5 1.0 0.5 0.0 -0.5 -1.0
f1 (ppm)

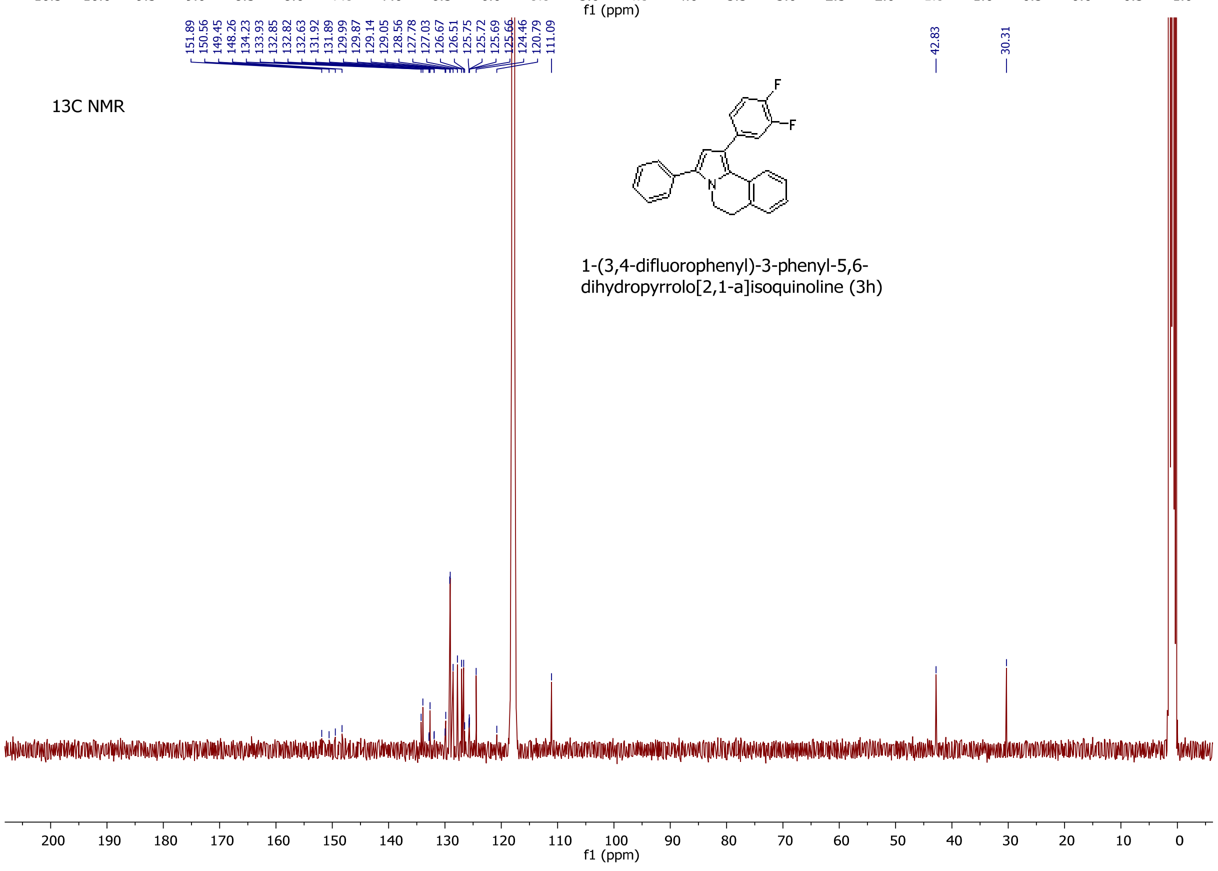
151.89 150.56 149.45 148.26 134.23 133.93 132.85 132.82 132.63 131.92 131.89 129.99 129.87 129.14 129.05 128.56 127.78 127.03 126.67 126.51 125.75 125.72 125.69 125.66 124.46 120.79 111.09
42.83
30.31
13C NMR
F
F
N
1-(3,4-difluorophenyl)-3-phenyl-5,6-
dihydropyrrolo[2,1-a]isoquinoline (3h)
200 190 180 170 160 150 140 130 120 110 100 90 80 70 60 50 40 30 20 10 0
f1 (ppm)

19F NMR

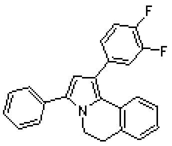

1-(3,4-difluorophenyl)-3-phenyl-5,6-dihydropyrrolo[2,1-a]isoquinoline (3h)

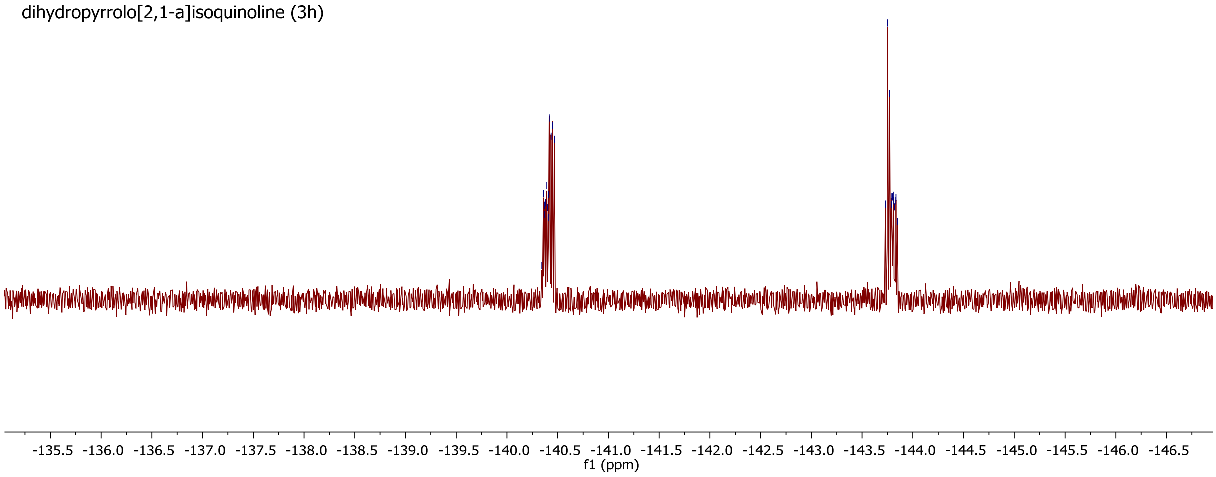

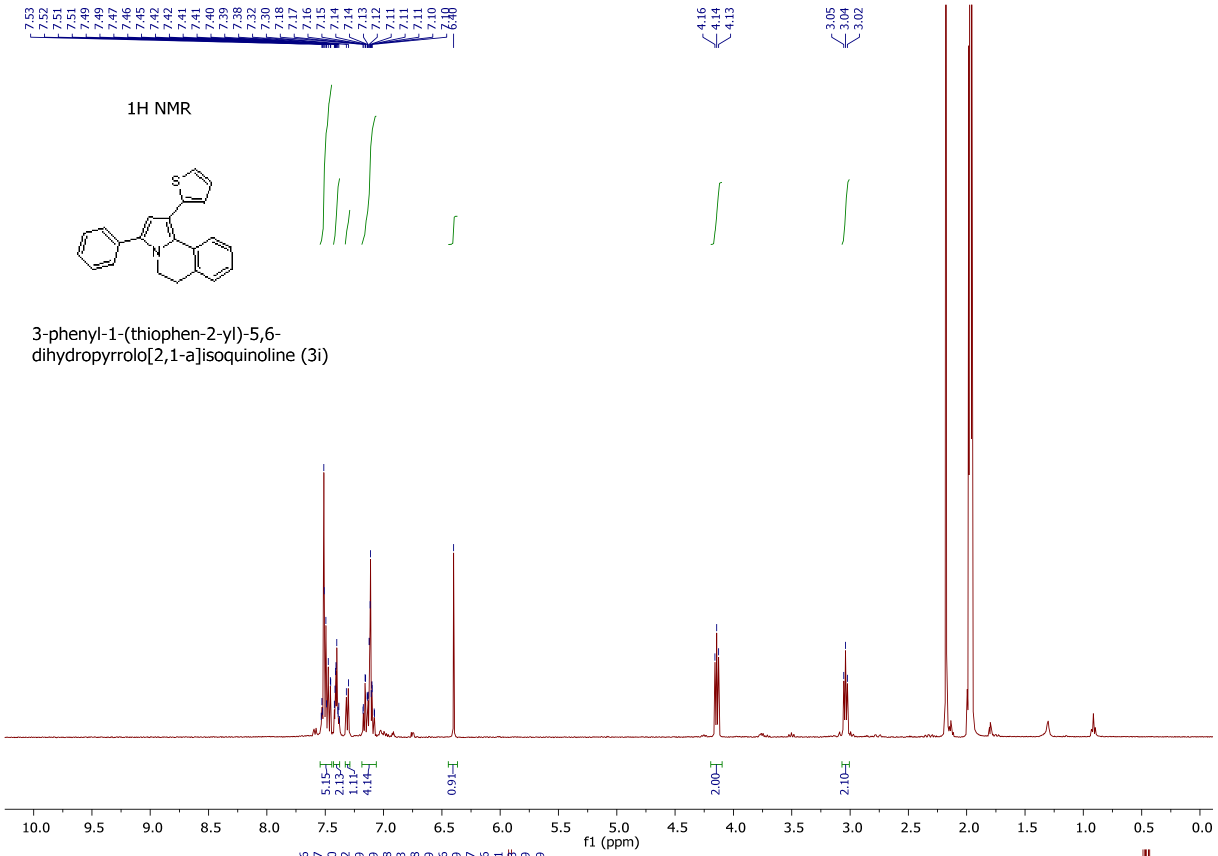

7.53
7.52
7.51
7.51
7.49
7.49
7.47
7.46
7.45
7.42
7.42
7.41
7.41
7.40
7.39
7.38
7.32
7.30
7.18
7.17
7.16
7.15
7.14
7.14
7.13
7.12
7.11
7.11
7.11
7.10
6.40
4.16
4.14
4.13
3.05
3.04
3.02
1H NMR
S
N
3-phenyl-1-(thiophen-2-yl)-5,6-dihydropyrrolo[2,1-a]isoquinoline (3i)
5.15
2.13
1.11
4.14
0.91
2.00
2.10
10.0 9.5 9.0 8.5 8.0 7.5 7.0 6.5 6.0 5.5 5.0 4.5 4.0 3.5 3.0 2.5 2.0 1.5 1.0 0.5 0.0
f1 (ppm)

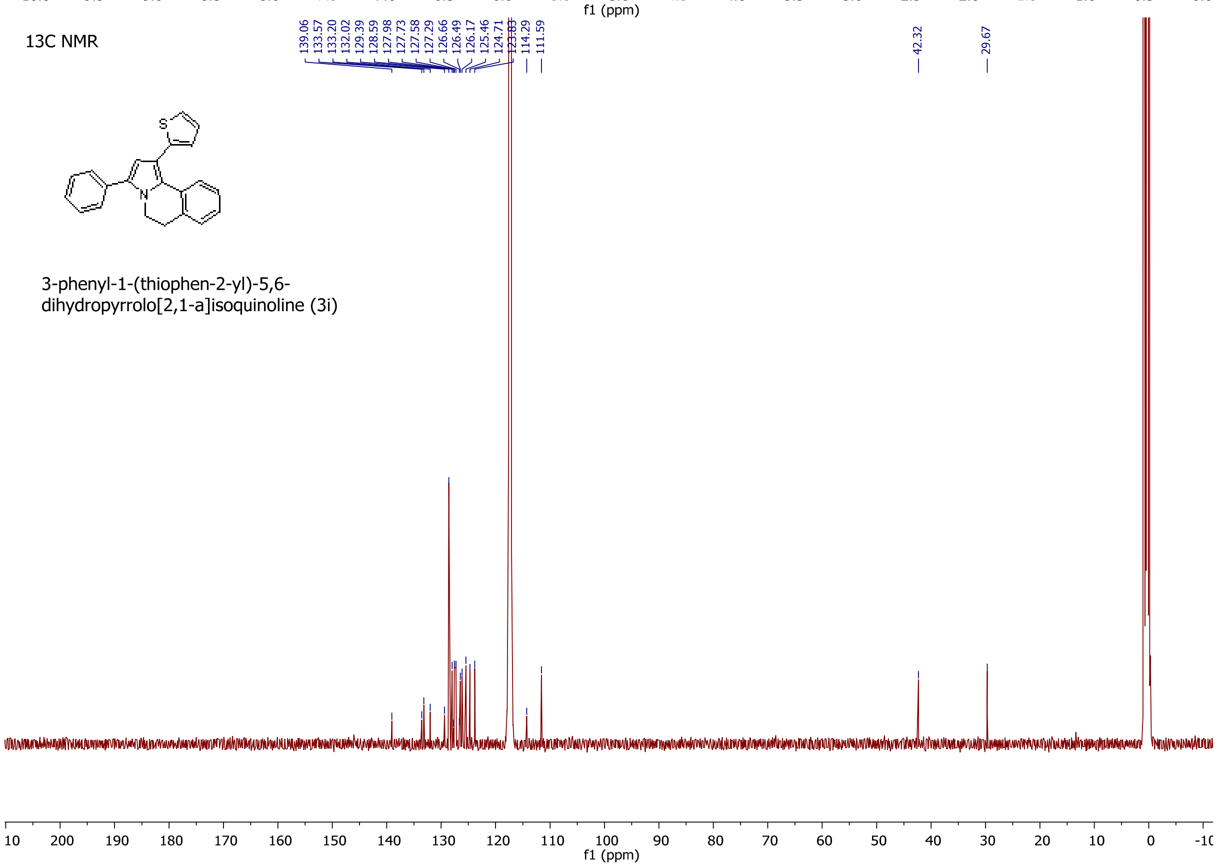

13C NMR
139.06
133.57
133.20
132.02
129.39
128.59
127.98
127.73
127.58
127.29
126.66
126.49
126.17
125.46
124.71
123.83
114.29
111.59
42.32
29.67
S
N
3-phenyl-1-(thiophen-2-yl)-5,6-dihydropyrrolo[2,1-a]isoquinoline (3i)
10 200 190 180 170 160 150 140 130 120 110 100 90 80 70 60 50 40 30 20 10 0 -1(
f1 (ppm)

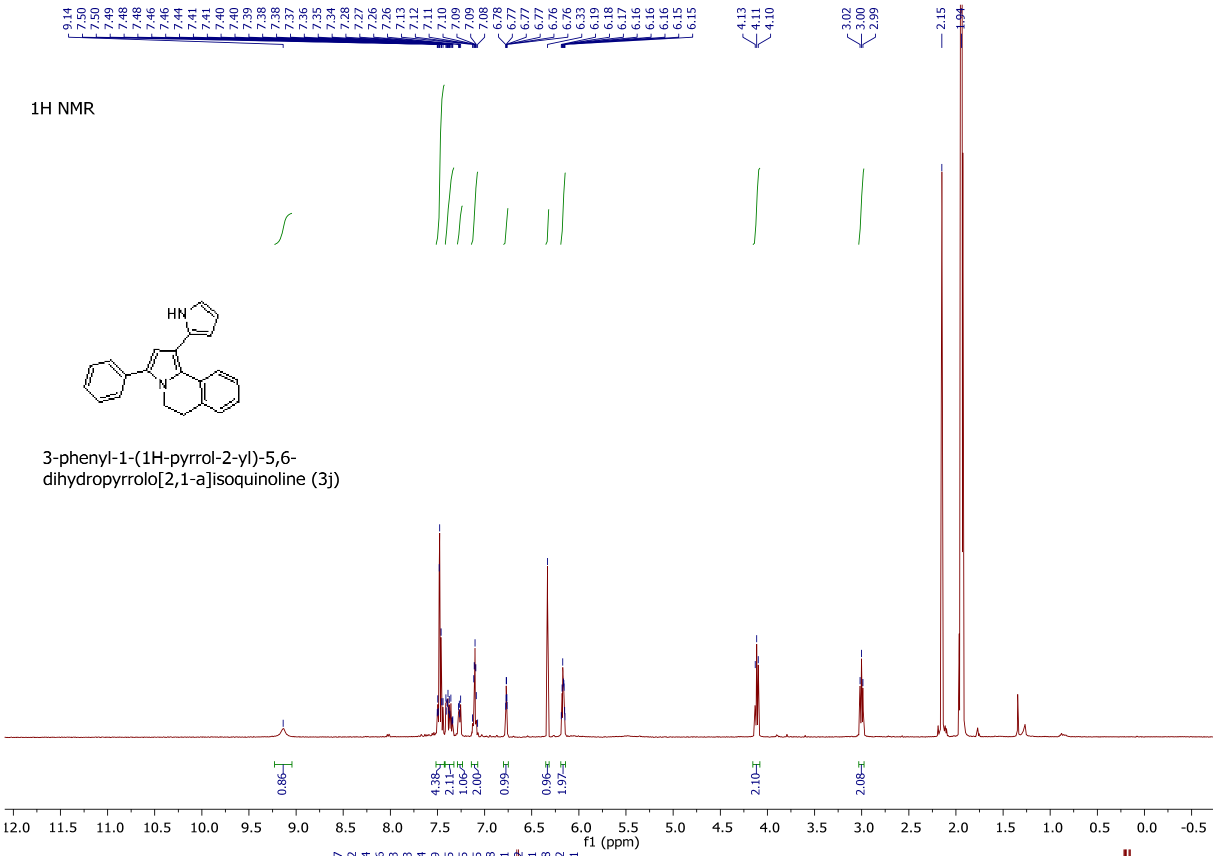
9.14 7.50 7.50 7.49 7.48 7.48 7.46 7.46 7.44 7.41 7.40 7.40 7.39 7.38 7.38 7.37 7.36 7.35 7.34 7.28 7.27 7.26 7.26 7.13 7.12 7.11 7.10 7.09 7.09 7.08 6.78 6.77 6.77 6.77 6.76 6.76 6.33 6.19 6.18 6.17 6.16 6.16 6.16 6.15 6.15 4.13 4.11 4.10 3.02 3.00 2.99 2.15 1.94
1H NMR
HN
N
3-phenyl-1-(1H-pyrrol-2-yl)-5,6-
dihydropyrrolo[2,1-a]isoquinoline (3j)
0.86 4.38 2.11 1.06 2.00 0.99 0.96 1.97 2.10 2.08
12.0 11.5 11.0 10.5 10.0 9.5 9.0 8.5 8.0 7.5 7.0 6.5 6.0 5.5 5.0 4.5 4.0 3.5 3.0 2.5 2.0 1.5 1.0 0.5 0.0 -0.5
f1 (ppm)

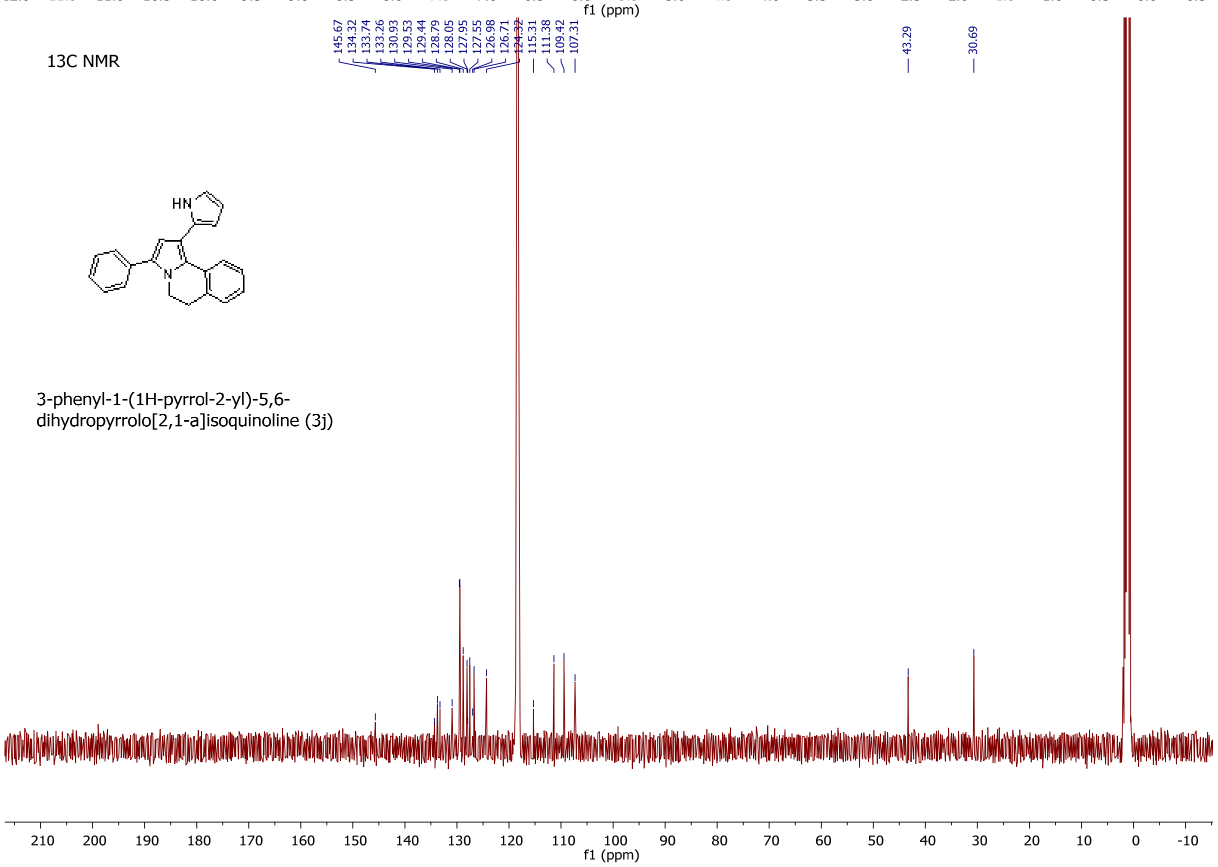
145.67 134.32 133.74 133.26 130.93 129.53 129.44 128.79 128.05 127.95 127.55 126.98 126.71 124.32 115.31 111.38 109.42 107.31 43.29 30.69
13C NMR
HN
N
3-phenyl-1-(1H-pyrrol-2-yl)-5,6-
dihydropyrrolo[2,1-a]isoquinoline (3j)
210 200 190 180 170 160 150 140 130 120 110 100 90 80 70 60 50 40 30 20 10 0 -10
f1 (ppm)

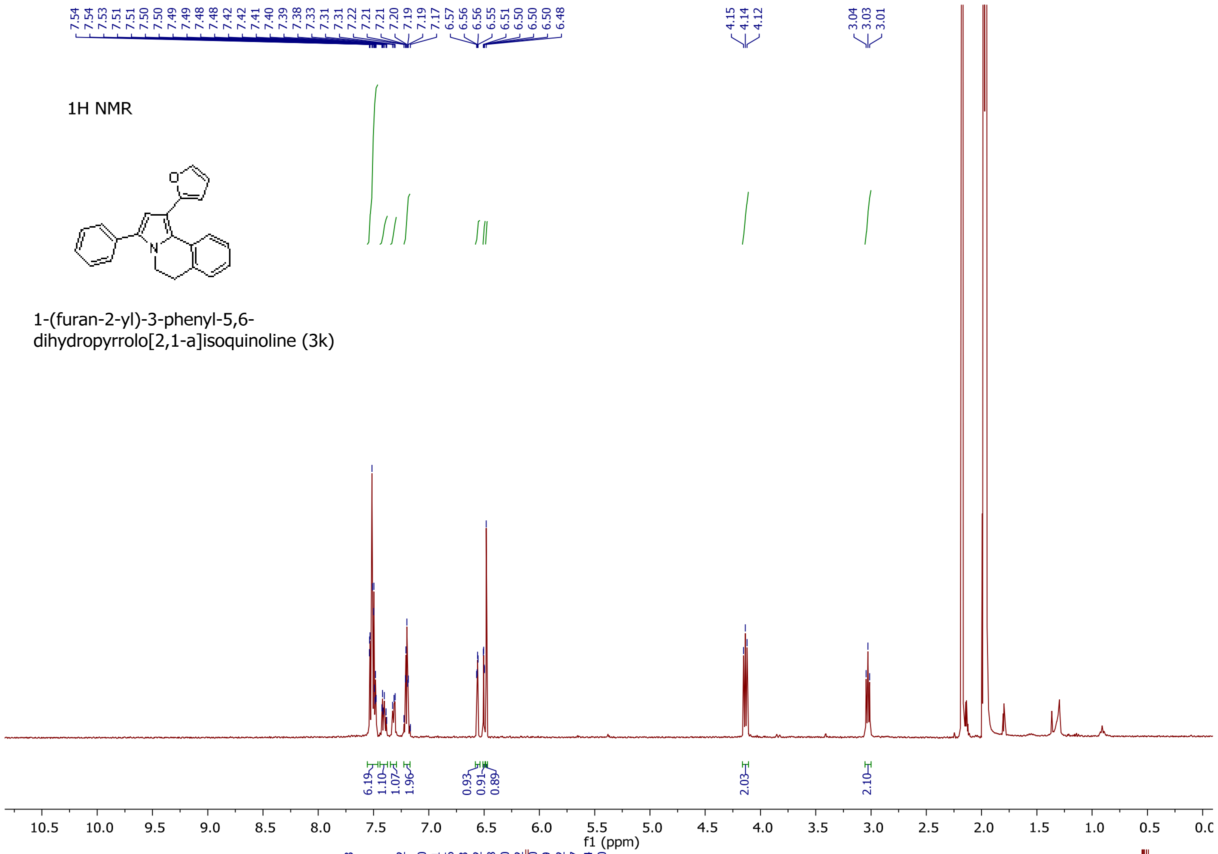
1H NMR
1-(furan-2-yl)-3-phenyl-5,6-dihydropyrrolo[2,1-a]isoquinoline (3k)
7.54 7.54 7.53 7.51 7.51 7.50 7.50 7.49 7.49 7.48 7.48 7.42 7.42 7.41 7.40 7.39 7.38 7.33 7.31 7.31 7.22 7.21 7.21 7.20 7.19 7.19 7.17 6.57 6.56 6.56 6.55 6.51 6.50 6.50 6.50 6.48
4.15 4.14 4.12
3.04 3.03 3.01
6.19 1.10 1.07 1.96 0.93 0.91 0.89 2.03 2.10
10.5 10.0 9.5 9.0 8.5 8.0 7.5 7.0 6.5 6.0 5.5 5.0 4.5 4.0 3.5 3.0 2.5 2.0 1.5 1.0 0.5 0.0
f1 (ppm)

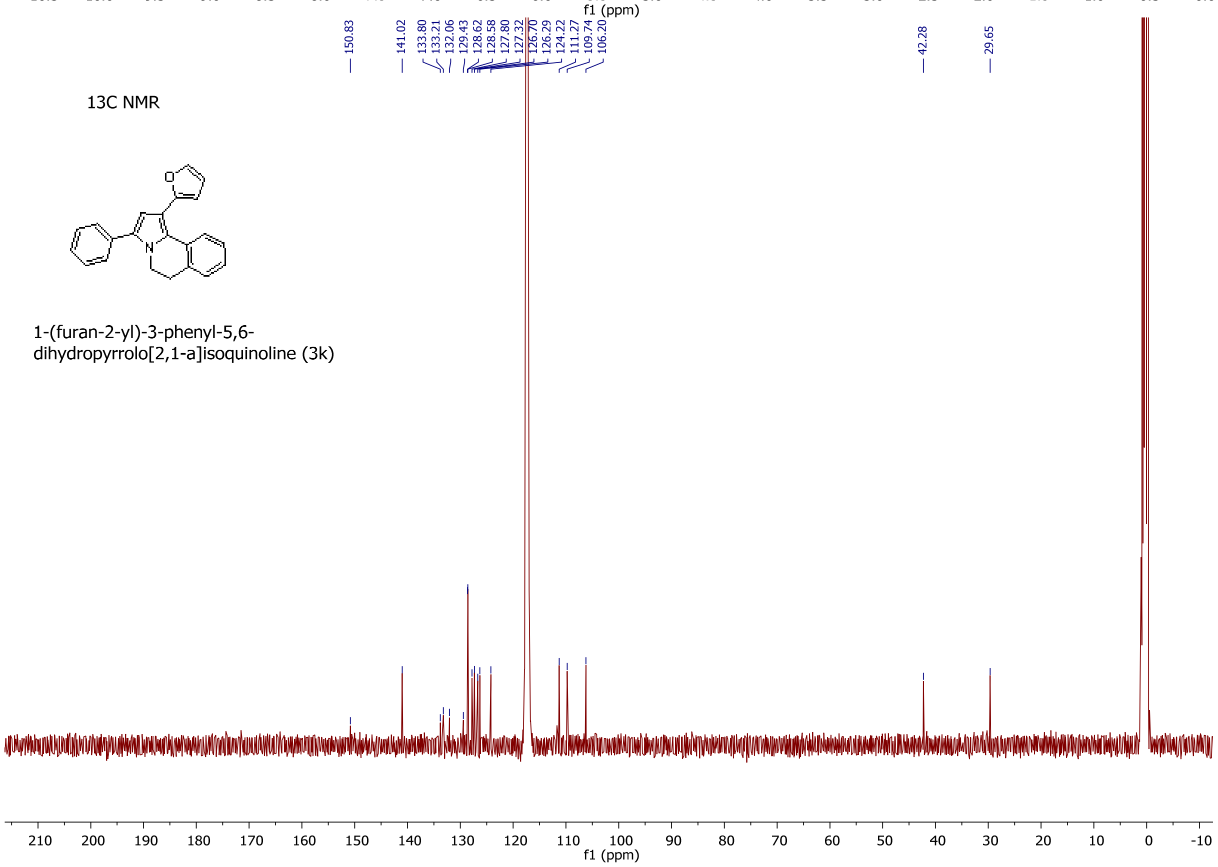
13C NMR
1-(furan-2-yl)-3-phenyl-5,6-dihydropyrrolo[2,1-a]isoquinoline (3k)
150.83
141.02
133.80 133.21 132.06 129.43 128.62 128.58 127.80 127.32 126.70 126.29 124.22 111.27 109.74 106.20
42.28
29.65
210 200 190 180 170 160 150 140 130 120 110 100 90 80 70 60 50 40 30 20 10 0 -10
f1 (ppm)

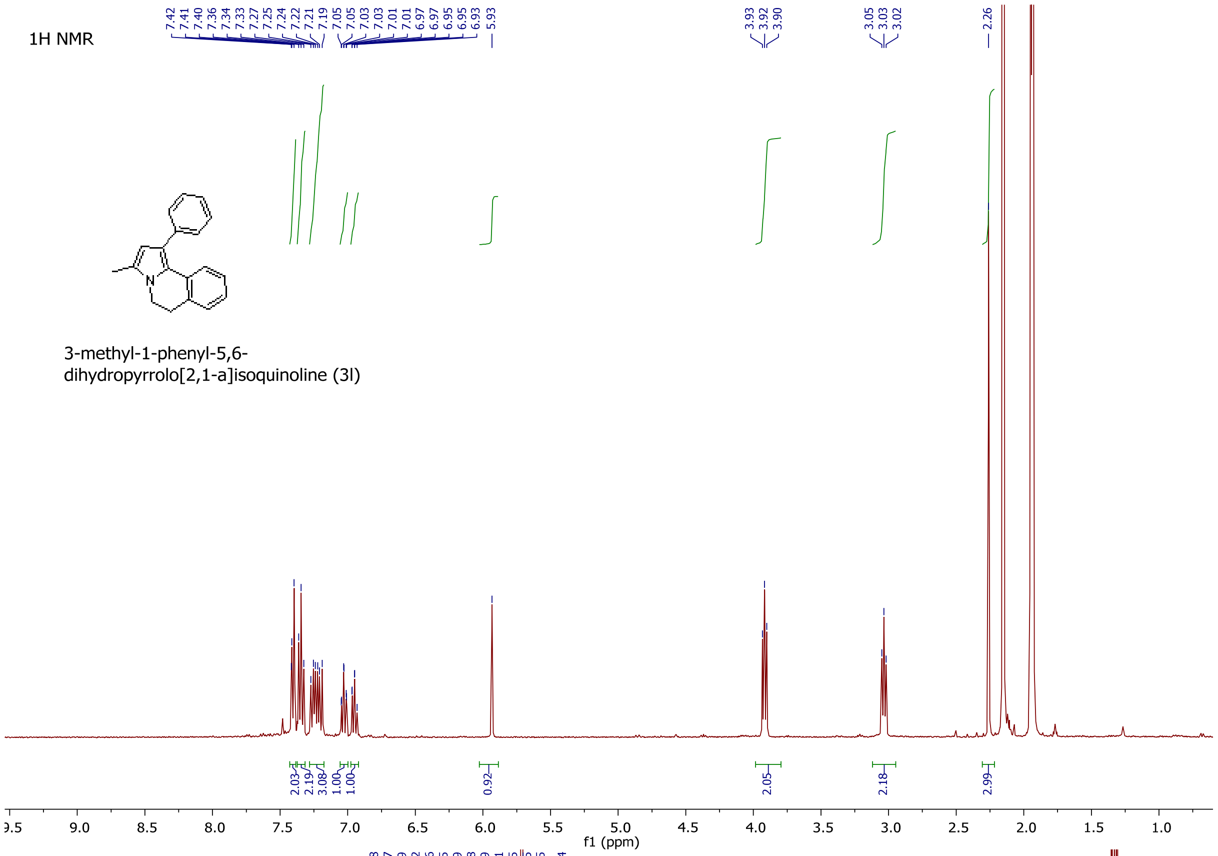
1H NMR
7.42
7.41
7.40
7.36
7.34
7.33
7.27
7.25
7.24
7.22
7.21
7.19
7.05
7.05
7.03
7.03
7.01
7.01
6.97
6.97
6.95
6.95
6.93
5.93
3.93
3.92
3.90
3.05
3.03
3.02
2.26
N
3-methyl-1-phenyl-5,6-dihydropyrrolo[2,1-a]isoquinoline (3l)
2.03
2.19
3.08
1.00
1.00
0.92
2.05
2.18
2.99
f1 (ppm)

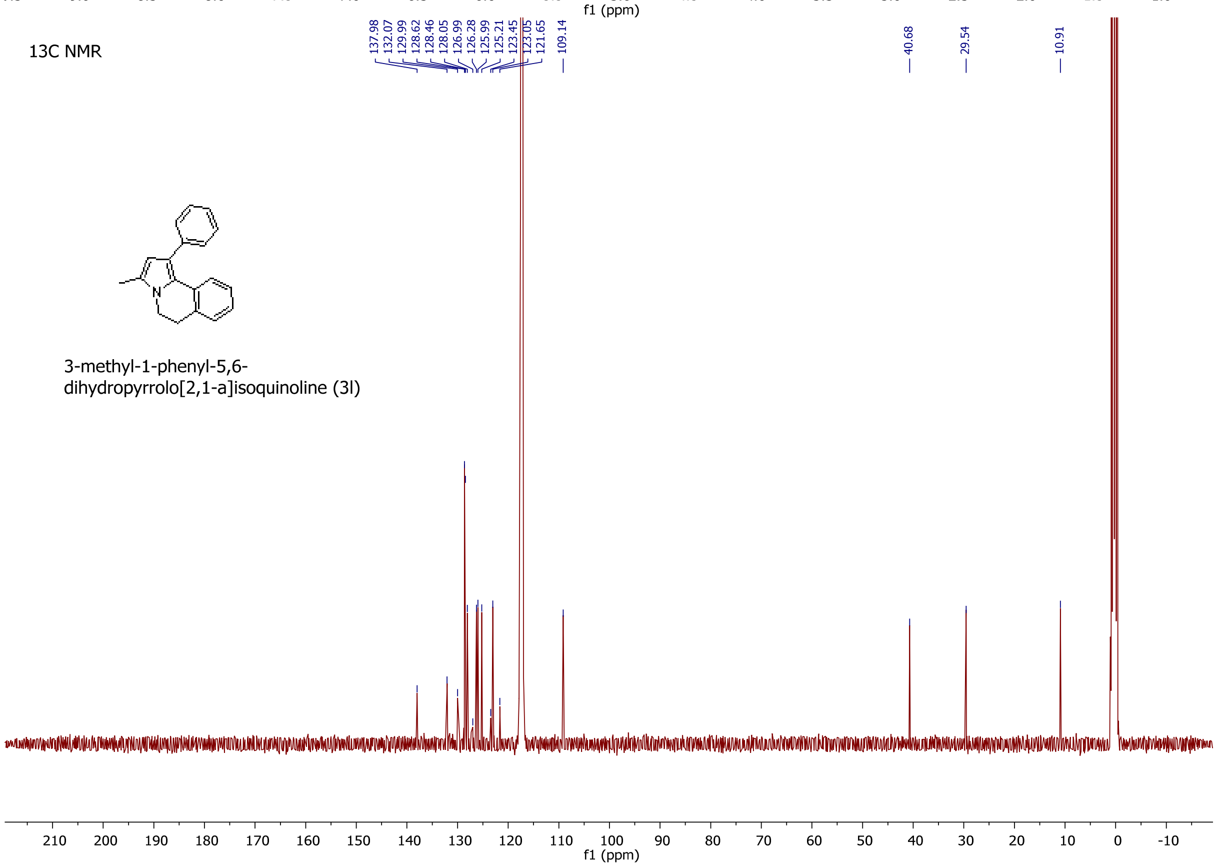
13C NMR
137.98
132.07
129.99
128.62
128.46
128.05
126.99
126.28
125.99
125.21
123.45
123.05
121.65
109.14
40.68
29.54
10.91
N
3-methyl-1-phenyl-5,6-dihydropyrrolo[2,1-a]isoquinoline (3l)
f1 (ppm)

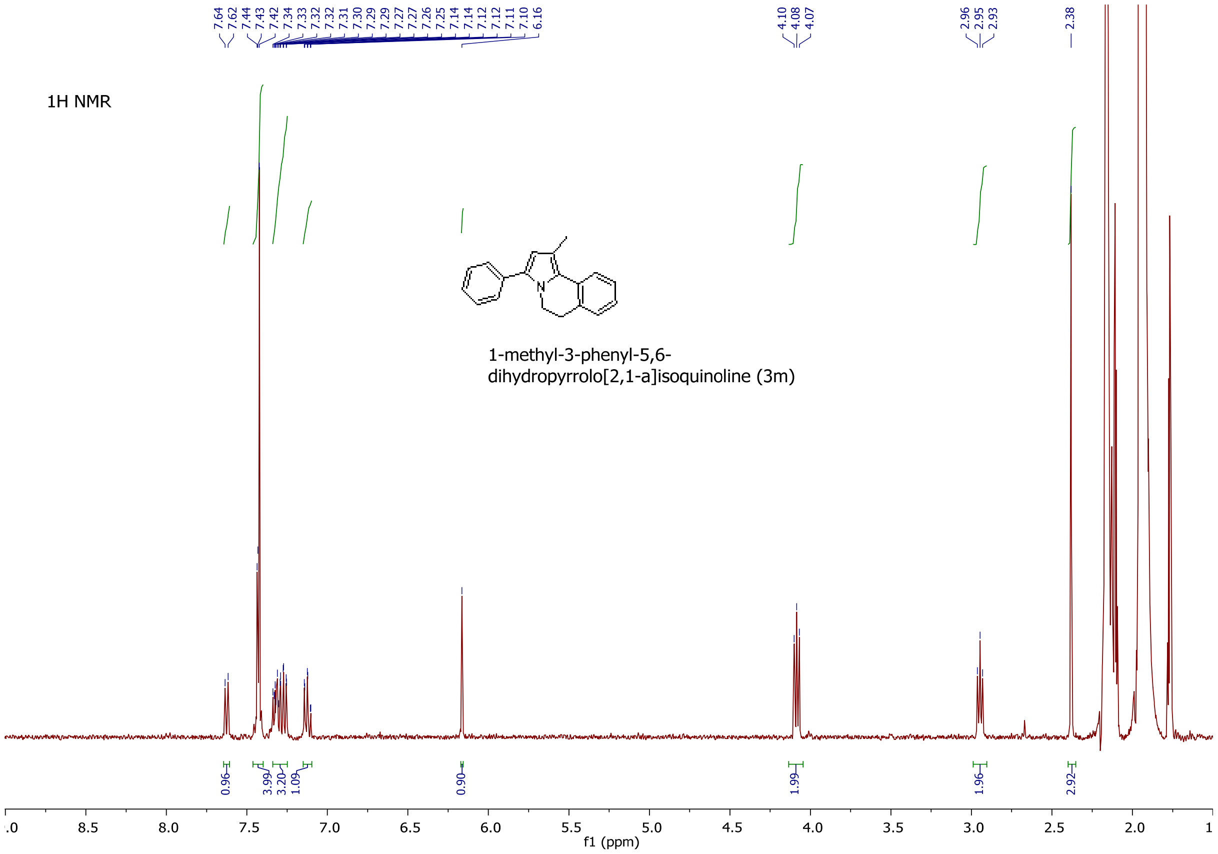
1H NMR
1-methyl-3-phenyl-5,6-dihydropyrrolo[2,1-a]isoquinoline (3m)
7.64
7.62
7.44
7.43
7.42
7.34
7.33
7.32
7.32
7.31
7.30
7.29
7.29
7.27
7.27
7.26
7.25
7.14
7.14
7.12
7.12
7.11
7.10
6.16
4.10
4.08
4.07
2.96
2.95
2.93
2.38
0.96
3.99
3.20
1.09
0.90
1.99
1.96
2.92
f1 (ppm)

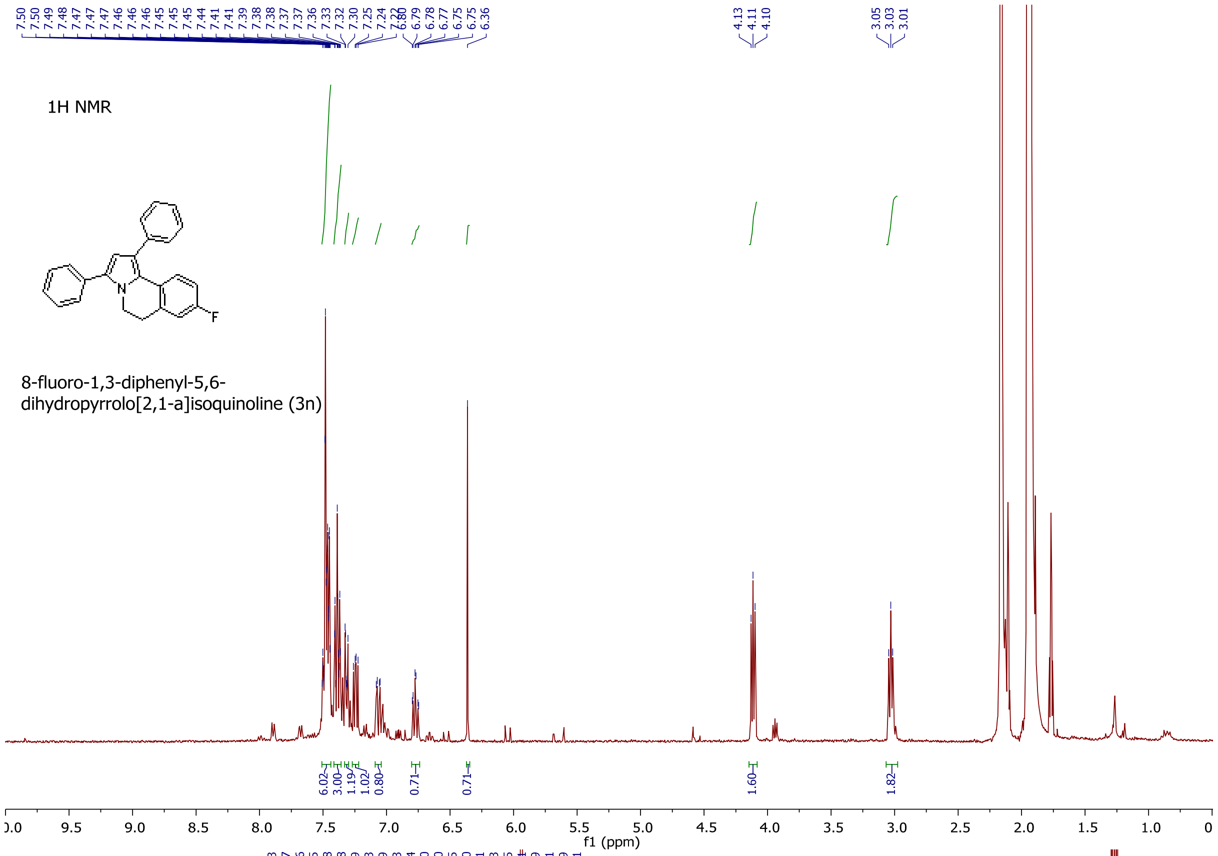

1H NMR
8-fluoro-1,3-diphenyl-5,6-dihydropyrrolo[2,1-a]isoquinoline (3n)
f1 (ppm)

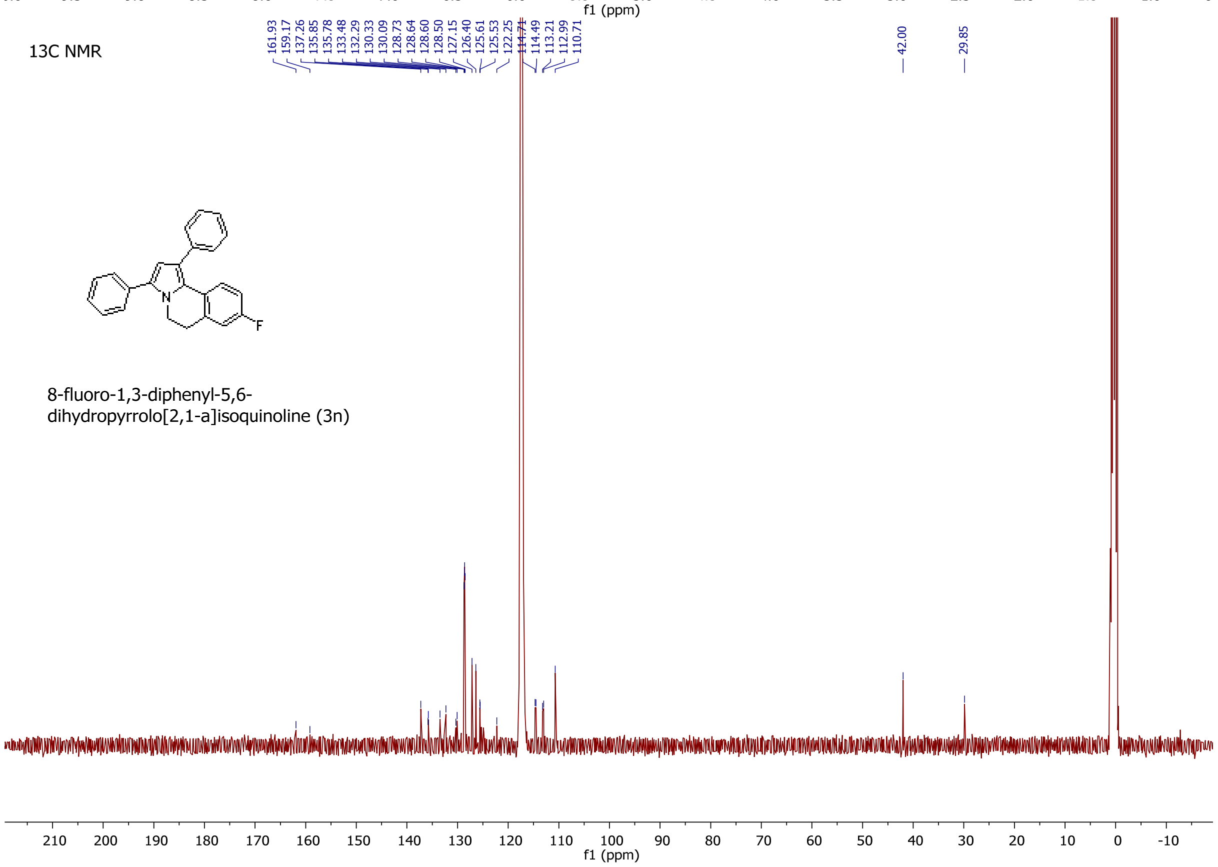

13C NMR
8-fluoro-1,3-diphenyl-5,6-dihydropyrrolo[2,1-a]isoquinoline (3n)
f1 (ppm)

19F NMR

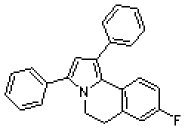

8-fluoro-1,3-diphenyl-5,6-
dihydropyrrolo[2,1-a]isoquinoline (3n)

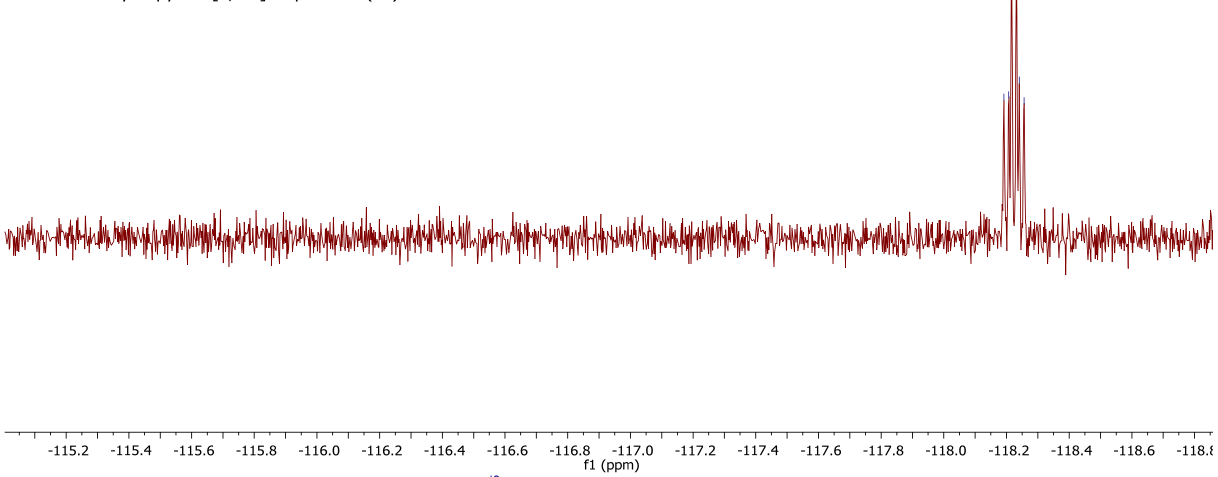

19F NMR

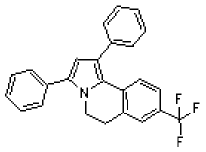

1,3-diphenyl-8-(trifluoromethyl)-5,6-
dihydropyrrolo[2,1-a]isoquinoline (3o)

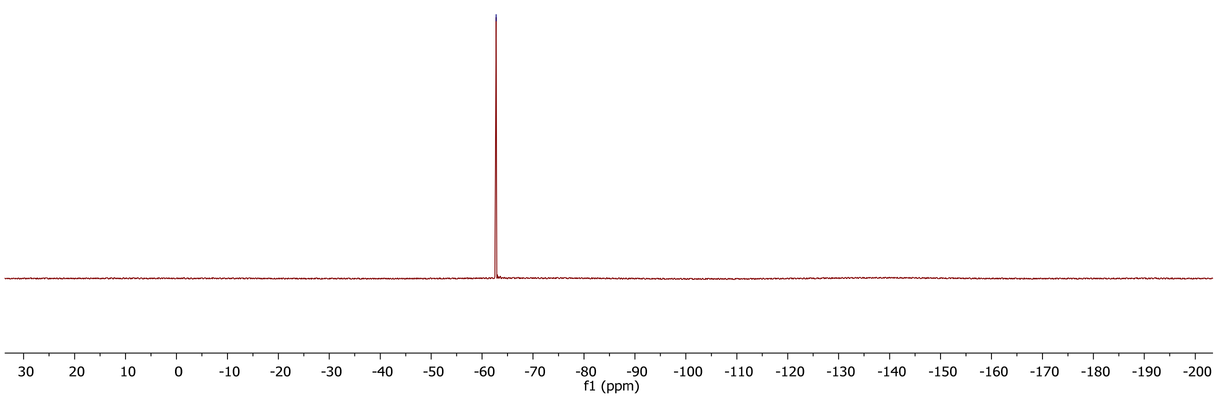

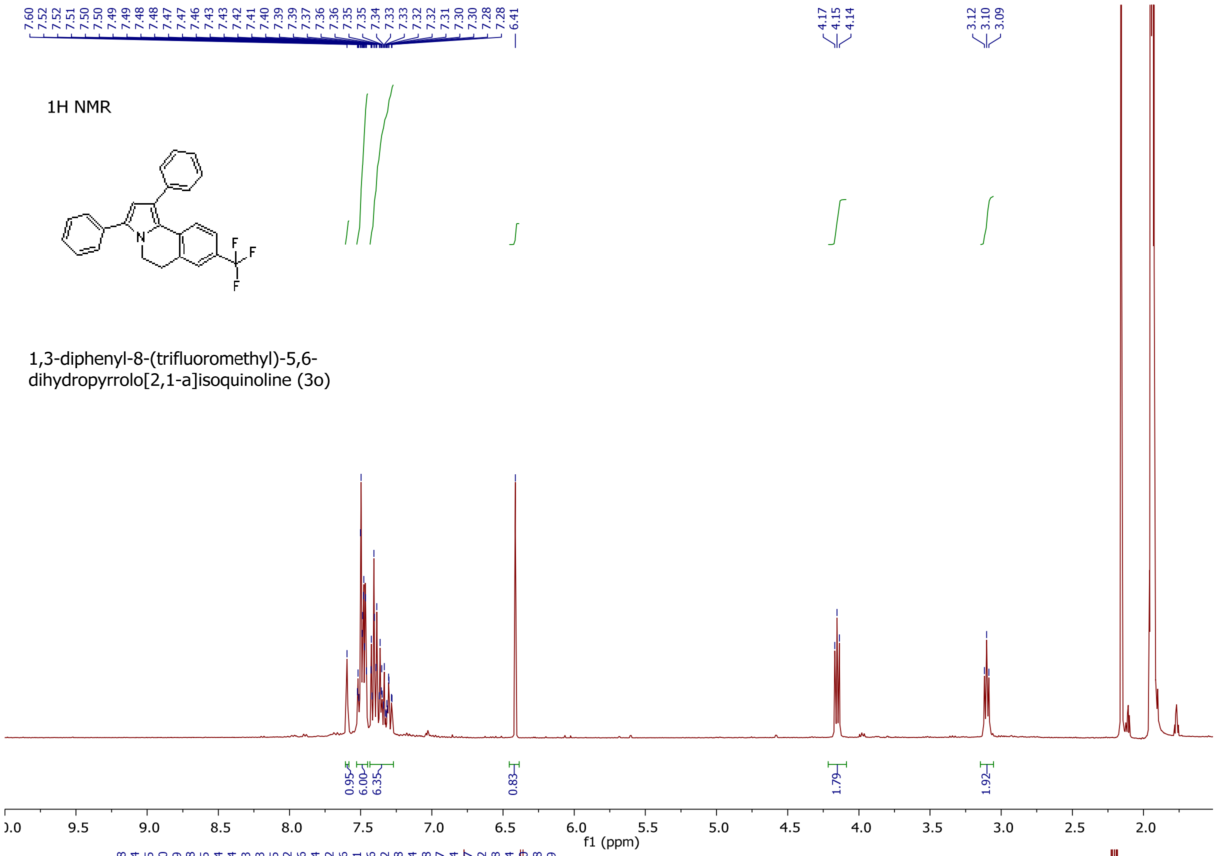
7.60
7.52
7.51
7.50
7.50
7.49
7.49
7.48
7.48
7.47
7.47
7.46
7.43
7.43
7.42
7.41
7.40
7.39
7.39
7.37
7.36
7.35
7.35
7.34
7.33
7.33
7.32
7.32
7.31
7.30
7.30
7.28
7.28
6.41
4.17
4.15
4.14
3.12
3.10
3.09
1H NMR
F
F
F
N
1,3-diphenyl-8-(trifluoromethyl)-5,6-
dihydropyrrolo[2,1-a]isoquinoline (3o)
0.95
6.00
6.35
0.83
1.79
1.92
f1 (ppm)

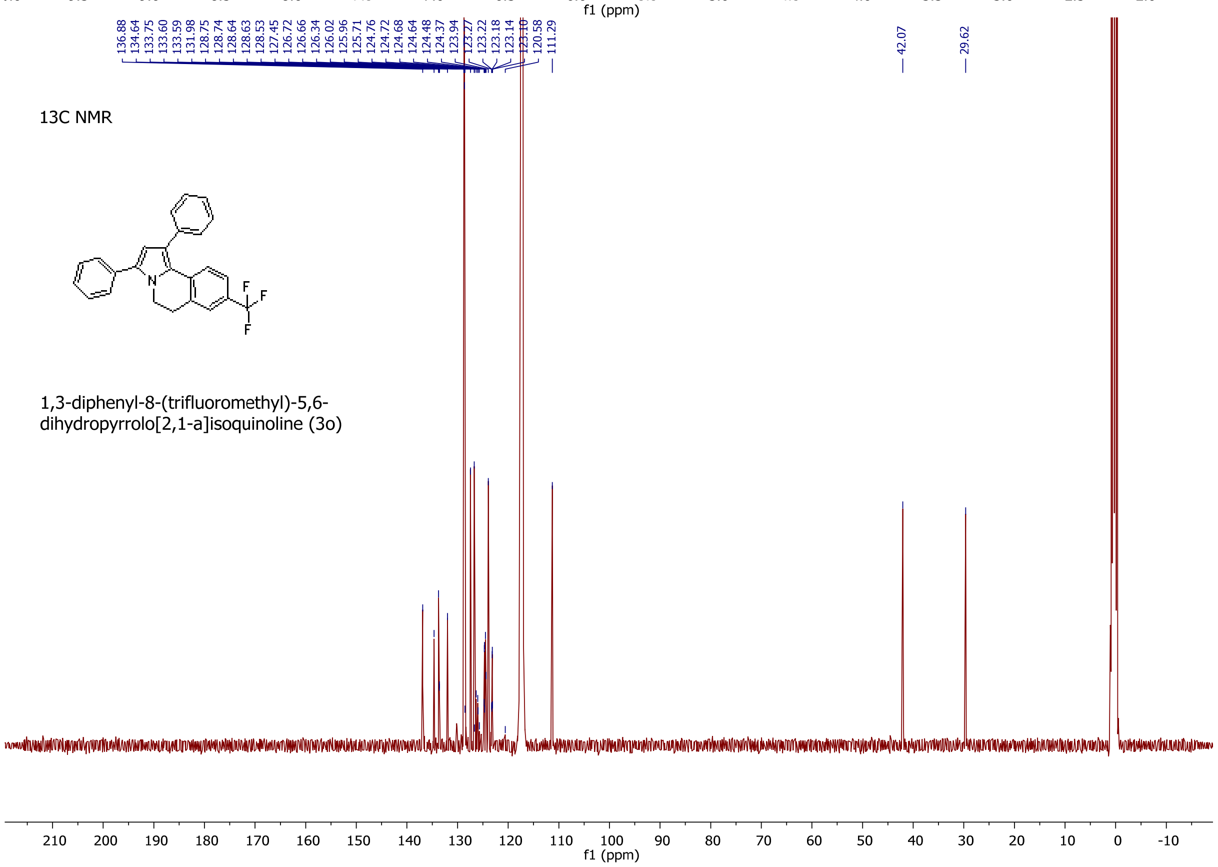
136.88
134.64
133.75
133.60
133.59
131.98
128.75
128.74
128.64
128.63
128.53
127.45
126.72
126.66
126.34
126.02
125.96
125.71
124.76
124.72
124.68
124.64
124.48
124.37
123.94
123.77
123.22
123.18
123.14
120.58
111.29
42.07
29.62
13C NMR
F
F
F
N
1,3-diphenyl-8-(trifluoromethyl)-5,6-
dihydropyrrolo[2,1-a]isoquinoline (3o)
f1 (ppm)

1H NMR

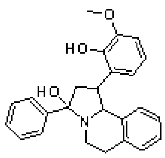

1-(2-hydroxy-3-methoxyphenyl)-
3-phenyl-1,2,3,5,6,10b-hexahydro-
pyrrolo[2,1-a]isoquinolin-3-ol (7p)

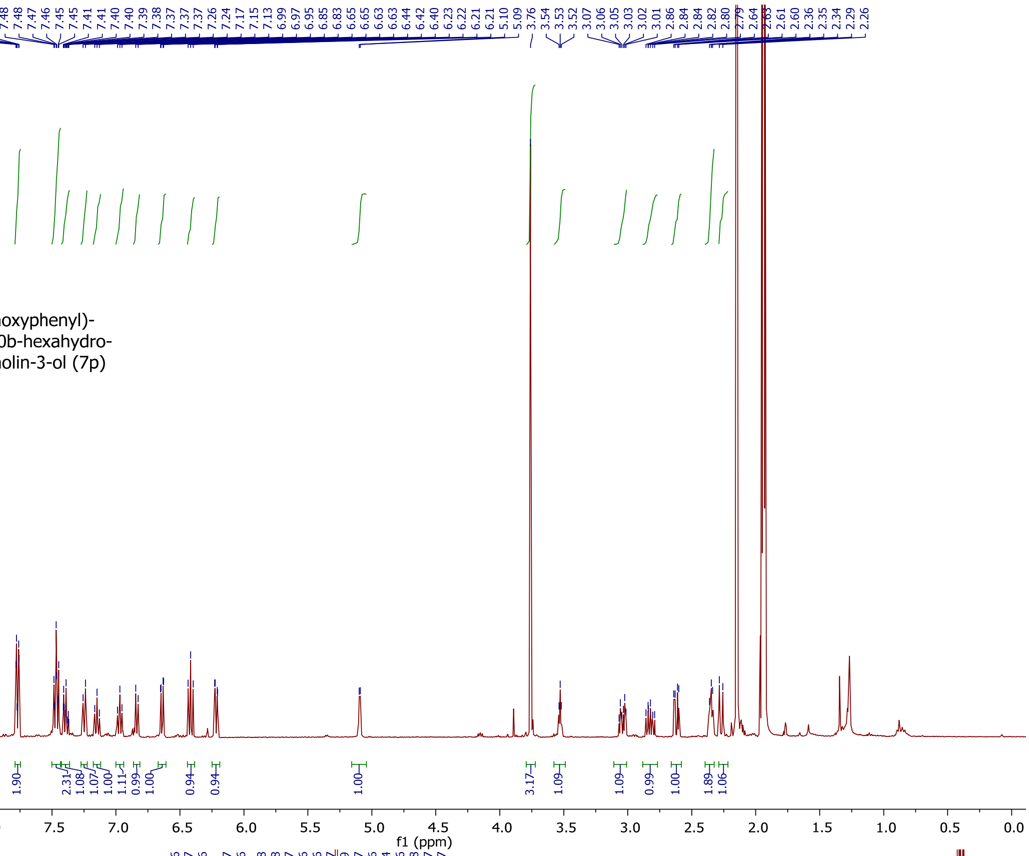

13C NMR

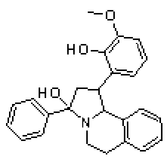

1-(2-hydroxy-3-methoxyphenyl)-
3-phenyl-1,2,3,5,6,10b-hexahydro-
pyrrolo[2,1-a]isoquinolin-3-ol (7p)

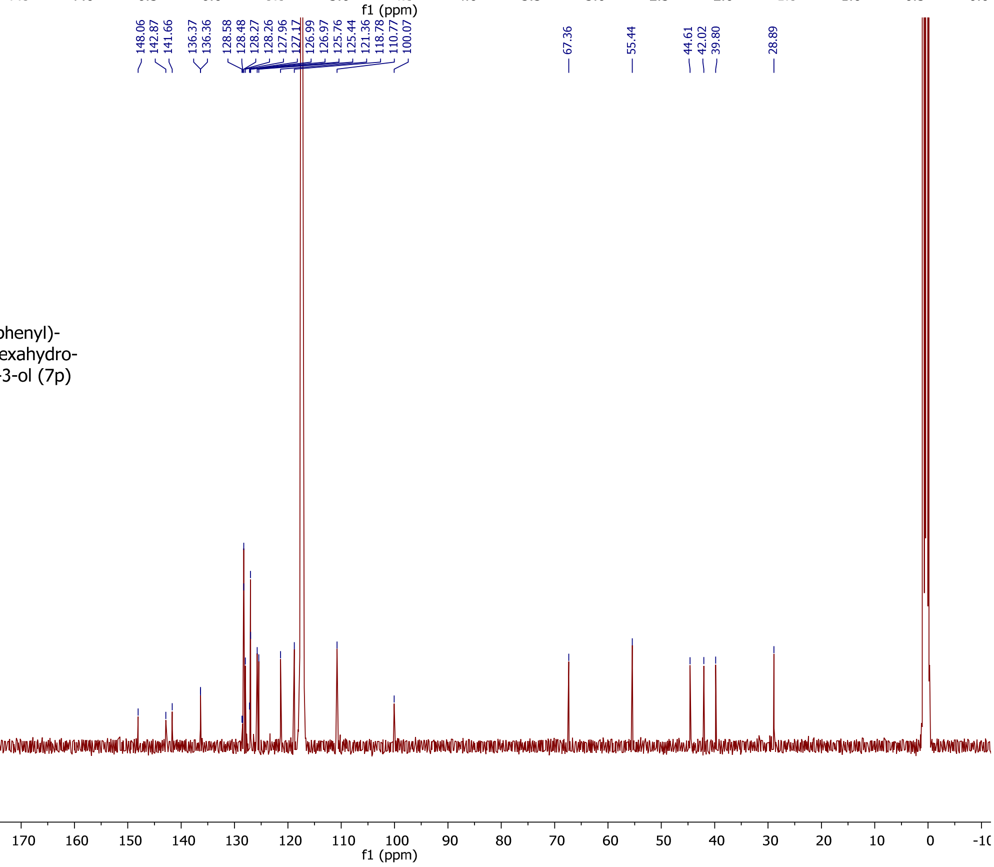

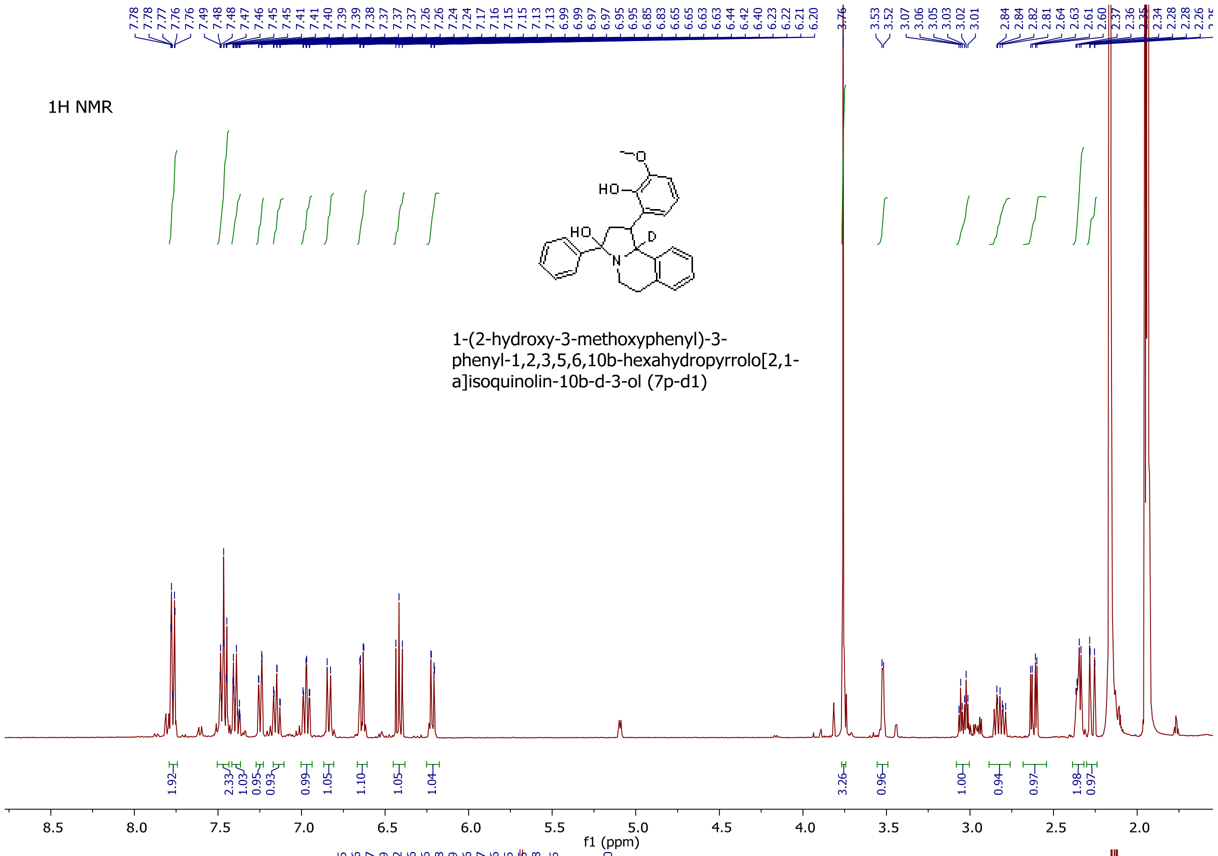

1-(2-hydroxy-3-methoxyphenyl)-3-phenyl-1,2,3,5,6,10b-hexahydropyrrolo[2,1-a]isoquinolin-10b-d-3-ol (7p-d1)

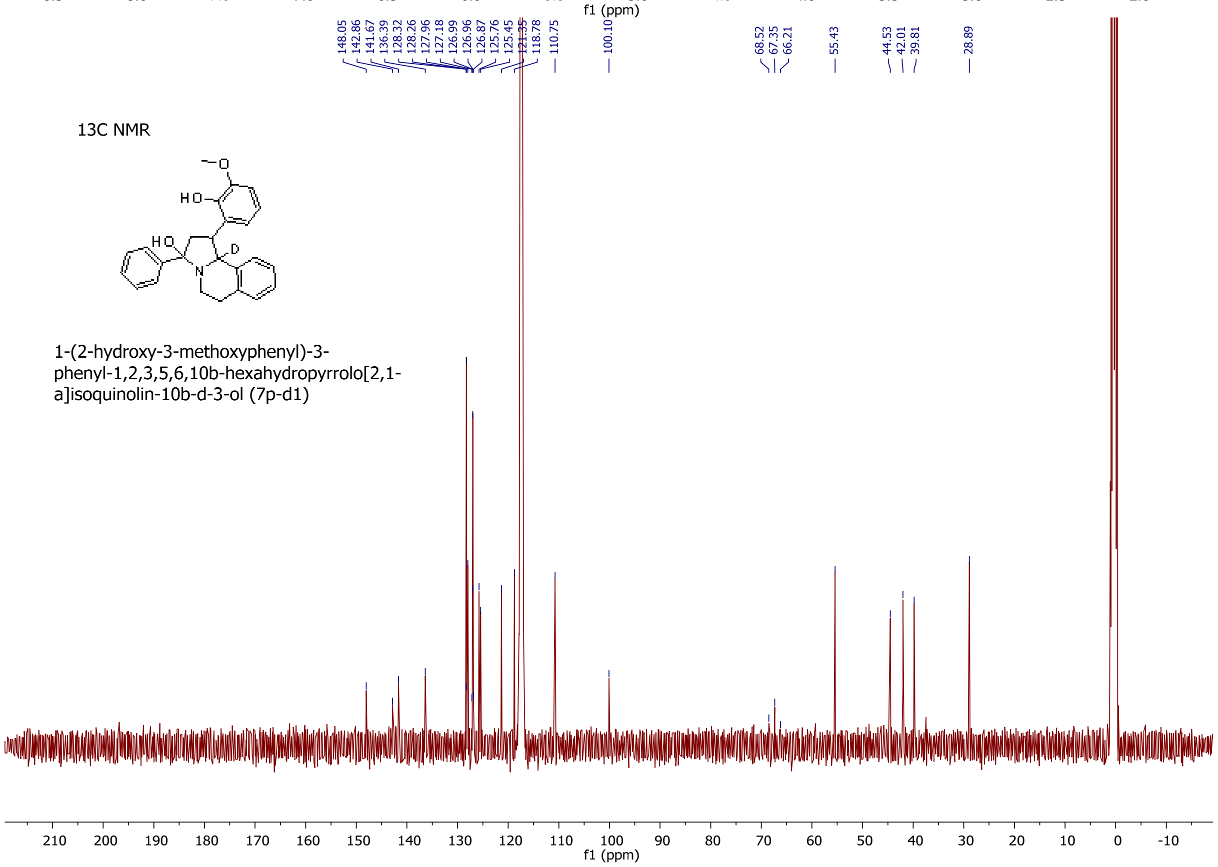

1-(2-hydroxy-3-methoxyphenyl)-3-phenyl-1,2,3,5,6,10b-hexahydropyrrolo[2,1-a]isoquinolin-10b-d-3-ol (7p-d1)

Preparation of 2-bromo-1-(4-fluorophenyl)-3-phenyl-5,6-dihydropyrrolo[2,1-a]isoquinoline 8d

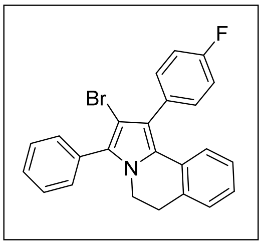

N-bromosuccine imide (1.8 mg, 10 μmol) was added to a solution of DHPIQ **3d** (3.5 mg, 10 μmol) in CD_3CN (0.5 mL). The reaction mixture was stirred at room temperature (ca. 25°C) for 10 min. Reaction progress was monitored by ^1H NMR. After reaction completion, the white solid was separated, washed with a small amount of CD_3CN (ca. 0.1 mL) and dried in vacuum at room temperature. White solid (yield 92%). ^1H NMR (400 MHz, CD_3CN) δ 7.49 (dq, *J* = 13.2, 7.2 Hz, 5H), 7.39 (dd, *J* = 8.7, 5.6 Hz, 2H), 7.22 (q, *J* = 8.9, 8.5 Hz, 3H), 7.13 – 7.05 (m, 1H), 6.96 (t, *J* = 7.6 Hz, 1H), 6.90 (d, *J* = 7.8 Hz, 1H), 3.97 (t, *J* = 6.4 Hz, 2H), 3.02 (t, *J* = 6.4 Hz, 2H). ^{13}C NMR (101 MHz, CD_3CN) δ 163.1 (d, *J* = 244.1 Hz), 133.9, 133.5 (d, *J* = 8.1 Hz), 131.4, 129.5, 129.4, 129.2, 129.1, 127.4, 127.3, 124.8, 116.4 (d, *J* = 21.5 Hz), 99.2, 43.8, 30.2. ^{19}F NMR (376 MHz, CD_3CN) δ -116.79 (ddd, *J* = 14.6, 9.1, 5.6 Hz).

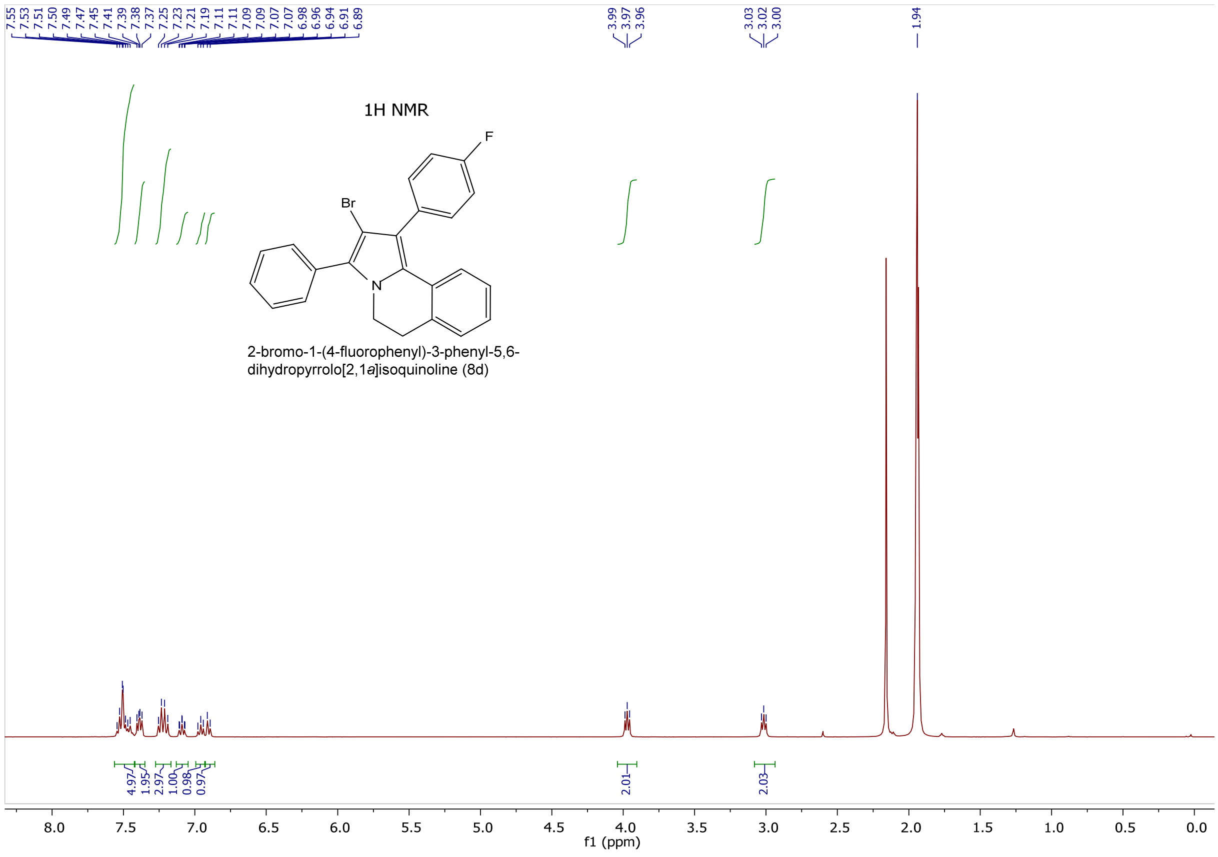

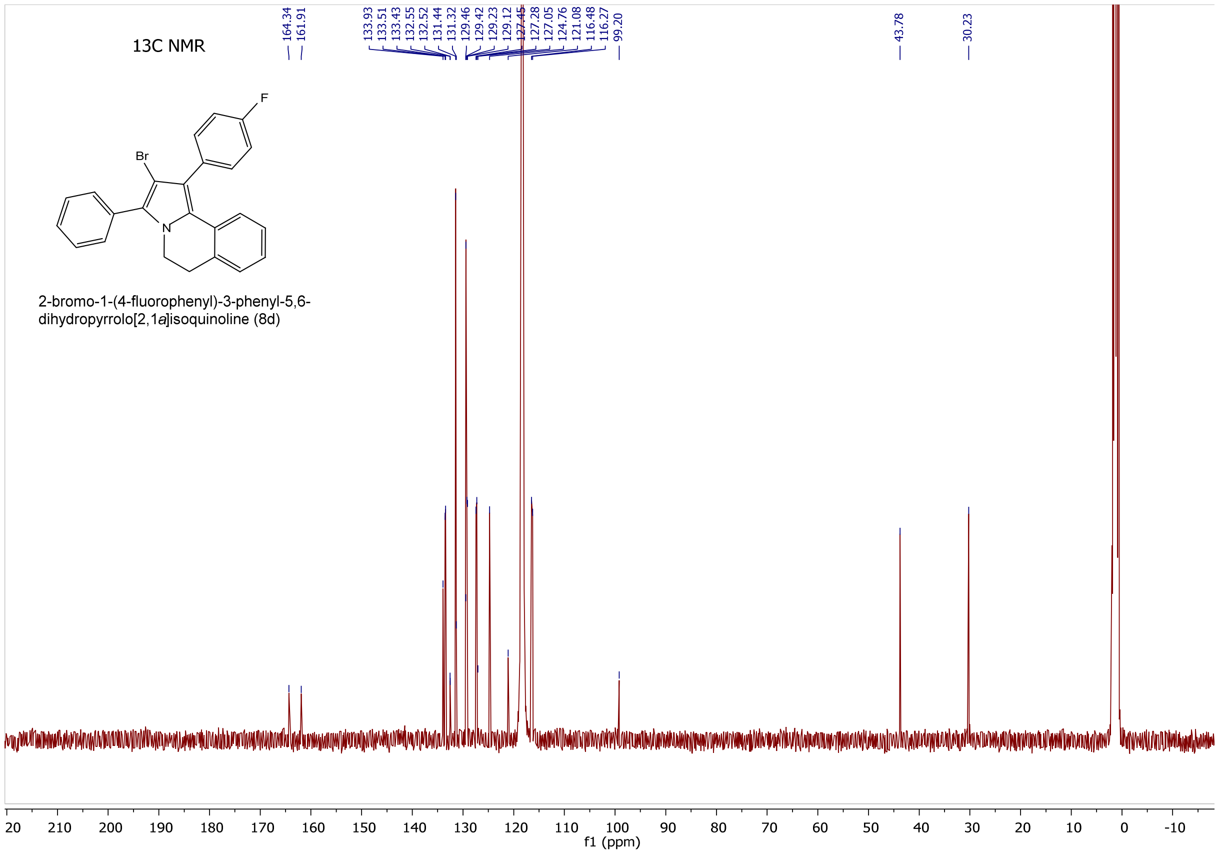
13C NMR
2-bromo-1-(4-fluorophenyl)-3-phenyl-5,6-dihydropyrrolo[2,1a]isoquinoline (8d)
164.34
161.91
133.93
133.51
133.43
132.55
132.52
131.44
131.32
129.46
129.42
129.23
129.12
127.45
127.28
127.05
124.76
121.08
116.48
116.27
99.20
43.78
30.23
Br
F
N
f1 (ppm)

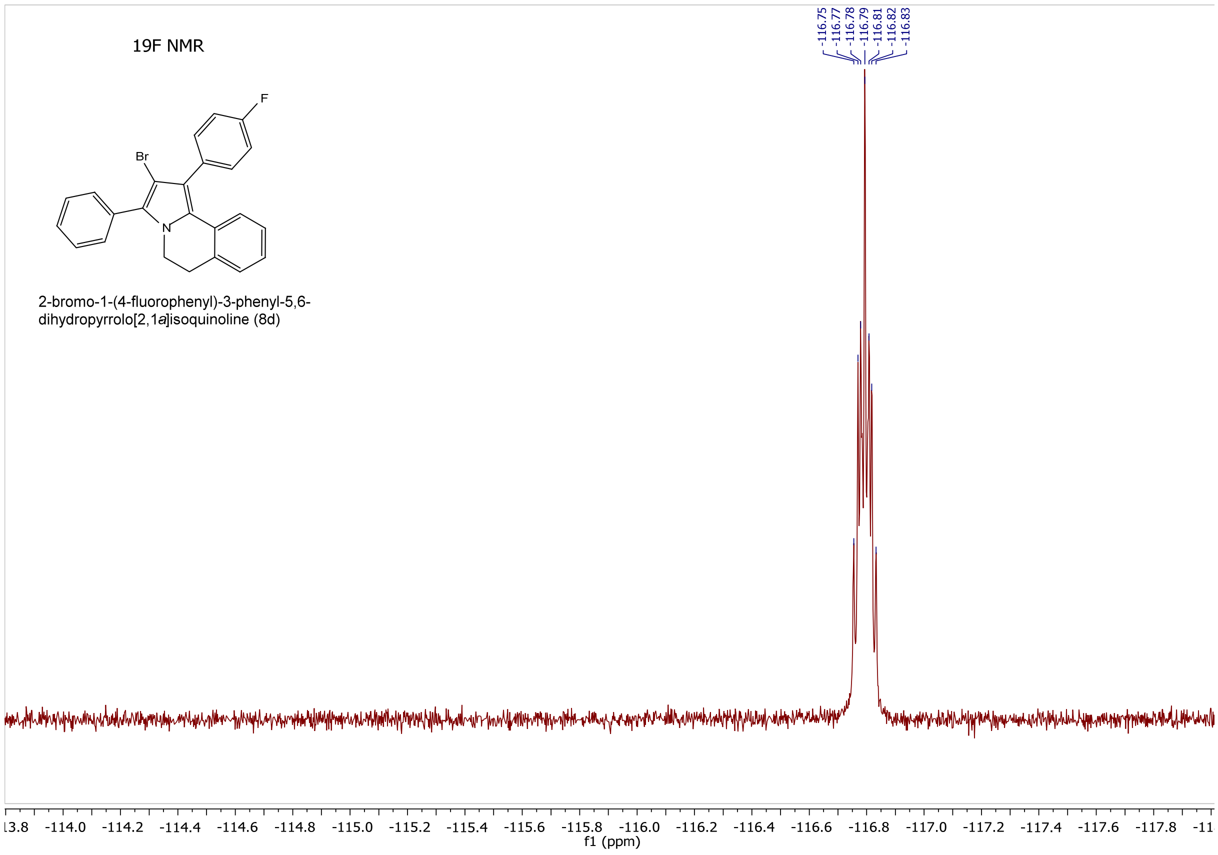
19F NMR
2-bromo-1-(4-fluorophenyl)-3-phenyl-5,6-dihydropyrrolo[2,1a]isoquinoline (8d)
-116.75
-116.77
-116.78
-116.79
-116.81
-116.82
-116.83
Br
F
N
f1 (ppm)

3-(perfluorophenyl)-1-phenylpropan-1-one (4q)

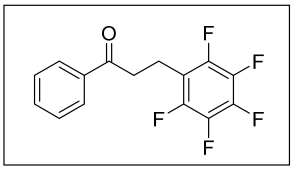

^{1}H NMR (400 MHz, Acetonitrile-d_3) δ 8.00 – 7.91 (m, 1H), 7.65 – 7.58 (m, 1H), 7.53 – 7.47 (m, 1H), 3.36 (t, J = 7.4 Hz, 1H), 3.08 (t, J = 7.4 Hz, 1H).

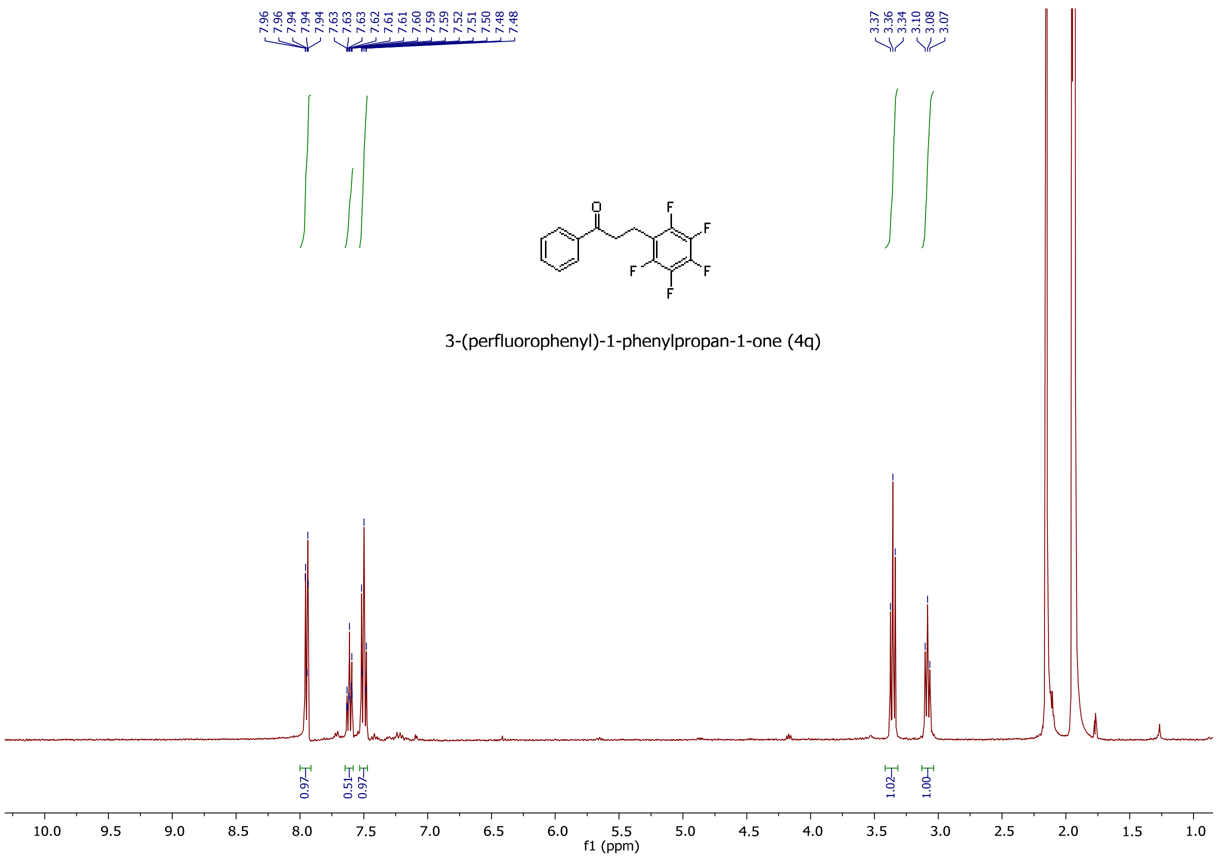

Supplementary References

1. JulioMontes-Avila, Díaz-Camacho SP, Sicairos-Félix J, Delgado-Vargas F, Rivero IA. Solution-phase parallel synthesis of substituted chalcones and their antiparasitary activity against Giardia lamblia. *Biorg Med Chem* **17**, 6780-6785 (2009).

2. Hayat F, Salahuddin A, Umar S, Azam A. Synthesis, characterization, antiamoebic activity and cytotoxicity of novel series of pyrazoline derivatives bearing quinoline tail. *Eur J Med Chem* **45**, 4669-4675 (2010).

3. Fang F, Li Y, Tian S-K. Stereoselective Olefination of N-Sulfonyl Imines with Stabilized Phosphonium Ylides for the Synthesis of Electron-Deficient Alkenes. *Eur J Org Chem*, 1084–1091 (2011).

4. Braun RU, Ansorge M, Müller TJJ. Coupling–Isomerization Synthesis of Chalcones. *Chem Eur J* **12**, 9081–9094 (2006).

5. Kumar V*, et al.* Novel Chalcone Derivatives as Potent Nrf2 Activators in Mice and Human Lung Epithelial Cells. *J Med Chem* **54**, 4147–4159 (2011).

6. Ranu BC, Jana R. Catalysis by Ionic Liquid. A Green Protocol for the Stereoselective Debromination of vicinal-Dibromides by [pmIm]BF4 under Microwave Irradiation. *J Org Chem* **70**, 8621–8624 (2005).

7. Via LD, Gia O, Chiarelotto G, Ferlin MG. DNA-targeting pyrroloquinoline-linked butenone and chalcones: Synthesis and biological evaluation. *Eur J Med Chem* **44**, 2854-2861 (2009).

8. Gottumukkala AL*, et al.* Pd-Diimine: A Highly Selective Catalyst System for the Base-Free Oxidative Heck Reaction. *J Org Chem* **76**, 3498–3501 (2011).

9. Jung J-C*, et al.* Efficient Synthesis and Neuroprotective Effect of Substituted 1,3-Diphenyl-2-propen-1-ones. *J Med Chem* **51**, 4054–4058 (2008).

10. Zhang X*, et al.* Pd(OAc)2 Catalyzed Olefination of Highly Electron-Deficient Perfluoroarenes. *J Am Chem Soc* **132**, 4506–4507 (2010).

11. Espenlaub S, Gerster H, Maas G. Synthesis and reactivity of 3-(dialkylamino)allenyl phosphonium salts. PPh3-mediated synthesis of pyrroles from propyne iminium triflates. *ARKIVOC*, 114-131 (2007).
